# Tislelizumab (anti-PD-1) plus chemotherapy as neoadjuvant therapy for patients with stage IB3/IIA2 cervical cancer (NATIC): a prospective, single-arm, phase II study

**DOI:** 10.1038/s41392-025-02294-9

**Published:** 2025-07-04

**Authors:** Jindong Sheng, Haitao Luo, Xiangyu Liu, Chunyan Liu, Wenhao Zhou, Yujie Zhao, Ruoyan Liu, Dan Li, Changxiao Xu, Bo Yang, Ying Liu, Xin Fu, Lewen Bao, Ke Wang, Jihui Hao, Wenxin Liu

**Affiliations:** 1 Department of Gynaecological Oncology, Tianjin Medical University Cancer Institute and Hospital, Tianjin, China; 2 National Clinical Research Center for Cancer, State Key Laboratory of Cancer Prevention and Therapy of Tianjin, Tianjin’s Clinical Research Center for Cancer, Tianjin, China; 3 Shenzhen Engineering Center for Translational Medicine of Precision Cancer Immunodiagnosis and Therapy, YuceBio Technology Co., Ltd, Shenzhen, China; 4 Department of Pathology, Tianjin Medical University Cancer Institute and Hospital, Tianjin, China; 5 Department of Radiology, Tianjin Medical University Cancer Institute and Hospital, Tianjin, China; 6 Pancreatic Center, Tianjin Medical University Cancer Institute and Hospital, National Clinical Research Center for Cancer, State Key Laboratory of Druggability Evaluation and Systematic Translational Medicine, Tianjin Key Laboratory of Digestive Cancer, Tianjin’s Clinical Research Center for Cancer, Tianjin, China

**Keywords:** Gynaecological cancer, Clinical trials

## Abstract

The clinical benefit of neoadjuvant immunochemotherapy in locally advanced cervical cancer (LACC) remains unclear. This single-arm, phase II study (Chinese Clinical Trial Registry, ChiCTR2200065392) aimed to evaluate the efficacy and safety of neoadjuvant anti-programmed cell death protein 1 (PD-1) antibody tislelizumab in combination with chemotherapy in treatment-naïve patients with stage IB3/IIA2 LACC. Enrolled patients received tislelizumab (200 mg, every 3 weeks) plus chemotherapy for 3 cycles before radical surgery. The primary endpoint was the pathological complete response (pCR). Secondary endpoints were objective response rate (ORR) per Response Evaluation Criteria in Solid Tumors version 1.1, disease-free survival, overall survival, and safety. Exploratory endpoints included tissue-based and blood-based biomarkers to identify the biological drivers behind the clinical outcomes. Between November 2022 and March 2024, 30 patients were enrolled. All patients completed 3 cycles of neoadjuvant immunochemotherapy and underwent radical surgery. The pCR was observed in 20 (66.7%) patients, and 4 (13.3%) patients achieved major pathological response (MPR), with an optimal pathological response rate (OPR) of 80.0%. The ORR was 90.0%, with 17 (56.7%) complete responses. Survival data were immature at the median follow-up of 14.7 months (data cutoff, December 31, 2024). Grade 3 treatment-related adverse events (TRAEs) and immune-related AEs occurred in 26.7% and 3.3% of patients, respectively. No treatment-related death occurred. Patients with pCR had significantly higher expression of PD-L1 CPS at baseline, and a strong relationship with immune-related signature (all *p* < 0.05). Neoadjuvant tislelizumab plus chemotherapy showed promising antitumor efficacy and a well-tolerated safety profile in patients with stage IB3/IIA2 LACC, and might be a potential option in this population.

## Introduction

Cervical cancer (CC) ranks the 4th leading cancer in both incidence and mortality among women,^1^ and locally advanced (LACC; stage IB3 and IIA2-IVA) accounts for roughly 37% (median) worldwide.^2^ Although the cisplatin-based concurrent chemoradiotherapy (CCRT) has established the treatment modality for LACC,^3^ approximately 10% of patients with LACC still developed local recurrences, and 30% suffered from distant metastasis after treatment.^4^ Until recently, the combination of immunologic checkpoint inhibitors (ICIs) with chemoradiotherapy (CRT) has emerged as a novel therapeutic option. In contrast to the negative results observed in the CALLA study (durvalumab plus CRT),^5^ the KEYNOTE-A18 trial of pembrolizumab plus CRT resulted in marked enhancements in survival in LACC.^6,7^ Thus, this regimen has been approved for newly diagnosed International Federation of Gynecology and Obstetrics (FIGO) 2014 stage III and IVA LACC patients.

Alternative therapeutic strategies for stage IB2-IIB LACC involve administering platinum-based neoadjuvant chemotherapy,^8^ but patients gaining survival benefits and obtaining improved prognoses from neoadjuvant chemotherapy remains controversial. In the EORTC-55994 study, 5-year OS was assessed for cervical cancer patients (stage IB2-IIB) receiving neoadjuvant chemotherapy or CCRT, yet no superiority of neoadjuvant chemotherapy was observed.^9^ Adding immunotherapy to neoadjuvant chemotherapy is a logical choice in light of multiple clinical data indicating that neoadjuvant immunochemotherapy yields significant clinical benefits in several solid tumors.^10–13^ Several molecular hallmarks—such as high tumor mutational burden (TMB), microsatellite instability (MSI), human papillomavirus (HPV), and high programmed death ligand 1 (PD-L1) expression—provide a strong biological foundation for employing immunotherapy in cervical cancer.^14^ However, evidence for neoadjuvant immunochemotherapy in LACC remains limited. So far, only a phase II NACI study has been published, which evaluated camrelizumab with neoadjuvant chemotherapy in LACC, reported a promising antitumor activity, and a manageable toxicity.^15^

Tislelizumab, a monoclonal immunoglobulin G4 antibody, has been designed to effectively disrupt programmed cell death-1 (PD-1)/PD-L1 binding and minimize binding to Fcγ receptors.^16^ Given its promising anti-tumor effects, tislelizumab has been approved in China for use in several solid tumors.^16^ The clinical benefit observed with neoadjuvant tislelizumab plus chemotherapy had been confirmed in the TD-NICE phase II study for resectable esophageal cancer, showing encouraging antitumor activity (pathological complete response [pCR] of 50%) and acceptable tolerability.^17^

Taken together, this phase II study aimed to assess neoadjuvant therapy of tislelizumab plus chemotherapy in patients with stage IB3/IIA2 LACC.

## Results

### Patient characteristics

**Fig. 1:**
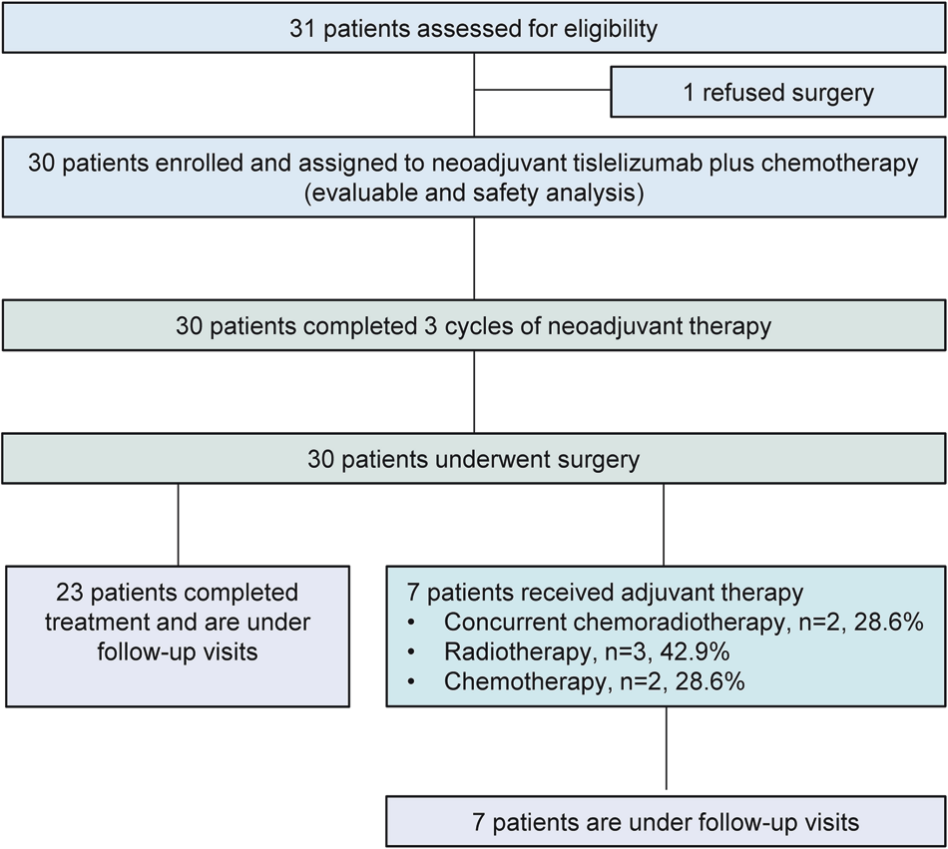
Trial profile

Between November 7, 2022, and March 30, 2024, 31 patients were screened (Fig. 1). One patient, after completing neoadjuvant therapy, refused surgery and opted to withdraw from the study. Thirty patients were evaluated for the efficacy and safety analysis. The median follow-up was 14.7 months (interquartile range [IQR] 11.6–21.4), as of data cutoff (December 31, 2024), and follow-up visits for all patients are ongoing.

**Fig. 2:**
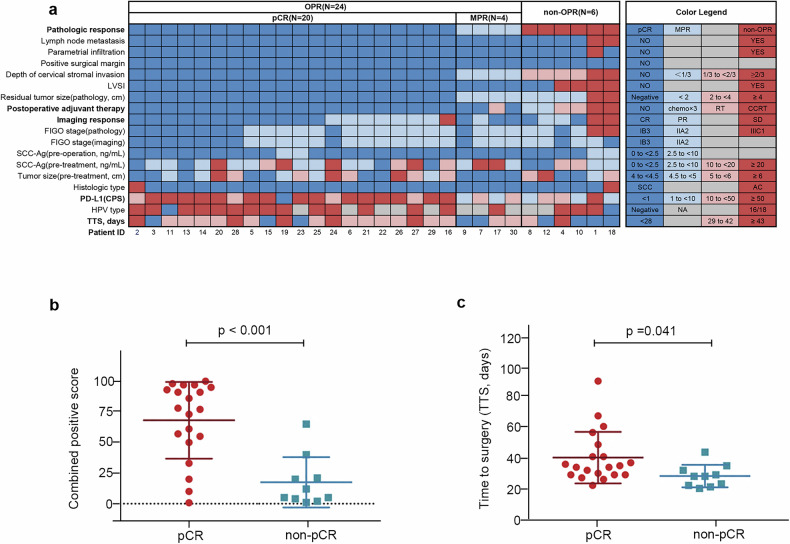
**a** Overview of baseline characteristics, tumor response, and pathological response in 30 patients. Each column represents one patient, with patient IDs listed at the bottom; each row represents a different feature. The definition of different colors is presented in the “Color Legend” on the right. **b** The relationship between PD-L1 CPS and pathological response. **c** The correlation between TTS and pathological response. PD-L1, programmed death ligand 1; CPS combined positive score, TTS time to surgery, SCC squamous-cell carcinoma, OPR optimal pathological response, RT radiotherapy, LVSI lymphatic vascular space invasion, MPR major pathological response, FIGO International Federation of Gynecology and Obstetrics, AC adenocarcinoma, CR complete response, PR partial response. SD stable disease, NA not applicable, HPV human papillomavirus, CCRT concurrent chemoradiotherapy, CPS combined positive score, pCR pathological complete response

The median age was 51.5 years (IQR 39.5–57.0) for all patients, and 80% of patients had an Eastern Cooperative Oncology Group performance status (ECOG PS) score of 0 (24/30). Most patients had squamous cell carcinoma (28/30, 93.3%), FIGO stage IIA2 (19/30, 63.3%), and PD-L1-positive (combined positive scores [CPS] ≥1; 29/30, 96.7%). The median tumor size of 4.6 cm (IQR 4.2–5.2) and the median level of squamous cell carcinoma antigen of 5.2 ng/mL (IQR 3.0–15.0) were observed. The full baseline characteristics are listed in Table 1 and Fig. 2a.

**Table 1:** Baseline characteristics

Patient Characteristics	Patients (*n* = 30)
Age, years	
Median	51.5 (39.5–57.0)
Tumor size, cm	
Median	4.6 (4.2–5.2)
≥4 to <4.5	13 (43.3%)
≥4.5 to <5	5 (16.7%)
≥5 to <6	8 (26.7%)
≥6	4 (13.3%)
Histologic type	
Squamous cell carcinoma	28 (93.3%)
Adenocarcinoma	2 (6.7%)
PD-L1 status	
CPS < 1	1 (3.3%)
1 ≤ CPS < 10	5 (16.7%)
10 ≤ CPS < 50	7 (23.3%)
CPS ≥ 50	17 (56.7%)
ECOG-PS score	
0	24 (80.0%)
1	6 (20.0%)
FIGO 2018 stage	
IB3	11 (36.7%)
IIA2	19 (63.3%)
SCC-Ag, ng/mL	
Median	5.2 (3.0–15.0)
HPV	
16 and/or 18	17 (56.7%)
Negative	2 (6.6%)
Not available	11 (36.7%)

Data are *n* (%) or median (IQR). *HPV* human papillomavirus, *SCC-Ag* squamous cell carcinoma-related antigen, *FIGO* International Federation of Gynecology and Obstetrics, *PD-L1* programmed cell death-1, *CPS* combined positive score, *ECOG-PS* Eastern Cooperative Oncology Group performance status

Among 30 patients, 2 were allergic to paclitaxel and were switched to paclitaxel liposome. Following neoadjuvant therapy, the median time to surgery was 32 days (IQR 28–40). Among 30 patients receiving radical surgery, the median operative time was 135 min (IQR 120–163). For patients under 40 years old (*n* = 8), 5 had one or both ovaries preserved. After surgery, 7 patients (23.3%) received adjuvant therapy (radiotherapy *n* = 3; CCRT *n* = 2; chemotherapy *n* = 2); 6 underwent adjuvant therapy as per the predefined treatment criteria due to not achieving OPR; 1 underwent postoperative localized afterloading brachytherapy for residual cervical intraepithelial neoplasia (CIN) III at the vaginal stump.

### Clinical activity

Of 30 patients, 20 achieved pCR (66.7%; 95% CI 48.8–80.8%), and 4 had a major pathological response (MPR; 13.3%), with an optimal pathological response (OPR) rate of 80.0% (24/30; Table 2 and Fig. 2a). The objective response rate (ORR) was 90.0% (95% CI 74.4–96.5%), with 17 (56.7%) complete response (CR) and 10 (33.3%) partial responses (PR; Table 2). As of data cutoff, 2 patients developed disease recurrence: 1 patient (patient 1) with non-OPR developed peritoneal metastasis at 11 months post-surgery and received comprehensive treatment; 1 patient (patient 7) with MPR experienced a paravaginal recurrence 12 months post-surgery and received radiotherapy. The 18-month disease-free survival (DFS) rate was 90.0% (95% CI 77.7–100.0). No deaths occurred. As the follow-up duration has not yet reached the protocol-specified threshold, overall survival (OS) will be reported in a future publication.

**Table 2:** Tumor and pathologic response to neoadjuvant chemo-immunotherapy

Clinical response to neoadjuvant immunochemotherapy	Patients (*n* = 30)
Imaging-based response per RECIST v.1.1	
Objective response	27 (90.0%)
Best overall response	
Complete response	17 (56.7%)
Partial response	10 (33.3%)
Stable disease	3 (10.0%)
Progressive disease	0
Pathologic response	
Optimal pathologic response	24 (80.0%)
Pathologic complete response	20 (66.7%)
Major pathologic response	4 (13.3%)
Non-optimal pathologic response	6 (20.0%)

Data are *n* (%). *RECIST* Response Evaluation Criteria in Solid Tumors, version 1.1

Only 56.7% of patients (17/30) had an imaging response consistent with the pathological response. Seven patients with PR were assessed as pCR on post-operative pathological evaluation, and 5 with CR were identified with residual viable tumors, including 3 evaluated as MPR and 2 as non-OPR. Among the 3 patients with radiological stable disease (SD), 1 (patient 16) achieved pCR (pathology evaluation revealed that the remaining lesions consisted entirely of necrotic tissue without residual tumor cells); while the other 2 (patient 1 [was squamous cell carcinoma] and patient 18 [was rare gastric-type adenocarcinoma]), initially without no pelvic lymph node metastasis at baseline and on preoperative imaging, were found to have pelvic lymph node involvement in surgical specimens, leading to stage modification to IIIC1p.

Patients who achieved pCR were significantly associated with higher level of PD-L1 CPS (median 77.5 [IQR 53.8–93.5] vs 8.5 [IQR 4.3–20.8]; *p* < 0.001; Fig. 2b); the area under the receiver operating characteristic curve of 0.88 (90% CI 0.75–1.00) was observed. Notably, even 1 patient with a PD-L1 CPS of <1 achieved pCR post-operatively to our regimen. Besides, the proportions of patients achieving pCR were 33.3% (3/9), 80.0% (12/15), and 83.3% (5/6) for time to surgery ≤4 weeks, 4–6 weeks, and >6 weeks, respectively. The patients who achieved pCR were significantly associated with longer time to surgery (median 40.4 vs 28.2 days; *p* = 0.041; Fig. 2c).

### Safety

All 30 patients experienced treatment-related adverse events (TRAEs; Table 3). The most common TRAEs were lymphopenia (27/30, 90.0%), anemia (21/30, 70.0%), and hypoalbuminemia (21/30, 70.0%). Eight patients (26.7%) had grade 3 TRAEs, with no grade 4 reported. There were 7 patients (23.3%) with immune-related adverse events (irAE), and only 1 (3.3%) experienced grade ≥3 irAE. This patient (patient 26) developed peripheral neuropathy (including limb numbness and leg weakness) at 2 weeks post-surgery, progressing to difficulty swallowing and breathing, and was ultimately diagnosed with Guillain-Barré syndrome. Following high-dose corticosteroids, there was a marked improvement in symptoms. During neoadjuvant treatment, none of the observed AEs necessitated treatment discontinuation, dose reduction, or death.

**Table 3:** Treatment-related adverse events in 30 patients

	Any grade	Grade 1–2	Grade 3
**Any treatment-related AEs**	30 (100.0%)	30 (100.0%)	8 (26.7%)
Lymphopenia	27 (90.0%)	23 (76.7%)	4 (13.3%)
Anaemia	21 (70.0%)	21 (70.0%)	0
Hypoproteinemia	21 (70.0%)	21 (70.0%)	0
Leukopenia	16 (53.3%)	16 (53.3%)	0
Neutropenia	13 (43.3%)	12 (40.0%)	1 (3.3%)
Elevated AST	10 (33.3%)	9 (30.0%)	1 (3.3%)
Elevated ALT	8 (26.7%)	6 (20.0%)	2 (6.7%)
Thrombocytopenia	7 (23.3%)	6 (20.0%)	1 (3.3%)
Rash	6 (20.0%)	6 (20.0%)	0
Pain	5 (16.7%)	5 (16.7%)	0
Nausea	5 (16.7%)	5 (16.7%)	0
Loss of appetite	5 (16.7%)	5 (16.7%)	0
Abnormal feeling	4 (13.3%)	4 (13.3%)	0
Itching	4 (13.3%)	4 (13.3%)	0
Vomit	4 (13.3%)	4 (13.3%)	0
Lower extremity venous thrombosis	1 (3.3%)	1 (3.3%)	0
**Immune-related AEs**	7 (23.3%)	7 (23.3%)	1 (3.3%)
Hypothyroidism	5 (16.7%)	5 (16.7%)	0
Guillain⁃Barré syndrome	1 (3.3%)	0	1 (3.3%)
Hyperglycemia	3 (10.0%)	3 (10.0%)	0

Data are *n* (%). *AST* Aspartate Aminotransferase, *ALT* Alanine Aminotransferase, AEs adverse event

Six patients experienced a surgery delay of more than six weeks (the specific reasons for delayed surgery are detailed in the Supplementary materials [supplementary Table S1]). During surgery, the median intraoperative blood loss was 140 mL (IQR 83–178). Four patients (13.3%) experienced at least one post-operative complication, including pelvic lymphocele larger than 5 cm (6.7%), intraoperative vascular injury (3.3%), and post-operative deep venous thrombosis (3.3%). No patients experienced urinary or gastrointestinal injuries during surgery.

### Exploratory endpoint

**Fig. 3:**
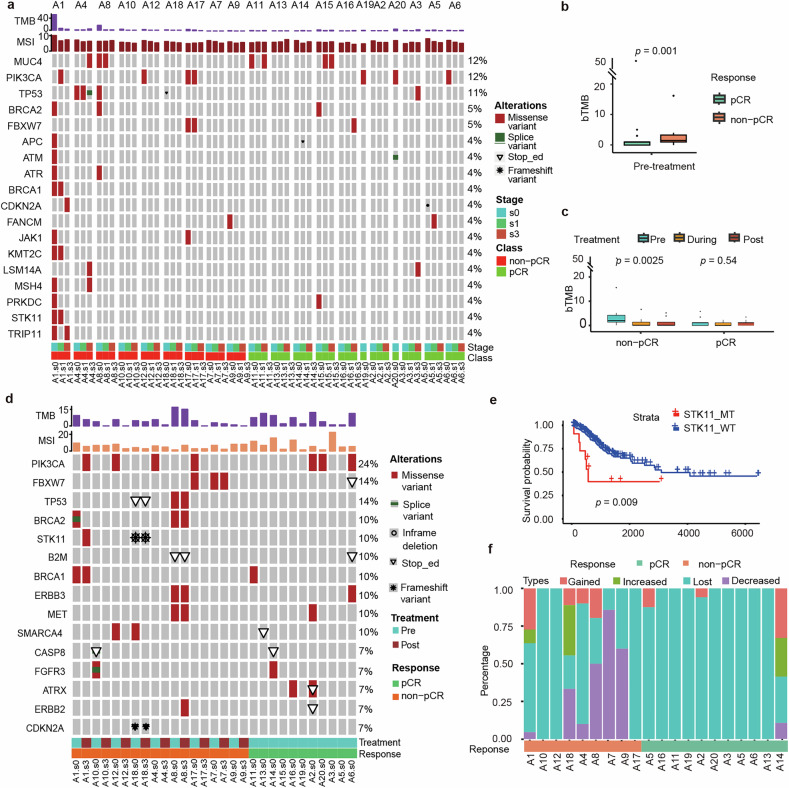
The relationship between genomic mutations and the efficacy of neoadjuvant therapy was explored through next-generation sequencing analysis of blood and tumor tissue samples. **a** Genomic profile at baseline based on ctDNA in 30 patients (mutation frequency ≥4%); **b** Analysis of differences in blood tumor mutation burden (bTMB) among groups at baseline. **c** Changes in bTMB among groups during neoadjuvant therapy. **d** Baseline genomic mutation profile based on tumor tissue samples. **e** The relationship between *STK11* mutations and survival was validated. **f** Changes in gene mutations during neoadjuvant therapy were categorized into four patterns: gain (mutations appearing after treatment), increase (mutation frequency rising after treatment), loss (mutations eliminated after treatment), and decrease (mutation frequency decreasing after treatment). pCR, pathological complete response; bTMB, blood tumor mutational burden

Panel-based targeted sequencing was performed on 60 plasma samples collected from 20 patients. After quality control, we identified that the most frequent genetic alterations in circulating tumor DNA (ctDNA) were *MUC4* (12%), *PIK3CA* (12%), and *TP53* (11%; Fig. 3a). Before treatment, the non-pCR group was significantly associated with higher blood-based TMB (bTMB; Fig. 3b). Next, we dynamically monitored bTMB and found that it showed a significant decrease in the non-pCR group during treatment, whereas bTMB in the pCR group remained relatively stable (Fig. 3c).

To further investigate tumor samples, panel-based targeted sequencing was performed from 20 patients (20 pre- and 15 post-treatment tumor biopsy samples). Somatic mutation analysis showed that the most frequent mutation genes were *PIK3CA*, *FBXW7*, and *TP53* (Fig. 3d), which were consistent with the previous study from the TCGA cervical squamous cell carcinoma and endocervical adenocarcinoma (TCGA-CESC).^18^ A few mutations were observed exclusively in the non-pCR cohort, including the *STK11* mutation. Further analysis using the TCGA-CESC dataset to examine the significant association between somatic *STK11* mutation and poorer OS compared to *STK11* wild type (Fig. 3e). Next, we explored the prognostic value of biomarkers of interest. No significant difference was observed in tissue-based TMB, MSI, and intratumoral heterogeneity (ITH; all *p* > 0.05; supplementary Fig. 1a**)** pre-treatment. The non-pCR group was associated with significantly lower tissue-based TMB and ITH levels post-treatment (all *p* < 0.05; supplementary Fig. 1b). Besides, post-treatment (Fig. 3f), patients with non-pCR had a tendency to acquire new mutations or increase mutation frequency compared to pCR groups (44% vs 27%); meanwhile, most patients with pCR exhibited mutation loss (elimination of mutations after treatment), whereas non-pCR patients showed a reduction in mutations (decreased mutation frequency after treatment; 67%). No significant difference in gene mutation alterations during neoadjuvant therapy was observed between the two pathologic response groups (supplementary Fig. 1c).

**Fig. 4:**
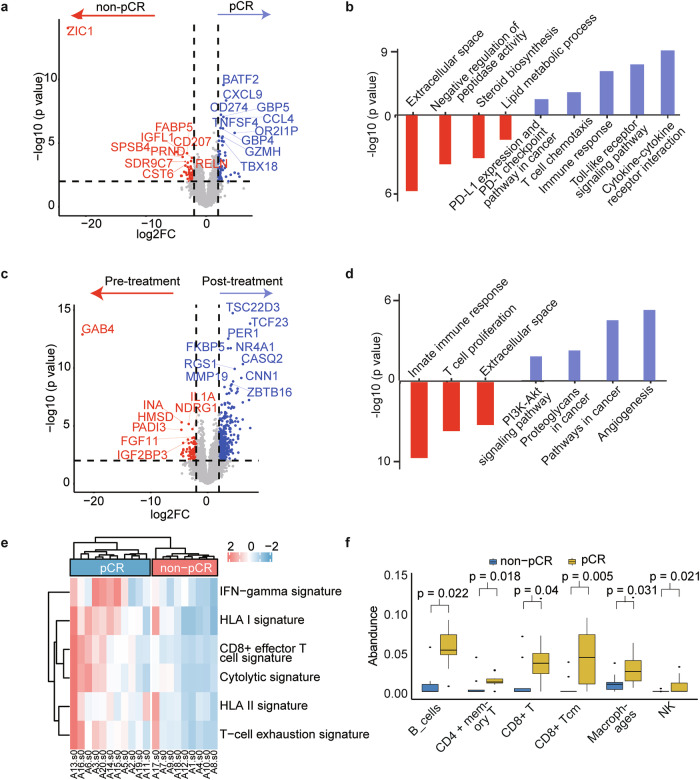
Comparison of differential genes, immune signals, and tumor microenvironment between different response groups. **a** Overview of differentially expressed genes (DEGs) at baseline. **b** Pathway enrichment analysis at baseline among groups. **c** DEGs before and after treatment for patients with non-pCR. **d** Pathway enrichment analysis before and after treatment in the non-pCR group. **e** Heatmap focusing on the immune signatures of baseline pCR and non-pCR groups. **f** Differences in immune molecules at baseline among groups. pCR pathological complete response

Subsequently, transcriptomic profiling was conducted to compare gene expression profiles based on different pathological response states. We performed corrections for multiple hypothesis testing on the Differentially expressed genes (DEGs; supplementary Fig. 2). Pre-treatment, 115 DEGs were identified (Fig. 4a), and *BATF2*, *CXCL9*, and *CD274* genes were highly expressed in patients with pCR. The upregulated genes in the pCR group were particularly associated with the pathways of cytokine-cytokine receptor interaction, immune response, and the PD-L1/PD-1 signaling pathway; in contrast, the upregulated genes in the non-pCR group were primarily enriched in extracellular space, negative regulation of peptidase activity, and lipid metabolic processes (Fig. 4b). Further analysis of DEGs (Fig. 4c) in the non-pCR group pre- and post-treatment identified 479 DEGs (89 upregulated pre-treatment and 390 upregulated after treatment). Pathway enrichment analysis showed that tumor-associated pathways were not inhibited post-treatment in non-pCR groups. Conversely, immune-related pathways, including T-cell proliferation and innate immune responses, were suppressed (Fig. 4d).

Finally, we evaluated immune signatures between different response groups. Pre-treatment, immune signatures revealed that the pCR group had better levels of T-cell exhaustion, IFN-γ, and cytolytic activity compared to the non-pCR group (Fig. 4e). Similarly, by using data from the TCGA-CESC dataset, we found that higher CD8^+^ effector T and cytolytic signatures were associated with longer OS (supplementary Fig. 3). Then, we investigated changes in immune cells at baseline and during neoadjuvant therapy across different response groups. Pre-treatment, we found pCR group was associated with significantly higher abundance of immune cells, such as B cells (Fig. 4f). In the non-pCR group, the abundance of effector TH1 cells significantly decreased during neoadjuvant therapy, whereas the abundance of M2 macrophages significantly increased (all *p* < 0.05), and there was an increased trend in regulatory T cells (Tregs; supplementary Fig. 4).

## Discussion

The NATIC study evaluated neoadjuvant tislelizumab plus chemotherapy for patients with stage IB3/IIA2 LACC and met its primary endpoint, with a pCR rate of 66.7% and OPR of 80.0%. Besides, the ORR was 90.0%, with 56.7% of patients achieving CR. This combinational neoadjuvant therapy exhibited a manageable and acceptable safety profile, with a low incidence ( ≥3 grade TRAEs, 26.7%) of AEs. The combination regimens may be a promising treatment option for stage IB3/IIA2 LACC.

So far, extensive evidence has demonstrated that post-neoadjuvant pCR is a surrogate marker for long-term survival prognosis.^19,20^ Accordingly, pCR was defined as the primary efficacy endpoint in our study. Among the 30 surgical patients who underwent surgery, an encouraging pCR of 66.7% was observed. Comparatively, platinum-based neoadjuvant chemotherapy reported pCR rates ranging from 8.5% to 26% in the contemporary trials,^20–22^ which was obviously lower than that in our study. The OPR and MPR were also chosen as additional pathological endpoints in our study. Compared to Huang et al., who conducted a retrospective study assessing IB2-IIB cervical cancer receiving neoadjuvant chemotherapy (7.5–9.3%), our neoadjuvant immunochemotherapy study achieved a higher OPR of 80%, with 4 patients (13.3%) achieving an MPR.^23^ This chemo-immunotherapy strategy has also been explored in the two randomized phase 3 studies (KEYNOTE-A18 and CALLA trials), both of which added ICIs to CRT in LACC.^5,6^ However, differences in the patient population and ICIs employed have led to conflicting results, with pembrolizumab plus CRT receiving approval and durvalumab plus CRT failing to meet its primary endpoint.^24^ Two contemporary trials assessed neoadjuvant immunotherapy in LACC. Liu et al. assessed neoadjuvant sintilimab plus chemotherapy (for 3 cycles) prior to surgery for LACC and reported a pCR rate of 36.2%.^25^ A published NACI study evaluated camrelizumab plus neoadjuvant chemotherapy for LACC, reporting a pCR of 38%.^15^ Although caution is warranted when comparing across studies, the nearly doubled pCR rate (66.7%) observed in our study is particularly promising, given the historically poor pCR outcomes observed in the above studies. The higher pCR in our study may be related to the patients with a longer duration of treatment (3 cycles in our study vs. 2 cycles in the NACI study) and the lower disease stage (all patients in our study were IB3/IIA2, compared to 60% of patients in the NACI study being at stages IIB/IIIC1r). Besides, the ORR in our study was somewhat lower (97.7–98% vs 90%) when compared with the aforementioned two studies.^15,25^ Despite encouraging pathological responses, disease recurrence was observed in 2 patients, suggesting that the long-term prognostic impact of neoadjuvant immunotherapy in LACC requires further validation through extended follow-up and larger studies.

Several studies have investigated induction chemotherapy or immunotherapy prior to definitive CRT for LACC.^26,27^ The INTERLACE trial demonstrated that adding induction chemotherapy to CRT led to improvements in survival.^26^ The COLIBRI trial supported the efficacy and safety of administering Inivolumab plus ipilimumab as induction therapy followed by CRT in patients with LACC, with an ORR of 97.5%.^27^ Besides, the EMBRACE-I trial consisted of chemoradiotherapy followed by MRI-based IGABT in LACC, achieving durable and effective long-term local control.^28^ However, the patient populations included in these studies were predominantly at advanced stages (FIGO IIB-IV, approximately 50–84%), whereas our study specifically targeted patients with earlier-stage disease (IB3/IIA2), an underrepresented subgroup. The promising outcomes of our study support the hypothesis that neoadjuvant immunochemotherapy followed by surgery can effectively improve pathological responses in IB3/IIA2 LACC. Despite these encouraging results, the questions of the optimal sequencing of immunotherapy, chemotherapy, and surgery or CRT remain unanswered and need further investigation to maximize the therapeutic benefit while minimizing toxicity.

In this phase II study, a discrepancy between imaging and pathologic response was observed, with only 56.7% of patients showing consistency between imaging and pathological findings. At the same time, some patients who were assessed as PR (n = 7) or SD (n = 1) by pre-operative imaging could also achieve pCR post-surgery. Conversely, 5 patients who achieved CR pre-operatively also found residual tumor tissue upon surgical examination. This is consistent with study showing that pathological response poorly correlates with residual active invasive tumors in clinical complete responders in pelvic gynecological tumors following neoadjuvant chemotherapy.^29,30^ Meanwhile, Ditto et al. found the overall accuracy of MRI in the pre-operative staging in cervical cancer post-neoadjuvant therapy was suboptimal, similar to our study.^31^ The reasons for the discrepancy may be that MRI has inherent limitations in distinguishing necrotic tissue, inflammatory changes due to neoadjuvant therapy, and viable neoplastic tissue; additionally, the use of more radical surgery (such as type C1 hysterectomy) compared to conventional approaches could uncover small disease foci missed by imaging.^32^ Besides, subgroup analysis identified a correlation between PD-L1 expression (CPS) and pathological response, with significantly higher PD-L1 CPS observed in the pCR group (*p* < 0.001). This finding supports the idea that the overexpression of PD-L1 may predict a better clinical benefit to ICIs.^17^ Notably, the patient in our study with a PD-L1 CPS of <1 also achieved a pCR following radical surgery.

The data on the optimal time to surgery following neoadjuvant immunochemotherapy are sparse. We found an intriguing observation of a significant difference between the time to surgery and pathological response (*p* = 0.041); patients who underwent delayed surgery (time to surgery >6 weeks) had the highest pCR rate, reaching 83.3%. These results suggest that performing surgery 4–6 weeks or even later after neoadjuvant immunochemotherapy might lead to improved pathological responses. This is consistent with Chen et al., who found a trend toward improved MPR and pCR among patients receiving delayed surgery in neoadjuvant immunochemotherapy.^33^ It may have been driven by the distinct characteristic of the ICIs, which differ from conventional chemotherapeutic agents. Although responses to chemotherapies are observed within a few weeks of initiation, immunotherapy generally produces a more delayed therapeutic effect.^34^ One possibility is that immunotherapy may rely on the activation of pre-existing tumor-reactive T cell pool or promote the generation of new antitumor T cell responses, thus delaying tumor shrinkage due to the time needed for effector T cell activation and function—commonly observed in patients receiving ICIs.^35^ Besides, allowing a moderate delay before surgery offers logistical advantages, such as improved coordination with gynecologic oncologists and adequate time for preoperative assessments and staging, ultimately enhancing the quality of clinical management.^36^ However, given the limited sample size in our study, these results warrant cautious interpretation, and further investigations are needed to validate it.

Exploratory biomarker analysis showed that non-pCR group was associated with significantly higher baseline bTMB, suggesting that ctDNA detection can provide valuable insights into predicting treatment efficacy. Regarding the tissue-based biomarker analysis, the non-pCR group exhibited significant post-treatment decreases in TMB and ITH compared to pre-treatment. This suggests that during neoadjuvant immunochemotherapy, tumor cells harboring somatic mutations, as well as a substantial proportion of tumor-cell subclones, are effectively eliminated. We also found that *STK11* gene mutations were exclusively present in the non-pCR group, and validation using the external TCGA-CESC cohort revealed that patients with *STK11* mutations had significantly poorer OS. The previous study had shown that *STK11* mutations in lung cancer lead to a “cold” tumor immune microenvironment (TME).^37^ Functional loss of *STK11* can disrupt the lactate transporter MCT4, increasing lactate production and export in the tumor microenvironment; excess lactate promotes macrophage polarization to the M2 phenotype, reducing T-cell infiltration and function, which can contribute to immune escape.^38^ Taken together, this suggests neoadjuvant immunochemotherapy in cervical cancer may be more reliant on changes in the characteristics of the TME. DEG analysis revealed that gene such as *CXCL9* was highly expressed in the pCR group. This is consistent with the recent study that found *CXCL9* in the TME playing a crucial role in promoting T-cell infiltration, contributing to a “hot” TME, and enhancing the clinical response to immunotherapy.^39^ Pathway enrichment analysis indicated that genes upregulated in the pCR group were significantly associated with immune-related pathways; in contrast, the non-pCR group had genes upregulated in biological pathways but did not show effective immune pathway activation post-treatment. These findings suggest that before treatment, patients with pCR possess a more active TME at baseline, while patients with non-pCR lack high expression of immune-related genes and did not potentiate TME with neoadjuvant therapy.

Further analysis of immune-related signatures in baseline tumor tissues showed that the pCR group had higher levels for cytolytic signature, CD8^+^ effector T cells, T-cell exhaustion, and IFN-γ signatures, indicating a more “hot” TME.^40^ This suggests that combining ICIs with chemotherapy may offer better clinical outcomes.^41,42^ IFN-γ can inhibit tumor angiogenesis and activate macrophages to suppress tumor growth and its signature might be an important factor related to clinical prognosis.^43,44^ Besides, baseline immunological assessments could further guide more effective stratification and objective exploration in subsequent randomized studies. We speculate that IFN-γ may be a biomarker that impacts the efficacy of neoadjuvant therapy and might induce an effective and durable antitumor immune response. Future study needs to explore its potential mechanisms. Additionally, immune cell abundance analysis revealed that the pCR group had significantly higher levels of immune cells. This finding is consistent with higher immune gene expression levels observed in baseline samples of the pCR group. Conversely, the non-pCR group showed a significant decrease in Th1 cell abundance and an increase in M2 macrophages and Tregs during treatment. Th1 cells are essential for macrophage activation and enhancing the efficacy of PD-1/PD-L1 checkpoint blockade.^45^ Tregs suppress T-cell cytotoxicity, leading to an immunosuppressive environment.^46^ The decreased Th1 expression and increased Tregs in the non-pCR group may contribute to ineffective immunotherapy, though the mechanisms driving these changes with neoadjuvant treatment require further investigation. Taken together, this bedside-to-bench iterative process illustrates the contributions of our translational analyses. From clinical outcomes to laboratory analyses, the molecular insights not only validate the observed clinical responses but also reveal the biological mechanisms underlying the potential benefits of neoadjuvant immunochemotherapy followed by surgery.

Overall, the present study showed a well-tolerated and manageable safety profile for neoadjuvant tislelizumab plus chemotherapy in LACC patients. Safety is consistent with the established toxicity of each agent,^16,47^, and no unexpected safety signals were observed. Most TRAEs were related to chemotherapy. Grade 3 TRAEs occurred in 30% of patients, with no grade 4 TRAEs seen, which is lower than the previously reported rate of 40%.^15^ Additionally, only 1 patient developed a grade ≥3 irAE. Furthermore, surgical complications were minimal at 13.3%, lower than the 42% reported in the NACI study.^15^

Several limitations of our study warrant consideration. First, the single-arm nature of this trial, conducted at a single center with a relatively small sample size, may introduce potential bias. However, given the promising clinical data, larger randomized controlled trials are planned for the future. Second, the short duration of follow-up has resulted in immature survival data. Third, our primary endpoint pCR is just a surrogate endpoint, and whether patients with pCR can achieve better long-term survival requires further longer follow-up. Finally, post-operative follow-up schedules and adjuvant therapy regimens require further investigation to establish optimal strategies.

To sum up, this study meets the prespecified endpoints showing promising pathological response and well-tolerated safety of neoadjuvant tislelizumab plus chemotherapy in LACC. This combination regimen of tislelizumab plus chemotherapy has the potential to be a feasible neoadjuvant regimen for patients with LACC. The long-term survival outcomes remain pending.

## Materials and methods

### Study design and participants

The NATIC study was a single-center, single-arm, open-label, investigator-initiated, phase II study. This trial adhered to the Declaration of Helsinki and Good Clinical Practice Guidelines and was registered at the Chinese Clinical Trial Registry (Registration Number: ChiCTR2200065392). Ethical approval was obtained from the institutional review board of Tianjin Medical University Cancer Institute and Hospital (Ethics number: E20220941). Written informed consent was required to be provided.

Eligible patients aged 18–65 years had locally advanced, histologically confirmed squamous cell carcinoma, adenocarcinoma, or adenosquamous carcinoma of the cervix with stage IB3, IIA2 disease (tumor diameter of ≥4 cm) as per International FIGO (2018). Additional inclusion criteria included patients with an ECOG PS score of 0 or 1, adequate organ function, without previous anti-tumor treatment, and at least one measurable lesion per Response Evaluation Criteria in Solid Tumors (RECIST) v1.1. Patients were deemed ineligible if they had a history of primary malignancy within the previous 5 years, had an active autoimmune disease that needed systemic treatment, or received systemic corticosteroids or immunosuppressive agents within 14 days before enrolment (the details of inclusion and exclusion criteria are provided in the Supplementary materials).

### Procedures

Patients received three 21-day cycles of pre-operative neoadjuvant therapy, including tislelizumab (200 mg, day 1), paclitaxel (175 mg/m^2^, day 1), and platinum (carboplatin AUC = 5 or cisplatin 60 mg/m^2^, day 2 of cycle 1 and day 1 of cycles 2–3). After 3–4 weeks of neoadjuvant therapy, patients who had imaging evaluation as CR, PR, or SD per RECIST v1.1 would proceed with radical hysterectomy (type C1) plus pelvic lymphadenectomy and para-aortic lymphadenectomy (to the level of the inferior mesenteric artery). Besides, ovarian preservation could be considered for patients <40 years old with squamous cell carcinoma, based on their intention. After that, patients could receive adjuvant chemotherapy, radiotherapy, or CCRT. A detailed treatment process is provided in the Supplementary materials. The follow-up was scheduled every 3 months during the first year, after completing the treatment, and then every 6 months throughout the second year.

Dose modifications for tislelizumab were not permitted; however, interruptions for up to 8 weeks were allowed in the event of irAEs, at the investigator’s discretion. Modifications to chemotherapy dosing were made in accordance with prescribing information, institutional protocols, or local treatment guidelines (Supplementary materials). Patients would discontinue the study treatment and switch to other appropriate treatments when disease progression or intolerable toxicity occurred before surgery.

### Assessments

Investigator-based imaging assessment (supplementary Fig. 5a) according to the RECIST v.1.1 at baseline, before each neoadjuvant therapy cycle, pre-surgery, and follow-up visit. All imaging assessments were performed by two independent radiologists. Baseline magnetic resonance imaging (MRI) assessment was preferred, but computed tomography (CT) was used as an alternative approach in patients with contraindications (See Supplementary materials for details). Post-operative pathological evaluation (supplementary Fig. 5b) was conducted by two experienced pathologists. AEs were documented during the neoadjuvant period and within 90 days before the end of treatment by evaluating clinical laboratory tests (blood biochemical, complete blood count, and electrocardiogram), physical examination, and vital sign assessment. Safety was graded according to the National Cancer Institute Common Terminology Criteria for Adverse Events (CTCAE) v5.0.

### Biomarker analysis

Plasma samples were obtained at three time points during treatment: before neoadjuvant therapy (pre-treatment sample), before the administration of the second cycle (on-treatment sample), and before surgery (post-treatment sample). Tumor tissue samples were also acquired from pre-treatment and post-surgery surgical specimens. Next-generation sequencing (NGS) was employed for genomic and transcriptomic analysis. Genomic DNA from tissue was extracted from formalin-fixed paraffin-embedded (FFPE) biopsies and matched peripheral blood samples. Somatic variants were identified by Vardict, and the results were annotated with snpEff. TMB was determined by the number of nonsynonymous mutations per megabase, and MSI was assessed with an MSI sensor. Copy number variants (CNVs) were detected with AscatNGS, and ITH was estimated using PyClone. For transcriptomic analysis, total RNA was extracted from tumor samples using RNeasy Plus Universal Kits, and RNA sequencing (RNA-seq) libraries were prepared using the rRNA depletion module and NadPrep DNA Library Preparation Module. Gene expression was quantified using RSEM, while differential expression analysis was conducted with DESeq2. Enrichment analysis was conducted using KOBAS. Immune signatures and abundance of infiltrating immune cells were assessed by gene expression matrix. Immunohistochemistry for PD-L1 was also performed using commercially available kits. Details are in the Supplementary materials.

### Outcomes

The primary endpoint was pathological complete response (pCR, i.e., the complete absence of residual viable tumor cells in both the primary lesion and lymph nodes). Secondary endpoints included MPR (i.e., no more than 10% residual viable tumor and ≤3 mm cervical stromal invasion in depth), OPR (including pCR and MPR), objective response rate (ORR, i.e., the proportion of patients achieving either a CR or PR) as assessed by investigator according to RECIST v1.1, DFS (i.e., the time from study entry until the occurrence of progression, either locoregional or distant recurrence, or death from any cause), and OS (i.e., the duration from enrolment to death due to any reason), and safety. Pre-defined exploratory endpoints encompassed the subgroup analysis and biomarker analysis. Subgroup analyses explored correlations between the imaging-based response post-neoadjuvant therapy and subsequent pathological outcomes, as well as the association between pathological response (pCR versus non-pCR) and variables such as baseline PD-L1 CPS and the interval from neoadjuvant therapy to surgery. Biomarker analysis aimed to uncover predictive indicators for the efficacy of immunochemotherapy in the neoadjuvant setting and to monitor immunological alterations in both peripheral blood and tumor specimens throughout the treatment course.

### Statistical analysis

Simon’s optimal two-stage design was employed to evaluate the primary endpoint. Based on the historical pCR rates of 20%,^9^ derived from previous studies and clinical experience, a threshold of 45% was set to define treatment efficacy. Assuming *α* = 0.025 and *β* = 0.20, a total sample size of 30 patients was determined across two stages. In stage 1, 10 patients were enrolled and assessed for pathological response; if at least 2 achieved pCR, the trial proceeded to stage 2 with an additional 20; if no more than 10 pCR were observed, the neoadjuvant regimen was deemed ineffective.

Efficacy or safety analyses were conducted on all participants who were treated with at least one dose of the investigational drug and evaluable efficacy or safety assessment. The 95% CIs for both the pCR rate and ORR were estimated using the Clopper-Pearson method. Group comparisons were performed using non-parametric tests, such as the Mann-Whitney U test or the Wilcoxon signed rank test. The algorithm xCell was used to evaluate immune cell abundance in all samples. Exploratory analyses were generated using R software (v4.0.0) with appropriate packages (e.g., pheatmap, ggplot2) and GraphPad Prism 8 (used for additional statistical analyses and data visualization). A two-sided p-value of less than 0.05 was considered statistically significant; SPSS software (version 275.0, SPSS Inc., IL, USA) was employed for statistical analyses.

## Supplementary information

Supplementary_Materials

Protocol

## Data Availability

The raw sequencing datasets have been submitted to the Genome Sequence Archive (GSA; under Accession number HRA011701) at the National Genomics Data Center. The clinical data supporting the findings of this study are available from the corresponding authors upon reasonable request.

## References

[1] Bray, F. et al. Global cancer statistics 2022: GLOBOCAN estimates of incidence and mortality worldwide for 36 cancers in 185 countries. *CA Cancer J. Clin.* **74**, 229–263 (2024). 38572751 10.3322/caac.21834

[2] Monk, B. J. et al. Proportions and incidence of locally advanced cervical cancer: a global systematic literature review. *Int J. Gynecol. Cancer* **32**, 1531–1539 (2022). 36241221 10.1136/ijgc-2022-003801 PMC9763192

[3] Morris, M. et al. Pelvic radiation with concurrent chemotherapy compared with pelvic and para-aortic radiation for high-risk cervical cancer. *N. Engl. J. Med* **340**, 1137–1143 (1999). 10202164 10.1056/NEJM199904153401501

[4] Nomden, C. N. et al. Nodal failure after chemo-radiation and MRI guided brachytherapy in cervical cancer: Patterns of failure in the EMBRACE study cohort. *Radiother. Oncol.* **134**, 185–190 (2019). 31005214 10.1016/j.radonc.2019.02.007

[5] Monk, B. J. et al. Durvalumab versus placebo with chemoradiotherapy for locally advanced cervical cancer (CALLA): a randomised, double-blind, phase 3 trial. *Lancet Oncol.* **24**, 1334–1348 (2023). 38039991 10.1016/S1470-2045(23)00479-5

[6] Lorusso, D. et al. Pembrolizumab or placebo with chemoradiotherapy followed by pembrolizumab or placebo for newly diagnosed, high-risk, locally advanced cervical cancer (ENGOT-cx11/GOG-3047/KEYNOTE-A18): a randomised, double-blind, phase 3 clinical trial. *Lancet* **403**, 1341–1350 (2024). 38521086 10.1016/S0140-6736(24)00317-9

[7] Lorusso, D. et al. Pembrolizumab or placebo with chemoradiotherapy followed by pembrolizumab or placebo for newly diagnosed, high-risk, locally advanced cervical cancer (ENGOT-cx11/GOG-3047/KEYNOTE-A18): overall survival results from a randomised, double-blind, placebo-controlled, phase 3 trial. *Lancet* **404**, 1321–1332 (2024). 39288779 10.1016/S0140-6736(24)01808-7

[8] National Comprehensive Cancer Network Cervical Cancer Guideline Version 3.2024. Cervical Cancer. https://www.nccn.org/guidelines/guidelines-detail?category=1&id=1426 (2025).

[9] Kenter, G. G. et al. Randomized Phase III study comparing neoadjuvant chemotherapy followed by surgery versus Chemoradiation in Stage IB2-IIB Cervical Cancer: EORTC-55994. *J. Clin. Oncol.* **41**, 5035–5043 (2023). 37656948 10.1200/JCO.22.02852

[10] Loibl, S. et al. Neoadjuvant durvalumab improves survival in early triple-negative breast cancer independent of pathological complete response. *Ann. Oncol.* **33**, 1149–1158 (2022). 35961599 10.1016/j.annonc.2022.07.1940

[11] Verschoor, Y. L. et al. Neoadjuvant atezolizumab plus chemotherapy in gastric and gastroesophageal junction adenocarcinoma: the phase 2 PANDA trial. *Nat. Med* **30**, 519–530 (2024). 38191613 10.1038/s41591-023-02758-x PMC10878980

[12] Yi, J. S. et al. Immune activation in early-stage non-small cell lung cancer patients receiving neoadjuvant chemotherapy plus Ipilimumab. *Clin. Cancer Res* **23**, 7474–7482 (2017). 28951518 10.1158/1078-0432.CCR-17-2005 PMC5732888

[13] Chen, G. et al. Neoadjuvant PD-1 blockade with sintilimab in mismatch-repair deficient, locally advanced rectal cancer: an open-label, single-centre phase 2 study. *Lancet Gastroenterol. Hepatol.* **8**, 422–431 (2023). 36870360 10.1016/S2468-1253(22)00439-3

[14] Monk, B. J. et al. Integration of immunotherapy into treatment of cervical cancer: Recent data and ongoing trials. *Cancer Treat. Rev.* **106**, 102385 (2022). 35413489 10.1016/j.ctrv.2022.102385 PMC10697630

[15] Li, K. et al. Neoadjuvant chemotherapy plus camrelizumab for locally advanced cervical cancer (NACI study): a multicentre, single-arm, phase 2 trial. *Lancet Oncol.* **25**, 76–85 (2024). 38048802 10.1016/S1470-2045(23)00531-4

[16] Desai, J. et al. Phase IA/IB study of single-agent tislelizumab, an investigational anti-PD-1 antibody, in solid tumors. *J. Immunother. Cancer* **8**, e000453 (2020). 32540858 10.1136/jitc-2019-000453 PMC7295442

[17] Yan, X. et al. Tislelizumab combined with chemotherapy as neoadjuvant therapy for surgically resectable esophageal cancer: A prospective, single-arm, phase II study (TD-NICE). *Int J. Surg.* **103**, 106680 (2022). 35595021 10.1016/j.ijsu.2022.106680

[18] Integrated genomic and molecular characterization of cervical cancer. *Nature* **543**, 378–384 (2017). 10.1038/nature21386 PMC5354998 28112728

[19] Zhu, Y., Yang, J., Zhang, X., Chen, D. & Zhang, S. Acquired treatment response from neoadjuvant chemotherapy predicts a favorable prognosis for local advanced cervical cancer: A meta-analysis. *Med. (Baltim.)* **97**, e0530 (2018). 10.1097/MD.0000000000010530 PMC5944488 29703026

[20] Candelaria, M. et al. Prognostic significance of pathological response after neoadjuvant chemotherapy or chemoradiation for locally advanced cervical carcinoma. *Int Semin. Surg. Oncol.* **3**, 3 (2006). 16457727 10.1186/1477-7800-3-3 PMC1386679

[21] Hu, Y. et al. Neoadjuvant chemotherapy for patients with International Federation of Gynecology and Obstetrics stages IB3 and IIA2 cervical cancer: a multicenter prospective trial. *BMC Cancer* **22**, 1270 (2022). 36471257 10.1186/s12885-022-10355-3 PMC9724322

[22] Angioli, R. et al. Neoadjuvant chemotherapy plus radical surgery followed by chemotherapy in locally advanced cervical cancer. *Gynecol. Oncol.* **127**, 290–296 (2012). 22819938 10.1016/j.ygyno.2012.07.104

[23] Huang, K. et al. Optimal pathological response indicated better long-term outcome among patients with stage IB2 to IIB cervical cancer submitted to neoadjuvant chemotherapy. *Sci. Rep.* **6**, 28278 (2016). 27325186 10.1038/srep28278 PMC4915007

[24] Duska, L. R., Podwika, S. E. & Randall, L. M. Top advances of the year: Cervical cancer. *Cancer* **130**, 2571–2576 (2024). 38651760 10.1002/cncr.35334

[25] Wan, T. et al. Efficacy and safety of sintilimab plus paclitaxel and cisplatin as neoadjuvant therapy for locally advanced cervical cancer: A phase II trial. *J. Clin. Oncol.* **42**, 5512–5512 (2024).

[26] McCormack, M. et al. Induction chemotherapy followed by standard chemoradiotherapy versus standard chemoradiotherapy alone in patients with locally advanced cervical cancer (GCIG INTERLACE): an international, multicentre, randomised phase 3 trial. *Lancet* **404**, 1525–1535 (2024). 39419054 10.1016/S0140-6736(24)01438-7

[27] Ray-Coquard, I. L. et al. In situ immune impact of nivolumab + ipilimumab combination before standard chemoradiation therapy (RTCT) for FIGO IB3-IVA in patients (pts) with cervical squamous carcinoma: COLIBRI trial, a GINECO study. *J. Clin. Oncol.* **41**, 5501–5501 (2023).37847874

[28] Pötter, R. et al. MRI-guided adaptive brachytherapy in locally advanced cervical cancer (EMBRACE-I): a multicentre prospective cohort study. *Lancet Oncol.* **22**, 538–547 (2021). 33794207 10.1016/S1470-2045(20)30753-1

[29] Li, R. et al. Prognostic value of responsiveness of neoadjuvant chemotherapy before surgery for patients with stage IB(2)/IIA(2) cervical cancer. *Gynecol. Oncol.* **128**, 524–529 (2013). 23146686 10.1016/j.ygyno.2012.11.006

[30] Chung, Y. S. et al. Prognostic value of complete metabolic response on ^18^F-FDG-PET/CT after three cycles of neoadjuvant chemotherapy in advanced high-grade serous ovarian cancer. *J. Gynecol. Oncol.* **33**, e28 (2022). 35128858 10.3802/jgo.2022.33.e28 PMC9024185

[31] Ditto, A. et al. Diagnostic accuracy of magnetic resonance imaging in the pre-operative staging of cervical cancer patients who underwent neoadjuvant treatment: a clinical-surgical-pathologic comparison. *Cancers* **15**, 2061 (2023). 37046722 10.3390/cancers15072061 PMC10093554

[32] Namura, M. et al. Discrepancies between pathological tumor responses and estimations of complete response by magnetic resonance imaging after neoadjuvant chemotherapy differ by breast cancer subtype. *Clin. Breast Cancer* **18**, 128–134 (2018). 28843513 10.1016/j.clbc.2017.07.001

[33] Chen, J., Deng, H., He, J., Wang, Z. & Li, S. Impact of the interval between neoadjuvant immunochemotherapy and surgery on surgical-pathological outcomes in non-small cell lung cancer. *Front Oncol.* **12**, 909726 (2022). 36158657 10.3389/fonc.2022.909726 PMC9491272

[34] Pardoll, D. M. The blockade of immune checkpoints in cancer immunotherapy. *Nat. Rev. Cancer* **12**, 252–264 (2012). 22437870 10.1038/nrc3239 PMC4856023

[35] Oliveira, G. & Wu, C. J. Dynamics and specificities of T cells in cancer immunotherapy. *Nat. Rev. Cancer* **23**, 295–316 (2023). 37046001 10.1038/s41568-023-00560-y PMC10773171

[36] AlHilli, M. M., Elson, P., Rybicki, L., Khorana, A. A. & Rose, P. G. Time to surgery and its impact on survival in patients with endometrial cancer: A National Cancer Database study. *Gynecol. Oncol.* **153**, 511–516 (2019). 31000472 10.1016/j.ygyno.2019.03.244

[37] Rizvi, H. et al. Molecular determinants of response to Anti-Programmed Cell Death (PD)-1 and Anti-Programmed Death-Ligand 1 (PD-L1) blockade in patients with non-small-cell lung cancer profiled with targeted next-generation sequencing. *J. Clin. Oncol.* **36**, 633–641 (2018). 29337640 10.1200/JCO.2017.75.3384 PMC6075848

[38] Qian, Y. et al. MCT4-dependent lactate secretion suppresses antitumor immunity in LKB1-deficient lung adenocarcinoma. *Cancer Cell* **41**, 1363–1380.e1367 (2023). 37327788 10.1016/j.ccell.2023.05.015 PMC11161201

[39] Hoch, T. et al. Multiplexed imaging mass cytometry of the chemokine milieus in melanoma characterizes features of the response to immunotherapy. *Sci. Immunol.* **7**, eabk1692 (2022). 35363540 10.1126/sciimmunol.abk1692

[40] Liu, Y. T. & Sun, Z. J. Turning cold tumors into hot tumors by improving T-cell infiltration. *Theranostics* **11**, 5365–5386 (2021). 33859752 10.7150/thno.58390 PMC8039952

[41] Chow, A., Perica, K., Klebanoff, C. A. & Wolchok, J. D. Clinical implications of T cell exhaustion for cancer immunotherapy. *Nat. Rev. Clin. Oncol.* **19**, 775–790 (2022). 36216928 10.1038/s41571-022-00689-z PMC10984554

[42] Kumagai, S. et al. The PD-1 expression balance between effector and regulatory T cells predicts the clinical efficacy of PD-1 blockade therapies. *Nat. Immunol.* **21**, 1346–1358 (2020). 32868929 10.1038/s41590-020-0769-3

[43] Gocher, A. M., Workman, C. J. & Vignali, D. A. A. Interferon-γ: teammate or opponent in the tumour microenvironment?. *Nat. Rev. Immunol.* **22**, 158–172 (2022). 34155388 10.1038/s41577-021-00566-3 PMC8688586

[44] Ivashkiv, L. B. IFNγ: signalling, epigenetics and roles in immunity, metabolism, disease and cancer immunotherapy. *Nat. Rev. Immunol.* **18**, 545–558 (2018). 29921905 10.1038/s41577-018-0029-z PMC6340644

[45] Xiao, M. et al. CD4(+) T-cell epitope-based heterologous prime-boost vaccination potentiates anti-tumor immunity and PD-1/PD-L1 immunotherapy. *J. Immunother. Cancer* **10**, e004022 (2022). 35580929 10.1136/jitc-2021-004022 PMC9114852

[46] Saleh, R. & Elkord, E. Treg-mediated acquired resistance to immune checkpoint inhibitors. *Cancer Lett.* **457**, 168–179 (2019). 31078738 10.1016/j.canlet.2019.05.003

[47] Dueñas-Gonzalez, A. et al. A phase II study of multimodality treatment for locally advanced cervical cancer: neoadjuvant carboplatin and paclitaxel followed by radical hysterectomy and adjuvant cisplatin chemoradiation. *Ann. Oncol.* **14**, 1278–1284 (2003). 12881393 10.1093/annonc/mdg333

