# Supplementary material for: Tislelizumab (anti-PD-1) plus chemotherapy as neoadjuvant therapy for patients with stage IB3/IIA2 cervical cancer (NATIC): a prospective, single-arm, phase II study
Source: Signal Transduct Target Ther. 2025 Jul 4;10:215. doi: 10.1038/s41392-025-02294-9 (PMC12227751; doi:10.1038/s41392-025-02294-9)
Supplement: Supplementary file 1 — Supplementary_Materials [file 41392_2025_2294_MOESM1_ESM.docx]

Supplementary Materials for

Tislelizumab plus chemotherapy as neoadjuvant therapy for patients with stage IB3/IIA2 cervical cancer (NATIC): a prospective, single-arm, phase II study

Jindong Sheng, Haitao Luo, Xiangyu Liu, Chunyan Liu, Wenhao Zhou, Yujie Zhao, Ruoyan Liu, Dan Li, Changxiao Xu, Bo Yang, Ying Liu, Xin Fu, Lewen Bao, Ke Wang, Jihui Hao, Wenxin Liu

Correspondence to: tjmuch_liuwenxin@126.com

**This PDF file includes:**

Materials and Methods

Supplementary Text

Figures. S1 to S5

Tables S1

Materials and Methods

**1. Inclusion and exclusion criteria**

**1.1 Inclusion criteria**

Patients participated in the study only if all the following criteria were met:

1. Female, aged 18-65 years;
2. Histopathological diagnosis of cervical squamous cell carcinoma, adenocarcinoma, or adenosquamous carcinoma;
3. Locally advanced tumors (tumor diameter ≥ 4 cm) that have not previously received any treatment refer to patients with FIGO stage IB3 and IIA2 cervical cancer (confirmed by imaging);
4. Patients must provide tumor tissue sections during the screening period; formalin-fixed paraffin-embedded tumor histopathological samples or fresh tumor histopathological samples will be sent to the central laboratory for PD-L1 expression detection;
5. ECOG performance status score of 0-1;
6. Patients’ laboratory tests meet the following requirements to confirm adequate organ and hematopoietic function: absolute neutrophil count ≥ 1.5 x 10^9^/L; hemoglobin ≥ 90 g/L (in the past two weeks without blood transfusion); platelets ≥ 90 x 10^9^/L; serum total bilirubin < 1.5 x upper limit of normal (ULN); AST and ALT ≤ 1.5 ULN; serum creatinine and urea nitrogen ≤ upper limit of normal, fasting blood glucose ≤ 126 mg/dL or ≤ 7.0 mmol/L;
7. According to the investigator, the patient should be able to follow the study procedures, restrictions, and requirements, have good compliance, and be willing to maintain contact;
8. Willing to participate in this study, female patients of childbearing age and their families must agree and sign an informed consent form.

**1.2 Exclusion criteria**

Patients were ineligible for enrollment into the study if any of the following criteria were met:

1. Patients have received chemoradiotherapy, immunotherapy, traditional Chinese medicine therapy, or surgery for cervical cancer;
2. Participation in a clinical trial with another investigational agent within 28 days prior to the first dose of the study drug; or receipt of antineoplastic therapy, including but not limited to chemotherapy, radiation therapy, or targeted therapy;
3. Previous treatment with immune checkpoint drugs;
4. Major surgical procedures (except biopsy) within 4 weeks prior to the first dose of study drug or incomplete healing of surgical incision;
5. Significant pleural effusion, pericardial effusion, or ascites requiring multiple drainages within 2 weeks prior to the first dose of the study drug;
6. Any active malignancy except basal cell carcinoma of the skin or carcinoma in situ of the breast following radical surgery;
7. History of active autoimmune diseases or autoimmune diseases that may recur; patients with the following conditions do not need to be excluded and may continue further screening: (1) Well-controlled type I diabetes; (2) Hypothyroidism (as long as hormone replacement therapy alone is used for treatment); (3) Well-controlled celiac disease; (4) Skin diseases that do not require systemic treatment (e.g., vitiligo, psoriasis, alopecia);
8. History of HIV, or severe chronic or active infection requiring systemic antibacterial, antifungal, or antiviral therapy, including tuberculosis infection. Patients with a history of active tuberculosis infection ≥ 1 year prior to screening should also be excluded unless evidence suggests appropriate treatment has been completed;
9. HBV DNA ≥ 10^4^ copies/mL at screening;
10. Previous history of interstitial lung disease, drug-induced interstitial lung disease, radiation pneumonitis, symptomatic interstitial lung disease, or any evidence of active pneumonia on chest CT scan within 4 weeks prior to the first dose of study drug;
11. Clinically significant cardiovascular disease, including but not limited to acute myocardial infarction, severe/unstable angina pectoris, cerebrovascular accident or transient ischemic attack within 6 months prior to enrollment, congestive heart failure (New York Heart Association Class III or higher heart failure; arrhythmia requiring other antiarrhythmic drugs in addition to β-blockers or digoxin; QTcF interval > 450 milliseconds (ms) by electrocardiogram repeat; hypertension not well controlled by antihypertensive drugs (systolic blood pressure > 150 mmHg, diastolic blood pressure > 100 mmHg);
12. Abnormal thyroid function is present, and thyroid function cannot be maintained in the normal range with drugs;
13. Use of immunosuppressive agents within 2 weeks prior to the first study drug treatment, excluding topical glucocorticoids or systemic glucocorticoids not exceeding 10 mg/methylprednisone or equivalent doses of other glucocorticoids;
14. Previous allogeneic stem cell transplantation or organ transplantation;
15. Live vaccines within 4 weeks (inclusive) of the first dose of study drug; (Note: Seasonally injected influenza vaccines are generally inactivated and are therefore permitted. Intranasal vaccines are live vaccines and are not allowed);
16. History of severe allergic reactions to chimeric or humanized antibodies or fusion proteins;
17. Any other condition, metabolic disorder, laboratory abnormality, or alcohol/drug abuse or dependence that could reasonably lead the investigator to suspect the patient is unsuitable for the study drug treatment, may compromise the interpretation of study results, or place the patient at high risk.

**2. Imaging assessment**

**2.1 Baseline imaging assessment**

For the baseline imaging assessment, our study utilized chest and abdominal contrast-enhanced CT scans, as well as both plain and contrast-enhanced pelvic MRI sequences. In cases where patients had metallic implants or other contraindications for MRI, contrast-enhanced CT scans of the pelvis were used as an alternative. These imaging modalities were employed to assess tumor size and determine the FIGO staging of the patients. PET/CT was not used in this study for staging or evaluation.

For the contrast-enhanced CT, iodinated contrast agents were used, while the contrast agent for MRI was gadobutrol.

For the pelvic MRI, we included both plain sequences (sagittal T2-weighted fast-recovery fast spin-echo [FRFSE] sequence; axial T2-weighted fat suppression [FS] FRFSE FS sequence; sagittal T 1-weighted fast spoiled gradient recalled [FSPGR] sequences, and sagittal DWI images) and contrast-enhanced sequences (Axial contrast-enhanced T1 weighted). Sagittal DWI images were obtained using single-shot echo-planar imaging with three b-factors (0, 800, and 1000 s/mm^2^). Contrast-enhanced MR images were obtained with a 3D spoiled gradient-pulse T1-weighted LAVA sequence before and after administration of 0.2 mmol/kg of gadolinium at a rate of 2 mL/sec, which was followed by a 30-mL saline bolus injection.

**2.2 Post-neoadjuvant imaging assessment**

In this study, post-neoadjuvant chemoimmunotherapy assessment was performed using plain and contrast-enhanced pelvic MRI sequences, to determine the tumor size and assess the radiologic response following treatment. In cases where patients had metallic implants or other contraindications for MRI, contrast-enhanced CT scans of the pelvis were used as an alternative.

The contrast agent and sequences used in the post-neoadjuvant chemoimmunotherapy assessment were the same as those used at baseline. Besides, for non-OPR patients, the residual tumor size after surgery was assessed by pathologists, who measured the maximum diameter of the tumor bed under a microscope. Microscopic examination was used to refine the tumor bed changes at the margin, excluding surrounding fibrosis, inflammatory changes, and necrotic tissue.

**3. Dose modifications**

For patients who were allergic to paclitaxel, the drug was substituted with the same dose of paclitaxel liposome. Grade ≥3 AEs required treatment interruption or delay, with active management implemented. If the AEs resolved to grade 1 or 2 within 7 days, treatment could resume without dose reduction. However, if recovery took over 7 days, the investigator had the discretion to adjust the doses of paclitaxel and carboplatin or cisplatin. Dose adjustments for tislelizumab were not permitted, but treatment could be interrupted for up to 56 days (8 weeks) if immune-related [adverse events](https://www.sciencedirect.com/topics/pharmacology-toxicology-and-pharmaceutical-science/adverse-event) (irAEs) occurred. If irAEs did not recover to grade 2 or lower within 7 days after the start of combined chemoimmunotherapy, tislelizumab treatment was discontinued. In the event of uncontrollable grade 4 AEs, the treatment would be interrupted permanently.

**4. Ovarian preservation**

In this study, for patients under 40 years old with stage IB3-IIA2 cervical cancer, ovarian preservation was considered based on patient intention. Bilateral ovarian preservation was usually performed, but if one ovary showed a mass or surface abnormality, that ovary was excised.

**5. Adjuvant therapy**

Following surgery, post-operative adjuvant therapy was determined based on the patient’s pathological response and the presence of high-risk factors identified during the postoperative pathological examination. Patients with optimum pathologic response (OPR) were recommended to undergo regular follow-ups. For patients with non-OPR, those with high-risk factors (pelvic or abdominal para-aortic lymph node-positive, margin-positive, or parametrial infiltration) were recommended to receive cisplatin-based concurrent chemoradiotherapy; with ≥2 intermediate-risk factors (deep >1/3 interstitial invasion, lymphovascular space invasion [LVSI] positive, or residual tumor size >2 cm) were given additional external beam pelvic radiotherapy; did not meet the above criteria underwent 3 cycles of adjuvant chemotherapy postoperatively.

**6. Exploratory analysis**

***Next-generation sequencing and data analysis***

YuceOnePro includes 1012 genes, which are mainly involved in tumor and immune-related genes. In this research, we used this panel to sequence tumor samples from 20 patients before and after neoadjuvant therapy. Tissue DNA was implemented on Formalin-fixed paraffin-embedded (FFPE) tumor biopsies and matched peripheral blood samples. Blood samples were collected into cell-free DNA blood streck tubes. Isolated plasma was stored at -80℃ until extraction. Cell-free DNA (cfDNA) was extracted from 4 mL plasma using QIAamp Circulating Nucleic Acid Kit (Qiagen, Hilden, Germany) according to the manufacturer’s instructions. GeneRead DNA Kit (QIAGEN, GER) was employed for tumor tissue extraction, while Mag-Bind® Blood & Tissue DNA HDQ 96 kit (OMEGA) was utilized for blood sample extraction. The dsDNA HS Assay Kit (ThermoFisher Scientific, USA) was used for DNA quantification. Sequencing libraries were built by Exome Plus Panel V1.0 (IDT, USA), and sequencing procedures were utilized by the MGISEQ platform with 100-bp paired-end reads. SOAPnuke was implemented to cut adapters and remove low-quality raw reads. Clean reads were aligned against the human reference genome (hg19) with BWA (v0.7.12), and duplicated reads were removed by Sambamba (v0.5.4). Subsequently, generated BAM files were used for downstream analysis.

***Somatic variant calling***

We compared tumor and matched blood sequencing data to identify the somatic mutations, including single nucleotide variants (SNVs) and small insertions and deletions (Indels), by mutation caller Vardict (v1.7.0) with default parameters. Three callers were run with dbSNP (version 147), 1000 G (phase3_release_v5), CLINVAR (version 151) and COSMIC (version 81) data for known polymorphic sites. Substitutions and indels with low variant allelic fractions (VAF < 0.02) or low read coverages were filtered out. Mutations called by at least 2 callers were retained. In addition, the filtered mutations were annotated by snpEff (v4.3) with NCBIrefseq (https://www.ncbi.nlm.nih.gov/refseq/).

***Tumor mutational burden and intratumoral heterogeneity analysis***

TMB was defined as the number of nonsynonymous somatic mutations per megabase. Secondly, microsatellite instability (MSI) was called by MSIsensor (v0.6). Ascatngs (v3.1.0) was implemented on the read alignments from targeted sequencing data to identify copy number variants, by correcting for GC content, and tumor purity and comparing to the matched blood sample. Tumor purity and ploidy were measured by ascites as well. PyClone (v0.13.1) was used to estimate the number of clones and calculate the cellular prevalence of inferred mutational clusters. Intratumoral heterogeneity (ITH) was defined as the proportion of the number of subclonal mutations to the total number of mutations (sum of clonal mutation number and subclonal mutation number).

***Whole transcriptome sequencing***

The total RNA of tumor samples was isolated using RNeasy Plus Universal Kits (Qiagen, GER). RNA concentration was quantified using QubitTM RNA HS Assay Kit (ThermoFisher Scientific, USA). RNA purity and integrity were analyzed using Take3 (BioTek, USA) and the RNA Cartridge kit of the Qseq100 Bio-Fragment Analyzer (Bioptic, CHN), respectively. Then, RNA sequencing (RNA-seq) libraries were constructed using the rRNA depletion module (H/M/R) and NadPrep DNA Library Preparation Module for MGI (MGI, CHN). Libraries were sequenced on the MGISEQ platform with 100 bp paired-end reads.

***RNA-Seq raw data quality control and gene expression analysis***

Raw RNA sequencing data from the sequencer were processed to filter out low-quality reads. Clean reads from each sample were obtained and used for the following analysis. The RNA reads were aligned against reference hg19 and gencodev27lift37 database (download from https://www.gencodegenes.org) by STAR (v2.7.8a). Based on the aligned reads, raw count and transcripts per million (TPM) values were calculated in Rsem (v1.3.0). Then, gene expression levels were summarized from transcript levels. Differentially expressed genes (DEGs) were identified by the DESeq2 package. The genes with fold changes >2 or < -2 and P-value <0.01 were considered as DEGs. Volcano plots and heatmaps were drawn in R with ggpubr and Complexheatmap package. Gene Ontology (GO) and Kyoto Encyclopedia of Genes and Genomes (KEGG) pathway enrichment were analyzed by the KOBAS-i webtool KOBAS.

***Immune signature and infiltration abundance of immune cells***

Based on the gene expression matrix, a series of immune signatures were implemented to assess tumor immune signatures comprised of cytolytic, IFN-gamma, T-cell, Batf3-DC, and HLA. R packages xCell were used to estimate the infiltration abundance of immune cells for each sample. Immune-related signature estimation was employed to evaluate the signatures comprised of cytolytic, IFN-gamma, T-cell, Batf3-DC, and HLA.

***PD-L1 IHC***

PD-L1 IHC was performed using the PD-L1 IHC 22C3 pharmDx kit (Dako) on the Dako ASL48 platform according to manufacturer recommendations. HER2 IHC was performed using the HER2/neu kit (Ventana) following the standard preprogrammed staining protocol. The anti-PD-L1 antibody (clone: 22C3) and anti-HER2 antibody (clone: 4B5) were provided already diluted at an unspecified ratio in the kit.

Supplementary Text


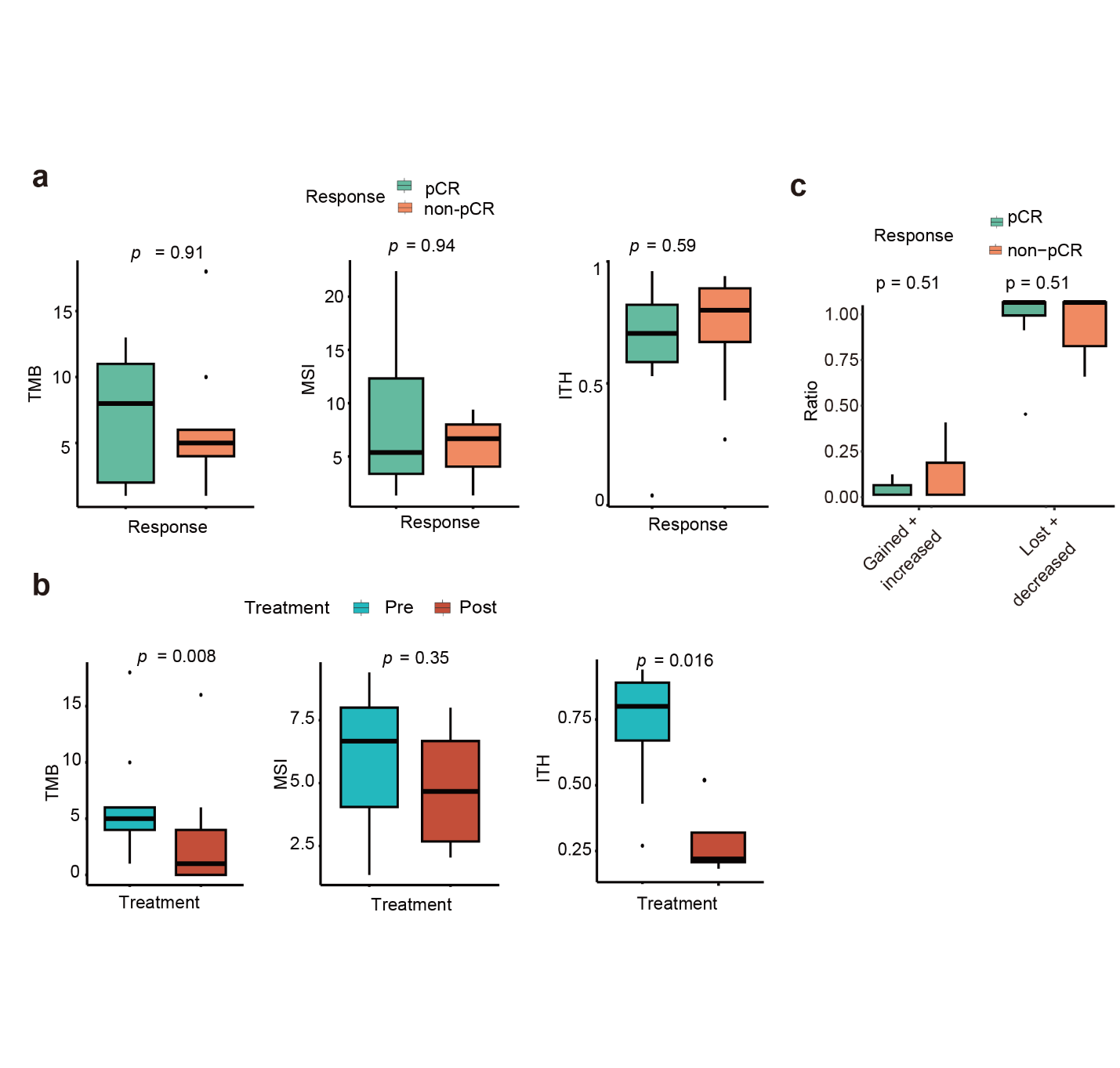
Figure. S1. A comprehensive comparison of tissue-based TMB, MSI, and ITH between pCR and non-pCR groups at baseline and changes in these parameters post-treatment.

A. Differences in baseline tumor mutation burden (TMB), microsatellite instability (MSI), and intratumor heterogeneity (ITH) between the pCR and non-pCR groups. B. Changes in TMB, MSI, and ITH levels before and after treatment in the non-pCR group. C. During treatment, the correlation between changes in gene mutations and pathological response. TMB, tumor mutation burden; MSI, microsatellite instability; ITH, intratumor heterogeneity; pCR, pathological complete response.


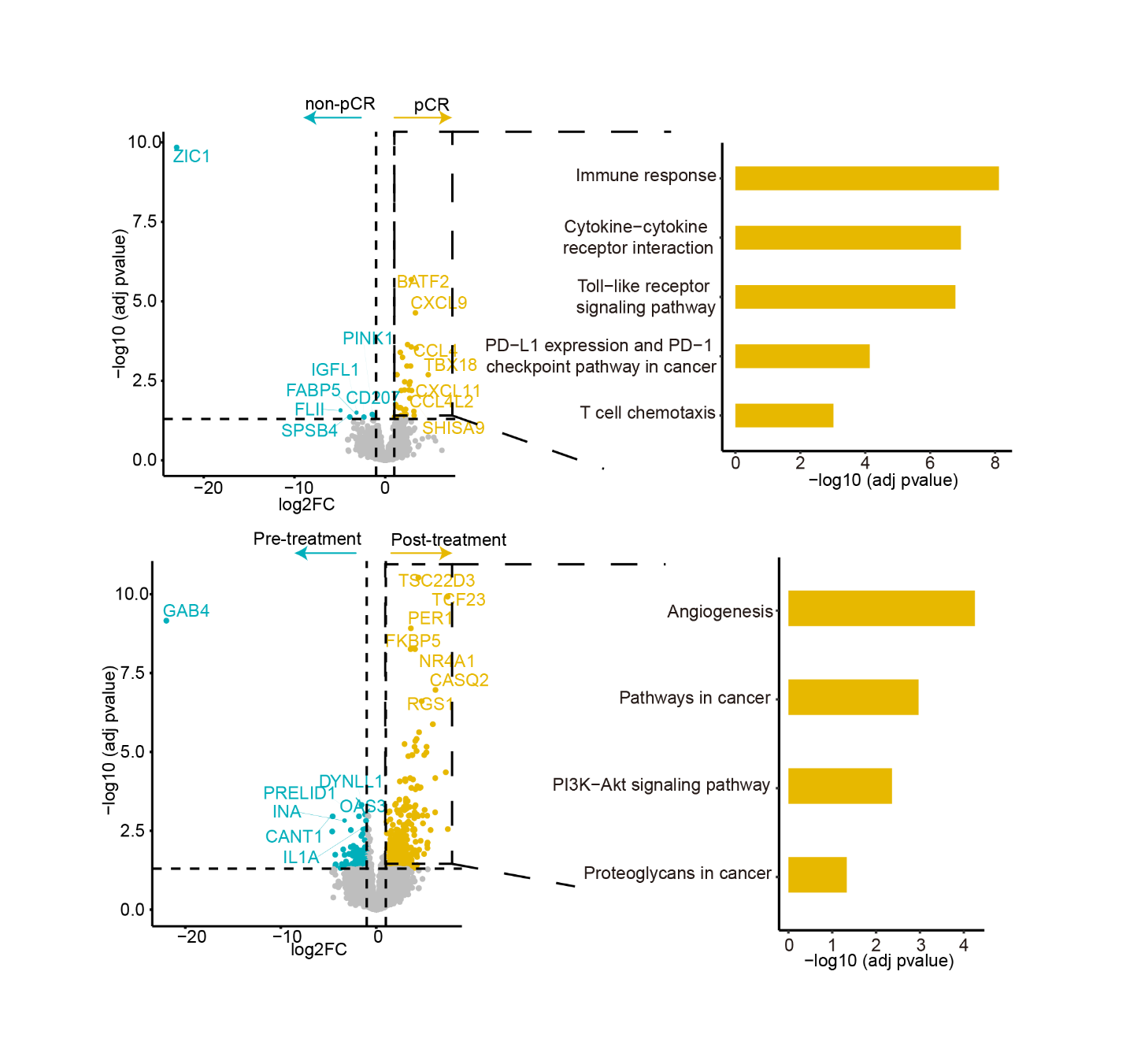
Figure S2. The results of differential gene expression analysis and pathway enrichment analysis after correction for multiple hypothesis testing.

In the baseline comparison, key genes like BATF2, CXCL19, and CD274 remained significant even after multiple hypothesis testing corrections. The enriched pathways were consistent with initial findings. Additionally, when examining non-pCR patients before and after treatment, the corrected differential gene expression analysis revealed minimal changes, with subsequent pathway enrichment results remaining largely unchanged, indicating robustness in the data and analysis. pCR, pathological complete response.


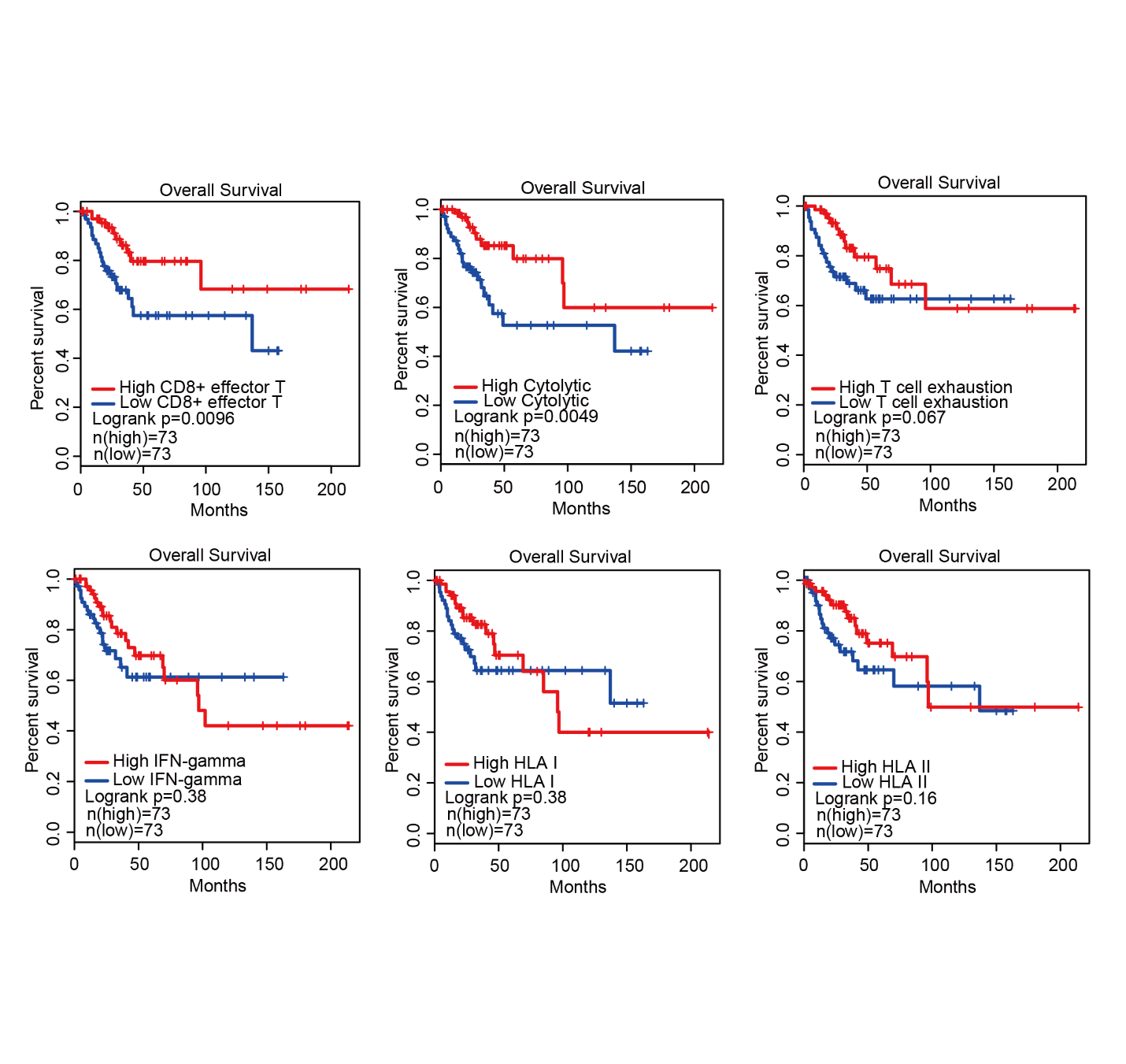
Figure S3. The impact of baseline immune-related signatures on OS was validated using the external TCGA-CESC cohort

Our findings indicated that high levels of CD8+ effector T cells and cytolytic activity signatures significantly prolong OS. In contrast, signatures related to HLA class I, HLA class II, IFN-γ, and T-cell exhaustion did not significantly extend OS. OS, overall survival.


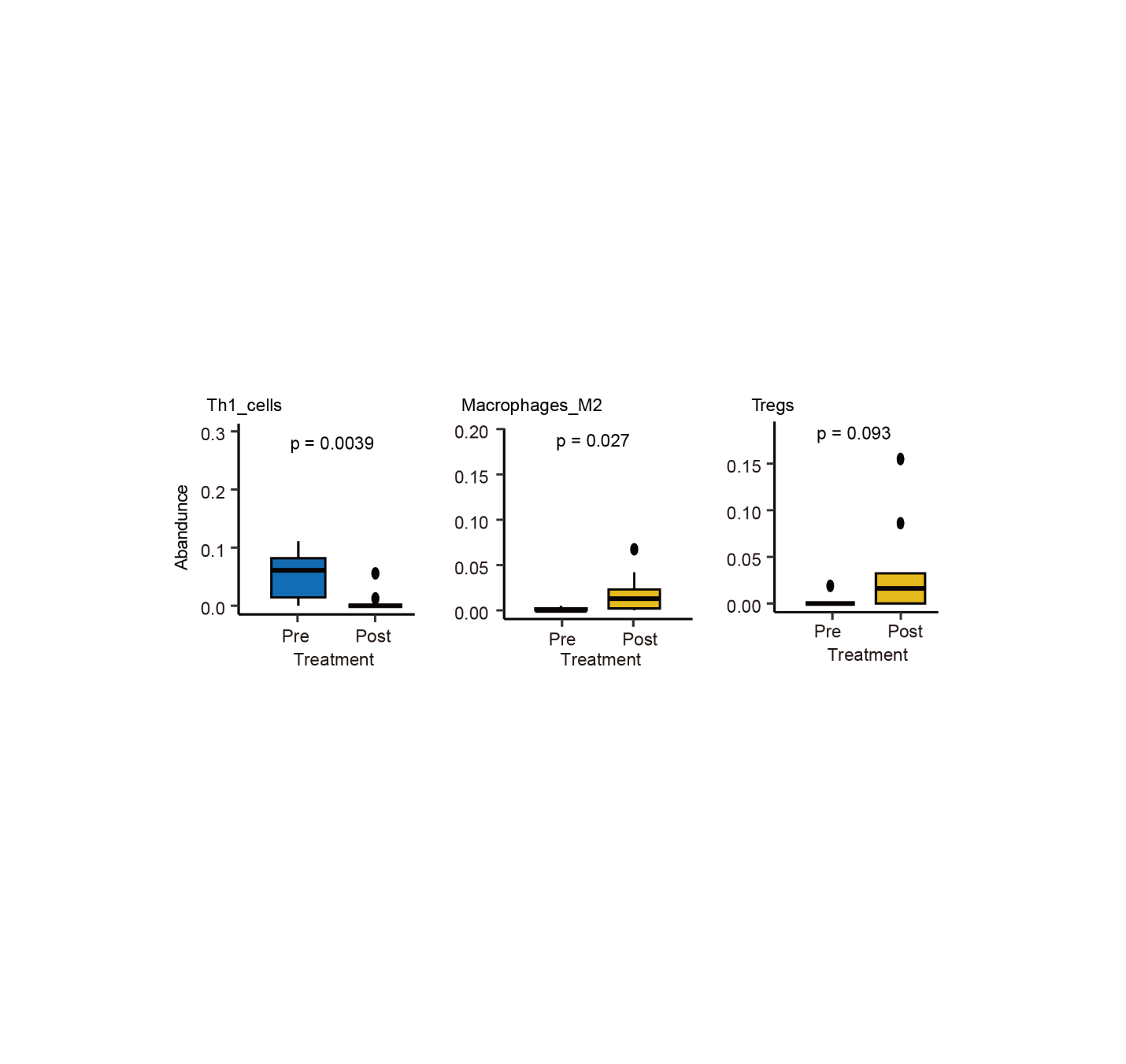
Figure S4. Changes in immune cells in the non-pathological complete response group during neoadjuvant therapy.


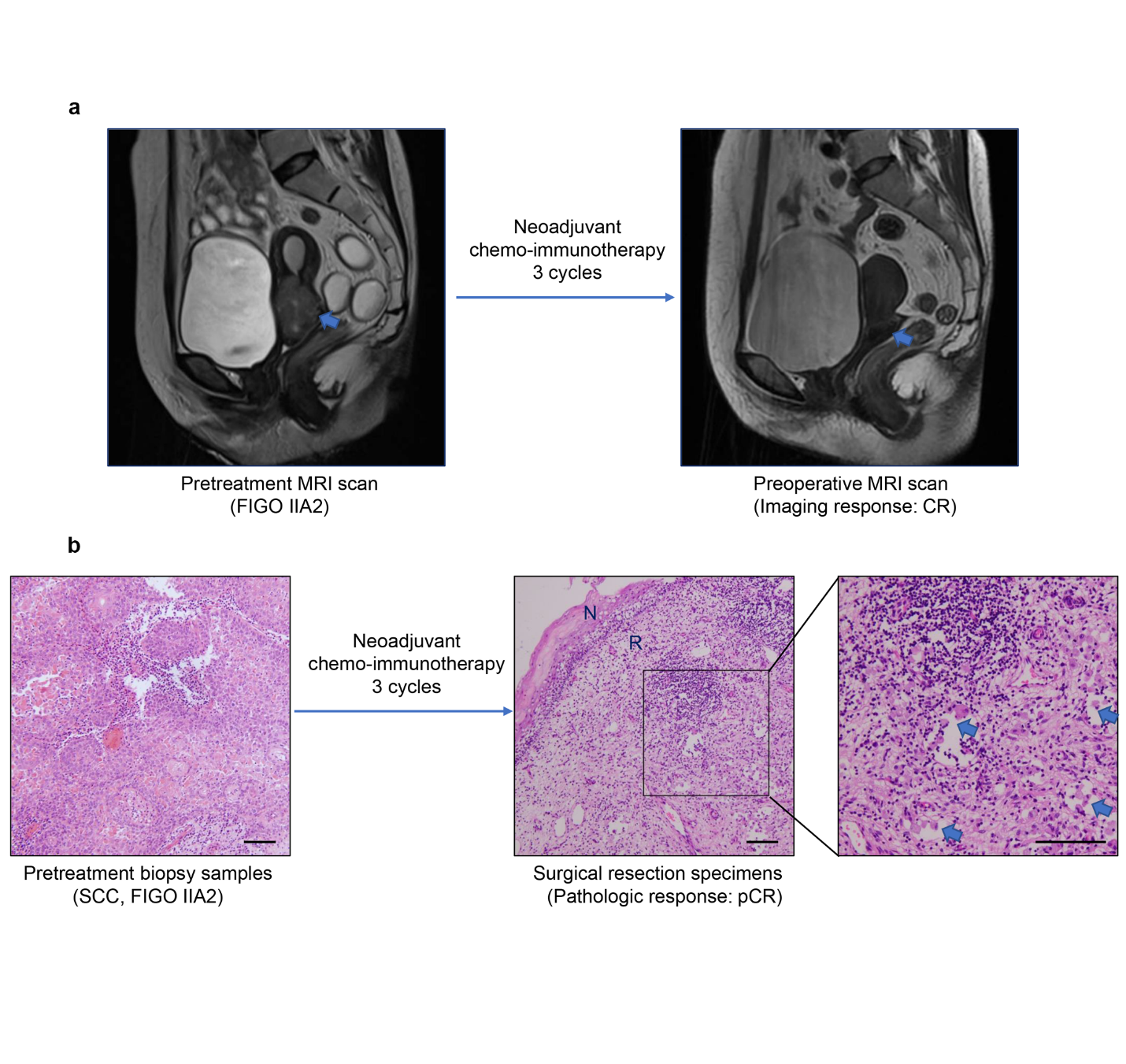


Figure S5. Neoadjuvant chemo-immunotherapy response in patients with locally advanced cervical cancer

A. Change in tumor size after neoadjuvant chemotherapy through magnetic resonance imaging. B. Representative sections of the radical surgery resected specimen before and after neoadjuvant therapy (HE staining, 200×). Scale bar=20μm. pCR, pathological complete response; FIGO, International Federation of Gynecology and Obstetrics; SCC, squamous cell carcinoma.

Table S1. Reasons for delayed surgery

| Patient ID | Pathologic response | Time to surgery, days | Reasons for delayed surgery |
| --- | --- | --- | --- |
| 2 | pCR | 68 | the COVID-19 pandemic and the Spring Festival holiday |
| 4 | non-OPR | 44 | personal financial reasons |
| 19 | pCR | 49 | adverse events: neutropenia, nausea, vomit, and pain |
| 24 | pCR | 91 | adverse event: hypothyroidism |
| 27 | pCR | 61 | adverse events: hyperglycemia, deep venous thrombosis, and hepatic dysfunction |
| 28 | pCR | 57 | adverse event: hypothyroidism |
