# Supplementary material for: Tislelizumab (anti-PD-1) plus chemotherapy as neoadjuvant therapy for patients with stage IB3/IIA2 cervical cancer (NATIC): a prospective, single-arm, phase II study
Source: Signal Transduct Target Ther. 2025 Jul 4;10:215. doi: 10.1038/s41392-025-02294-9 (PMC12227751; doi:10.1038/s41392-025-02294-9)
Supplement: Supplementary file 2 — Protocol [file 41392_2025_2294_MOESM2_ESM.docx]

**Study Title: Tislelizumab plus chemotherapy as neoadjuvant therapy for patients with stage IB3/IIA2 cervical cancer (NATIC): A prospective, single-arm, phase II study**

**CLINICAL STUDY PROTOCAL**

**Version 1.0**

**Research Center: Tianjin Cancer Hospital**

**Principal Investigator: Wenxin Liu**

**Clinical study registration number: ChiCTR2200065392**

Contents

[**1. Research background** 1](#_Toc190869151)

[1.1 Epidemiology 1](#_Toc190869152)

[1.2 Treatment status 1](#_Toc190869153)

[1.3 Immunotherapy 1](#_Toc190869154)

[1.4 Tislelizumab 3](#_Toc190869155)

[**2. Study design** 5](#_Toc190869156)

[2.1 Study overview 5](#_Toc190869157)

[2.2. Study flowchart 5](#_Toc190869158)

[2.3 Treatment options 5](#_Toc190869159)

[2.3.1 Tislelizumab injection 5](#_Toc190869160)

[2.3.2 Platinum-based chemotherapy 6](#_Toc190869161)

[2.3.3 Surgery 6](#_Toc190869162)

[2.4 Assessment of therapeutic effects 6](#_Toc190869163)

[2.4.1 Therapeutic effects criteria 6](#_Toc190869164)

[2.4.2 Therapeutic efficacy evaluation index 6](#_Toc190869165)

[2.5 Stratification factors 6](#_Toc190869166)

[**3. Study population** 6](#_Toc190869167)

[3.1 Inclusion criteria 6](#_Toc190869168)

[3.2 Exclusion criteria 7](#_Toc190869169)

[3.3 Rejection criteria 8](#_Toc190869170)

[3.4 Trial exit criteria 8](#_Toc190869171)

[**4. Investigational medicinal product** 9](#_Toc190869172)

[4.1 Introduction to tislelizumab 9](#_Toc190869173)

[4.1.1 Pharmacology 9](#_Toc190869174)

[4.1.2 Absorption, distribution, metabolism and excretion 9](#_Toc190869175)

[4.1.3 toxicology 10](#_Toc190869176)

[4.1.4 Clinical data 10](#_Toc190869177)

[4.2 Instructions for using investigational medicinal product 11](#_Toc190869178)

[4.2.1 PD-1 antibody 11](#_Toc190869179)

[4.2.2 Chemotherapy drugs 11](#_Toc190869180)

[4.2.3 Drug dose delay or adjustment 12](#_Toc190869181)

[4.3 General guidance for dose adjustment 12](#_Toc190869182)

[4.3.1. Dose suspension or adjustment of tislelizumab 13](#_Toc190869183)

[4.3.2 Suspension, interruption or adjustment of chemotherapy dose 13](#_Toc190869184)

[4.3.3. Criteria for stopping chemotherapy regimen 15](#_Toc190869185)

[4.4. Concomitant medication and restricted medication 16](#_Toc190869186)

[4.4.1. Concomitant medication 16](#_Toc190869187)

[4.4.2 Restricted medication 16](#_Toc190869188)

[**5. Study procedure** 16](#_Toc190869189)

[**6. Biomarkers and tumor tissue samples** 17](#_Toc190869190)

[**7. Description of study endpoints** 17](#_Toc190869191)

[7.1 Primary study endpoints 17](#_Toc190869192)

[7.2 Secondary study endpoints 17](#_Toc190869193)

[7.3 Exploratory study endpoints 18](#_Toc190869194)

[**8. Detailed evaluation criteria** 18](#_Toc190869195)

[8.1 Tumor and efficacy assessment 18](#_Toc190869196)

[8.2. Safety assessment 19](#_Toc190869197)

[8.2.1 Vital signs 19](#_Toc190869198)

[8.2.2 Physical examination 19](#_Toc190869199)

[8.2.3 Eastern Cooperative Oncology Group (ECOG) Performance Status 19](#_Toc190869200)

[8.2.4 Laboratory safety inspection 19](#_Toc190869201)

[8.3. Adverse Events 21](#_Toc190869202)

[8.3.1 Definition of adverse events 21](#_Toc190869203)

[8.3.2 Severity assessment 21](#_Toc190869204)

[8.3.3 Causality assessment 22](#_Toc190869205)

[8.3.4 Definition of serious adverse events 22](#_Toc190869206)

[8.3.5 Management of adverse events of special concern 24](#_Toc190869207)

[**9. Testing and statistical items** 25](#_Toc190869208)

[9.1 Screening period 25](#_Toc190869209)

[9.1.1 Test items to be completed within 2 weeks before enrollment 25](#_Toc190869210)

[9.1.2 Test items to be completed within 1 week before enrollment 26](#_Toc190869211)

[9.2 Treatment period 26](#_Toc190869212)

[9.2.1 Clinical observation indicators during medication 26](#_Toc190869213)

[9.2.2 Follow-up period 26](#_Toc190869214)

[**10. Statistical considerations** 27](#_Toc190869215)

[10.1 Analysis set/analysis population 27](#_Toc190869216)

[10.2 Efficacy analysis 28](#_Toc190869217)

[10.3 Safety analysis 28](#_Toc190869218)

[10.4 Sample size 28](#_Toc190869219)

[References 52](#_Toc190869220)

# **1. Research background**

## 1.1 Epidemiology

Cervical cancer ranks as the fourth most common malignant tumor in terms of incidence and mortality worldwide, following breast cancer, colorectal cancer, and lung cancer. According to a 2008 report by the World Health Organization (WHO), there are approximately 529,800 new cases globally each year, with 85.6% of these cases (453,300) occurring in developing countries [1]. The latest cancer data in China indicates that in 2016, cervical cancer ranked ninth in the top ten cancer incidence rates in registered areas (with 119,000 new cases and 37,000 deaths), holding the top position among female reproductive tract tumors. Both the incidence and mortality rates for cervical cancer are on the rise [2]. Pathological types include squamous cell carcinoma, adenocarcinoma, adenosquamous carcinoma, and rare types such as small cell carcinoma, clear cell carcinoma, and sarcoma with low incidence rates.

## 1.2 Treatment status

Before stage ⅡB, surgery was the main treatment method. Locally advanced cervical cancer (LACC) refers to patients with a tumor diameter ≥4 cm and staged between 1B3 and 2A2 according to the 2018 International Federation of Gynecology and Obstetrics (FIGO) staging system. The difficulty of surgical resection is higher in these cases, making it hard to ensure the scope of parametrial resection which can lead to complications such as ureteral injury, affecting both short-term and long-term surgical outcomes. Previous studies have shown that using neoadjuvant chemotherapy (NACT) for LACC can shrink tumors, eliminate micro-metastases in lymphatic vessels, improve local control rates, and increase the rate of successful surgical resection. Literature has confirmed that the objective response rate of NACT for LACC is between 67%-90%, with an effective pathological response rate of 21.3%-48.3%. The rate of achieving a complete pathological response (CR) post-surgery range from 9%-18%, with manageable toxic side effects. NACT can increase the rate of successful surgical resection to 48%-100% without additional surgical complications. Patients with large tumors or adenocarcinoma show lower response rates to NACT. Patients who respond to treatment have lower rates of metastasis and lower stromal depth compared to non-responders. There is no statistical difference in progression-free survival (PFS) and overall survival (OS) between the NACT + surgery group and the surgery-alone group [3-4].

The 2021 guidelines for diagnosis and treatment pointed out that neoadjuvant therapy is mainly used for locally advanced cervical cancer IB3 or IIA2 stage, usually for 2-3 courses, to improve local control rate and surgical clearance rate. The 2022 NCCN guidelines also suggest that for patients with stage IB-IIA, neoadjuvant chemotherapy (NACT) can effectively reduce tumor size and metastasis[5]. Currently, it is common in clinical practice to administer 2-3 courses of paclitaxel combined with platinum-based neoadjuvant chemotherapy followed by surgical treatment for patients with locally advanced cervical cancer.

## 1.3 Immunotherapy

The immune system plays a crucial role in detecting and clearing abnormal cells in the human body. Tumor cells employ various strategies to evade immune surveillance, one of which is immune checkpoint inhibition. programmed cell death-1 (PD-1) is an important immune checkpoint inhibitor. The PD-1 receptor is a programmed cell death protein and a member of the T cell receptor CD28 family. It is a negative co-stimulatory molecule expressed on activated CD4 and CD8 T cells, B cells, monocytes, natural killer cells, and dendritic cells. In normal conditions, PD-1 mainly functions as an immune checkpoint, exerting negative control on T cell activity by binding to its ligands. The key ligands of PD-1 include programmed cell death ligand-1 (PD-L1) and Programmed cell death 1 ligand 2 (PD-L2). PD-L1 is expressed on T and B cells, dendritic cells, macrophages, mesenchymal stem cells, and other non-hematopoietic cells. PD-L1/2, when bound to PD-1 expressed on lymphocytes in the tumor microenvironment, mainly produce inhibitory tyrosine phosphatases, inhibit downstream PI3K/AKT signaling, cause cell cycle arrest and T cell activation suppression, hinder the anti-tumor immune response, and assist tumor immune evasion. Thus, PD-1/PD-L has become a rational target for tumor immunotherapy to unleash the inherent anti-tumor effects of the immune system [6]. The expression of PD-L1 and PD-L2 has been confirmed in various solid tumors, including non-small cell lung cancer, gastric cancer, liver cancer, renal cancer, bladder cancer, breast cancer, lymphoma, leukemia, and others. Among various tumor types, PD-L1 CPS≥1 positivity rates rank highest. Retrospective studies have confirmed a PD-L1 positivity rate of approximately 60%-91% [7-10], indicating that these tumors should respond well to immunotherapeutic agents.

There have been significant advances in the field of gynecologic oncology in the past three years, particularly in the treatment of endometrial cancer with immunotherapy checkpoint inhibitors. In 2019, the results of the KEYNOTE-158 trial were published. The study included 98 patients with recurrent or metastatic disease who received pembrolizumab monotherapy. The objective response rate (ORR) for PD-L1 positive patients was 14.6%, with a median PFS of 2.1 months and a median OS of 11 months, while no PD-L1 negative patients showed any response [11]. Based on this study, pembrolizumab received accelerated FDA approval for a second-line treatment in advanced PD-L1-positive cases, becoming the first approved immunotherapy drug and being included in the NCCN guidelines.

The EMPOWER-CERVICAL1 study presented by ESMO in 2021 enrolled patients without consideration of PD-L1 expression. The study compared the PD-1 inhibitor cemiplimab with monotherapy chemotherapy for the treatment of platinum-containing chemotherapy-refractory/relapsed advanced cervical cancer. It showed that cemiplimab extended median overall survival by 3.5 months in the overall population compared to chemotherapy, with a 31% reduction in the risk of death (12.0 months vs 8.5 months, HR=0.69, P=0.0001). The ORR increased by 10.1% (16.4% vs 6.3%), and the median duration of response (DOR) was prolonged by 9.5 months (16.4 months vs 6.9 months). The survival outcomes for both squamous cell carcinoma and adenocarcinoma populations showed significant extensions in overall survival compared to chemotherapy [12].

The results of the landmark study KEYNOTE-826, which compared immunotherapy combined with chemotherapy ± anti-vascular targeted therapy with chemotherapy ± anti-vascular generation targeted therapy, were also announced at the 2021 ESMO Congress.

Compared with a placebo, the addition of pembrolizumab plus paclitaxel plus cisplatin/carboplatin ± bevacizumab significantly improved the PFS and OS of patients with advanced relapsed disease, regardless of the overall population, patients with PD-L1 CPS≥1, or CPS≥10. The addition of pembrolizumab significantly increased the ORR and prolonged DOR in patients. For women with recurrent or metastatic disease, the addition of pembrolizumab to chemotherapy ± bevacizumab can significantly improve the prognosis of patients [13].

Currently, in the 2022 NCCN guidelines, immunotherapy is recommended for first-line or second-line treatment of recurrent cancer. However, there is no research data available on the addition of immunotherapy to neoadjuvant therapy, both domestically and internationally. Nonetheless, in other solid tumors, immunotherapy neoadjuvant treatment has shown remarkable results. For example, in breast cancer, immunotherapy neoadjuvant treatment has significantly improved patient outcomes. In 2019, the first Phase III study data on immunotherapy neoadjuvant treatment for breast cancer was released by ESMO, showing that the addition of pembrolizumab to neoadjuvant chemotherapy increased the pathological response rate from 51.2% to 62.8% compared to the placebo control group [14]. Additionally, a mid-term analysis of the KEYSTONE-001 single-center, single-arm Phase II clinical trial led by Professor Jiang Hongjing, Director of Minimally Invasive Surgery for Esophageal Tumors at Tianjin Medical University Cancer Hospital, showed that the postoperative pathological response rate reached 72.4% (21/29) when pembrolizumab was combined with paclitaxel and cisplatin, including 12 cases of pathological complete response (ypT0N0, 41.1%) and 17 cases reaching ypT0 (58.6%). The treatment was well-tolerated with no Grade 3 or higher immune-related adverse reactions [15]. These studies indicate that combining immunotherapy with neoadjuvant chemotherapy can significantly increase the surgical resection rate for locally advanced tumors. For immunogenic tumors, the trend of moving towards immunotherapy agents earlier in treatment may potentially change the future of therapy.

Ongoing clinical studies investigate immune checkpoint inhibitors as adjuvant therapy for advanced localized tumors, with PD-1 combined with chemotherapy being the treatment regimen. These trials, as detailed in Table 1[16], are all in phases 1-2 and focus on endpoints such as pathologic response rate (pCR), ORR, Disease-free survival (DFS), and OS. The selected immune checkpoint inhibitors include pembrolizumab, sintilimab, and camrelizumab.

Table 1. Summary of clinical studies on immunotherapy in adjuvant chemotherapy

| **Type of study** | **Indications** | **plan** | **Primary End Point** | **Secondary End Points** | **Number of participants** | **nation** | **Enrollment Center** | **Start time** | **Completion time of primary endpoint** |
| --- | --- | --- | --- | --- | --- | --- | --- | --- | --- |
| Ph2, one arm | 1B3/ 2A2, tumor > 3.5 cm | Taxane + cisplatin + Sindily * 3 cycles | pCR | ORR | 47 | China | Sun Yat-sen Memorial Hospital of Sun Yat-sen University -Jihong Liu | 2021.3 | 2023.3 |
| Ph2, one arm | 1B3, 2A2, 2B (tumor > 4 cm); SC, ADS, AD; PD-L1+ | Cisplatin + white purple * 1 - Cisplatin + white purple + carrelizumab * 2 cycles | ORR | CR, DFS, ORR, OS, relapse | 84 | China | Tongji Hospital of Huazhong University of Science and Technology-Ding Ma | 2020.12.1 | Expected 2022.12.1 |
| Ph2, one arm | 1B2-2B; AD, ADS, SCC; PDL1+>1% | Carboplatin + Taxane + K drug * 3 cycles - Surgery - Carboplatin + Taxane + K drug * 3 cycles - K drug maintenance (until PD/toxicity/up to 35 cycles) | 2y PFS | AE, CR, ORR,  OS, quality of life | 45 | Italy | 13 | 2021.2.18 | Expected 2023.9.1 |

## 1.4 Tislelizumab

Tislelizumab is currently the only successfully engineered PD-1 monoclonal antibody with special modifications to the Fc segment. Through Fc modification, it avoids antibody-dependent cell-mediated phagocytosis (ADCP effect) and prevents depletion of effector T cells; at the same time, tislelizumab has unique binding sites and a longer affinity. In clinical studies across various tumor types, tislelizumab has demonstrated very good efficacy and safety. Currently, tislelizumab has been approved for indications in Hodgkin's lymphoma, urothelial carcinoma, squamous non-small cell lung cancer, and other tumors. Its use is recommended in multiple authoritative domestic guidelines.

In the field of gynecologic oncology, in 2022, SGO reported the latest results of the toripalimab RATIONALE 209 study. This is a phase II single-arm study enrolling patients with previously treated unresectable locally advanced or metastatic MSI-H/dMMR solid tumors, who received toripalimab monotherapy until intolerable toxicity or disease progression. The study ultimately included 80 patients, with an ORR of 45.9%. Among the cohort, 17 cases of gynecologic tumors were included, with an ORR of 53.3% and a CR rate of 20%. Among the 13 cases of endometrial cancer, the ORR was 46.2%, with 1 case achieving partial response (PR) and 1 case achieving PR. The median follow-up was 17.5 months, and the median DOR, PFS, and OS have not yet been reached. The study also demonstrated that toripalimab monotherapy had good safety and tolerability. Based on this study, toripalimab was approved for the treatment of unresectable or metastatic microsatellite high instability (MSI-H) or mismatch repair deficient (dMMR) adult solid tumors in China in March 2022. [15-21]

Based on the good response of immune-related drugs to late-stage recurrence in the past and the favorable safety data of toripalimab, we are applying to conduct this clinical trial to provide LACC patients with PD-1 antibody toripalimab + paclitaxel/platinum regimen neoadjuvant chemotherapy combined with surgical treatment, to evaluate whether it can improve the effective pathological remission rate, objective remission rate of LACC patients, and the safety of the combined treatment regimen. Similar studies have not been conducted domestically or internationally yet, and it is expected that good trial results can be obtained, enhancing the efficacy of neoadjuvant chemotherapy, especially the NACT effect on large, localized tumors and adenocarcinoma, improving the local control rate and surgical margin-negative rate, and improving the short-term and long-term efficacy of high-risk patients, which will have good socio-economic effects.

The research group plans to further explore the changes in the tumor microenvironment induced by immunotherapeutic agents by comparing tissue samples before and after immunotherapy with molecular biology techniques and bioinformatics methods based on the current clinical study. This deeper basic research will be conducted to apply for a new research project.

# **2. Study design**

## 2.1 Study overview

This study is a single-center prospective single-arm phase II clinical trial, with the primary endpoint being the complete pCR of locally advanced patients treated with Tislelizumab + paclitaxel/platinum-based neoadjuvant chemotherapy combined with surgery. Secondary endpoints of the study include ORR, DFS, OS, and safety. Exploratory endpoints included tissue-based and blood-based biomarkers to uncover the biological drivers behind the clinical outcomes.

The plan is to enroll a total of 30 participants. Based on literature data combined with clinical experience, the pathological response rate after neoadjuvant treatment with paclitaxel plus cisplatin in locally advanced patients is approximately 20%. It is expected that the pathological complete response rate can be increased to 45% when combined with PD-1 antibodies. Using Simon's two-stage design with one-sided α=0.025 and β=0.2, the required sample size is 30 cases. The intention is to enroll 10 patients in the first stage, and if 3 patients achieve the pathological response rate, the study will proceed to the second stage, enrolling an additional 20 patients, for a total of 30 patients in the study.

## 2.2. Study flowchart


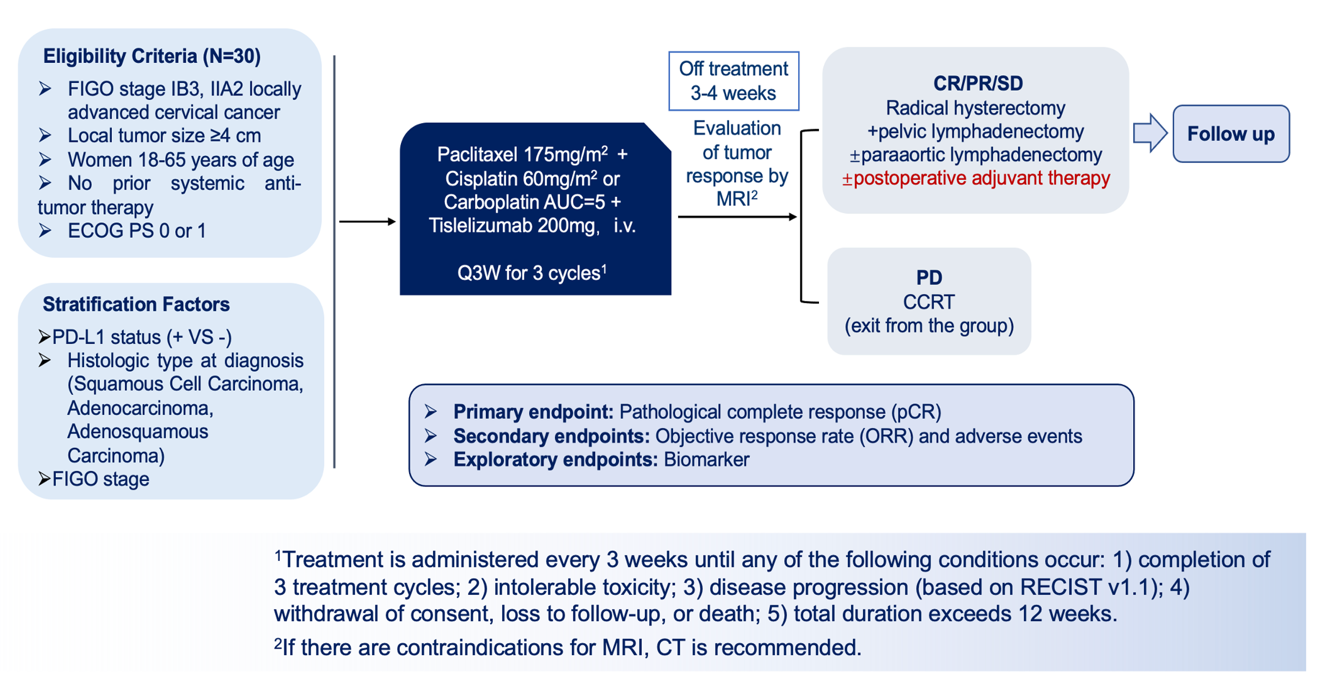


FIGO, International Federation of Gynecology and Obstetrics; ECOG, Eastern Cooperative Oncology Group; CR, Complete Response; PR, Partial Response; SD, Stable Disease; MRI, Magnetic Resonance Imaging; PD, Progressive Disease; CCRT, Concurrent Chemoradiotherapy; pCR, Pathological Complete Response; ORR, Objective Response Rate; AUC, Area Under the Curve; i.v, Intravenous; CT, Computed Tomography; PD-L1, Programmed Death-Ligand 1; RECIST, Response Evaluation Criteria in Solid Tumors.

## 2.3 Treatment options

### 2.3.1 Tislelizumab injection

Drug Specification: 100 mg per vial.

Administration and Dosage: Tislelizumab antibody 200 mg, intravenous infusion (not less than 60 minutes for the first cycle, not less than 30 minutes for subsequent cycles), once every 3 weeks, for a total of 3 cycles.

### 2.3.2 Platinum-based chemotherapy

Paclitaxel 175mg/m^2^ + Carboplatin AUC = 5/ Cisplatin 60 mg/m^2^, administered once every 3 weeks for a total of 3 cycles.

### 2.3.3 Surgery

Radical surgery (total hysterectomy + bilateral/unilateral adnexectomy + pelvic lymph node dissection + para-aortic lymph node dissection), performed by the attending physician in the gynecologic oncology department.

## 2.4 Assessment of therapeutic effects

### 2.4.1 Therapeutic effects criteria

Response Evaluation Criteria in Solid Tumors (RECIST 1.1) is used for assessing the efficacy of solid tumor treatment. See Appendix 6 for details.

### 2.4.2 Therapeutic efficacy evaluation index

1) pCR, absence of any residual viable tumor in the primary tumor and lymph nodes

2) MPR (major pathological response), defined as ≤10% residual viable tumor and ≤3 mm cervical stromal invasion in depth.

3) ORR

Researchers will use RECIST 1.1 to evaluate the objective response rate of tumors. Detailed information on target lesions or non-target lesions will be collected in the Case Report Form (CRF) to calculate tumor objective response. Tumor assessments post-baseline should use the same evaluation methods (CT, MRI) as baseline and preferably be conducted at the same hospital.

The objective response rate of tumors is calculated based on the percentage of evaluable subjects with CR and PR.

## 2.5 Stratification factors

1) PD-L1 expression level

Negative (CPS<1), low expression (1≤CPS＜10), or high expression (CPS≥10).

2) Pathological types: Squamous cell carcinoma, Adenocarcinoma, Adenosquamous carcinoma

# **3. Study population**

## 3.1 Inclusion criteria

1) Female, aged 18-65 years old.

2) The pathology diagnosis is squamous cell carcinoma, adenocarcinoma, or adenosquamous carcinoma.

3) Patients with locally advanced tumors (tumor diameter ≥ 4 cm) at FIGO stage IB3 or IIA2, who have not received any prior treatment, as confirmed by imaging.

4) Patients in the screening period must provide tumor tissue slices; fixed paraffin-embedded tumor histopathology specimens or fresh tumor histopathology specimens are sent to the central laboratory for PD-L1 expression testing.

5) ECOG physical fitness score 0-1 point [more information in appendix 2]

6) The patient's laboratory tests must meet the following requirements to confirm adequate organ and hematopoietic system function: absolute neutrophil count ≥1.5×10^9^/L; hemoglobin ≥90g/L (without receiving blood transfusion in the past two weeks); platelets ≥90×10^9^/L; total serum bilirubin ≤1.5×ULN; AST and ALT ≤1.5 ULN; serum creatinine, blood urea nitrogen within normal limits, fasting blood glucose ≤126 mg/dL or ≤7.0 mmol/L.

7) According to the researcher's judgment, it is believed that the patient should be able to follow the study procedures, restrictions, and requirements, exhibit good compliance, and be willing to communicate.

8) Women of childbearing age and their family members must agree and sign an informed consent form to participate in this study.

## 3.2 Exclusion criteria

1) The subjects have undergone treatments such as chemotherapy, immunotherapy, traditional Chinese medicine, or surgery.

2) Participated in other clinical trials involving investigational drugs in the 28 days preceding the first dose of study treatment; or received prior anti-tumor therapy including, but not limited to, chemotherapy, radiation therapy, or targeted therapy;

3) Previously received treatment with immune checkpoint inhibitors.

4) Having undergone major surgery (excluding biopsies) or having a surgical incision that has not completely healed within the first four weeks of initiating drug therapy;

5) Significant pleural effusion, pericardial effusion, or ascites will require repeated drainage within the first two weeks prior to the initial administration of the drug under investigation.

6) Excluding malignant tumors during the activity period; excluding basal cell carcinoma or ductal carcinoma in situ after radical surgery.

7) A history of autoimmune disease with potential for relapse or active autoimmune disease (Appendix 4). Patients with the following diseases do not need to be excluded and may proceed with further screening: a. well-controlled type I diabetes mellitus; b. hypothyroidism (as long as being treated with replacement therapy only); c. well-controlled celiac disease; d. localized skin diseases not requiring systemic treatment (such as vitiligo, psoriasis, alopecia).

8) History of HIV infection or serious chronic or active infection requires systemic antimicrobial, antifungal, or antiviral therapy, including tuberculosis infection, etc. Patients who have a history of active tuberculosis infection within ≥1 year before screening should also be excluded, unless evidence is provided to demonstrate completion of appropriate treatment.

9) Screening period HBV DNA ≥ 10^4^ copies/mL;

10) History of interstitial lung disease, drug-induced interstitial lung disease, radiation pneumonitis, symptomatic interstitial lung disease, or evidence of active pneumonia on chest CT scan within 4 weeks before initiation of drug therapy for the first time;

11) Cardiovascular diseases of significant clinical relevance include but are not limited to acute myocardial infarction within 6 months before enrollment, severe/unstable angina pectoris, cerebrovascular accident or transient ischemic attack, and heart failure (New York Heart Association class II or higher; Appendix 3). Besides beta blockers or digoxin, treatment with other antiarrhythmic drugs is needed for arrhythmias; repeat electrocardiograms with a QTcF interval> 450 milliseconds (ms); hypertension that is not well controlled with antihypertensive medications (systolic blood pressure >150 mmHg, diastolic blood pressure >100 mmHg);

12) There is a thyroid dysfunction that prevents the thyroid function from staying within the normal range.

13) Used immunosuppressive drugs within 2 weeks prior to the first administration of the study drug, excluding local or systemic corticosteroids at a dose not exceeding 10 mg/day methylprednisolone or its equivalent.

14) Has undergone allogeneic stem cell transplantation or organ transplantation in the past;

15) Received live vaccines within the first 4 weeks prior to the drug therapy initiation; (Note: Seasonal injectable influenza vaccines are usually inactivated vaccines and therefore permitted for use. Nasal vaccines are live vaccines and thus not allowed for use).

16) History of severe allergic reactions to chimeric or humanized antibodies or fusion proteins;

17) Any other disease, metabolic disorder or abnormal laboratory findings, alcohol/drug abuse or dependency, the investigator has reason to suspect that the patient is not suitable for receiving study drug treatment, or will affect the interpretation of the study results, or put the patient at high risk.

## 3.3 Rejection criteria

If the participant selected for this experiment falls into one of the following circumstances, they should be considered for exclusion:

1) After being selected as a participant, it was found that the participant did not meet the inclusion/exclusion criteria at the time of selection and was mistakenly included in the trial.

2) Subjects who seriously violated the requirements of the experimental protocol.

## 3.4 Trial exit criteria

If any of the following situations occurs during the experiment, the researcher should arrange for termination and withdrawal from the trial. Before withdrawing from the trial, the researcher must complete the most recent assessment of all indicators. Detailed records of the reasons for subject withdrawal and the date must be kept.

1) During the experiment, it was found that the subjects selected did not meet the criteria for inclusion or exclusion.

2) Progression of malignant tumors at any time.

3) Due to serious adverse events, it must be stopped.

4) The subjects developed intolerable toxic reactions.

5) Concurrent with other ailments, researchers deem it inappropriate to continue with this clinical study.

6) The participant refused to continue with the clinical trial and withdrew voluntarily.

7) The subject is non-compliant, unable to adhere to the medication schedule or the study protocol.

8) The use of other adjuvant anti-tumor therapies impacts the evaluation of results.

9) The subject has died.

10) Lost visit.

11) The applicant may terminate the study for any reason or discontinue the participation of this subject in the study.

# **4. Investigational medicinal product**

## 4.1 Introduction to tislelizumab

Tislelizumab is a monoclonal antibody for intravenous administration in a single-use vial (20R glass vial, USP Type I) containing 10 mL of isotonic solution for a total of 100 mg of antibody. Tislelizumab is aseptically filled into single-use vials with Fluoro® protective coated butyl rubber stoppers and aluminum caps. Each vial is individually packaged in a single-use outer box.

### 4.1.1 Pharmacology

Tislelizumab (also known as BGB-A317) is a humanized immunoglobulin G4 (IgG4) variant monoclonal antibody against PD-1 that is currently marketed in China for the treatment of a variety of human malignancies. Tislelizumab works by binding to the extracellular domain of human PD-1 with high specificity and affinity (discrete constant [KD] = 0.15 nM). It inhibits PD-1-mediated negative regulation of T cells by competitively and simultaneously preventing PD-1 from binding to PD-L1 and programmed cell death protein ligand-2 (PD-L2). In in vitro cell assays, tislelizumab was observed to sustainably and dose-dependently enhance the functional activity of human T cells and activated primitive peripheral blood mononuclear cells. In addition, tislelizumab has shown antitumor activity in several xenograft models established by co-injecting peripheral blood mononuclear cells with human cancer cells (A431 [epithelioid carcinoma] or tumor fragments (BCCO-028 [colon carcinoma]) into immunocompromised mice. In vitro, the IgG4 variant has been shown to bind with very low affinity to the fragment crystallizable region (Fc) receptor IIIA (FcγRIIIA) and complement 1q, a subunit of complement 1, suggesting that antibody-dependent cellular cytotoxicity (ADCC) or complement-dependent cytotoxicity (CDC) effects are minimal or absent (Labrijn et al 2009).

### 4.1.2 Absorption, distribution, metabolism and excretion

The pharmacokinetics (PK) of tislelizumab were studied in cynomolgus monkeys with single-dose doses of 3, 10, or 30 mg/kg and repeated-dose doses of 10 mg/kg once weekly for 5 consecutive doses. The route of administration was intravenous infusion. The systemic exposure of tislelizumab increased proportionally with dose, and no gender differences or drug accumulation were observed. After a single dose of 3, 10, or 30 mg/kg, the half-life (t1/2) ranged from 74 to 183 hours; the maximum serum concentration (Cmax) ranged from 90 to 999 μg/mL, the area under the curve (AUC0-1008h) ranged from 12,322 to 163,755 h*μg/mL; and the volume of distribution (Vd) ranged from 22 to 52 mL/kg. The t_1/2_ value of tislelizumab in cynomolgus monkeys supports the use of a once every 2 weeks (every other week) dosing schedule in repeated-dose toxicology studies.

### 4.1.3 toxicology

The toxicity and safety of tislelizumab were evaluated in single-dose toxicology studies in mice and cynomolgus monkeys and in a 13-week repeat-dose toxicology study in monkeys. Tissue cross-reactivity was evaluated in normal frozen tissues from humans and monkeys. Cytokine release assays were evaluated using fresh human whole blood cells. Pivotal toxicity studies were conducted in accordance with Good Nonclinical Research Practice guidelines. Single-dose regimens ranged from the expected human dose to a dose 10-fold higher than the highest expected human dose, and repeat-dose regimens ranged up to a dose 3-fold higher than the highest expected human dose. Cynomolgus monkeys are the only relevant species based on target sequence homology and binding activity. Overall, no significant toxicity was observed in the toxicity studies in mice or monkeys. Tissue cross-reactivity was not observed in either human or monkey tissues, and no effects on cytokine release were observed in human whole blood assays. Toxicokinetic properties were fully characterized, with a dose-proportional increase in systemic exposure, with no significant accumulation or sex differences. Immunogenicity was observed, but no significant immunotoxicity or effects on systemic exposure were observed. In a 13-week toxicity study in monkeys, the no-observable-adverse-effect dose of tislelizumab was considered to be 30 mg/kg. Therefore, the safety profile of tislelizumab was considered sufficient to support the current study.

### 4.1.4 Clinical data

Tislelizumab is an anti-cancer drug that can be used as a single agent or in combination with other therapies to treat a variety of malignancies in different geographic populations. The overall safety experience of tislelizumab is based on the experience of 1,705 patients who received the drug as a single agent or in combination with other therapies as of May 20, 2019. As a single agent, the drug has a similar safety profile in the treatment of different types of tumors. There was no specific pattern between the incidence, severity or causality of adverse events (AEs) and the dose level of tislelizumab. The safety profile of tislelizumab monotherapy is similar to that observed in other PD-1 inhibitors. The initial data collected in these studies show that tislelizumab has anti-tumor activity against a variety of tumor types. Anti-tumor activity was observed in the dose range used in evaluable patients. In the Phase III controlled study, the safety profile of tislelizumab monotherapy was acceptable in the context of observed clinical efficacy and could be managed by the recognized safety guidelines. For combination studies, the safety profile of tislelizumab was generally consistent with monotherapy, showing safety and well-tolerated status in combination with other agents and in a variety of different chemotherapy applications. Preliminary antitumor activity of tislelizumab has been observed in multiple tumor types when used in combination with chemotherapy and other agents.

Immune-related adverse events (irAEs) were observed in 23.1% and 16% of patients in the BGB-A317-001 and BGB-A317-102 studies receiving tislelizumab monotherapy, respectively: Immune-related adverse events that occurred in at least 2% of patients in the overall population are detailed in Table 2 below.

Table 2. Immune-related TRAEs assessed by investigators for tislelizumab

|  | **BGB-A317-001 Study N=451 n (%) ^a^** | **BGB-A317-102 Study N=300 n (%) ^b^** |
| --- | --- | --- |
| Thyroid dysfunction | 39 (8.6) | 25 (8.3) |
| Skin reactions | 39 (8.6) | 9 (3.0) |
| Pneumonia | 12 (2.7) | 7 (2.3) |
| Hepatitis | 10 (2.2) | 5 (1.7) |
| Colitis | 9 (2.0) | 0 |

a As of October 27, 2018. b As of December 1, 2018.

Among these irAEs, immune-mediated skin reactions were reported more frequently in the overall population of BGB-A317-001 compared with study BGB-A317-102. The severity of these events was mostly grade 1 or 2, with fewer grade 3 events.

In the BGB-A317-001 study, potential immune-related treatment-emergent adverse events (irTEAEs) assessed as grade ≥3 were: grade 3 pneumonitis and increased ALT (6 patients each, 1.3%), diarrhea and colitis (3 patients each, 0.7%), increased GGT and hyperglycemia (2 patients each, 0.4%). All other potential irTEAEs assessed as grade ≥3 were single-patient reported events, including a grade 4 diabetic ketoacidosis event reported in a patient with adenoid cystic carcinoma, and 2 grade 5 events (acute hepatitis reported in a patient with hepatocellular carcinoma (HCC) and pneumonitis reported in a patient with NSCLC).

In Study BGB-A317-102, the only grade 4 event reported was a case of skin adverse reaction. No deaths due to irAEs occurred in Study BGB-A317-102.

## 4.2 Instructions for using investigational medicinal product

### 4.2.1 PD-1 antibody

Tislelizumab 200 mg will be administered on Day 1 of each 21-day cycle (once every 3 weeks). Tislelizumab will be administered by intravenous infusion using an IV catheter containing a sterile, non-pyrogenic, low protein binding, 0.2 or 0.22 micron diameter inline filter or auxiliary filter.

The first infusion (Day 1 of Cycle 1) needs to be completed within 60 minutes; if well tolerated, subsequent infusions can be administered within 30 minutes, which is the minimum allowed infusion time. Tislelizumab should not be administered concurrently with any other medication.

### 4.2.2 Chemotherapy drugs

Paclitaxel: Taxol will be administered intravenously at a dose of 175 mg/m^2^ on day 1 of each 21-day cycle, by IV infusion, every 3 weeks for 3 cycles.

Cisplatin: Cisplatin 60 mg/m^2^ will be administered as an IV infusion over 2 hours on day 1 or 2 of each cycle for 3 cycles. Multiple doses of cisplatin are not permitted. All patients should receive adequate hydration (including premedication) and a diuretic. The recommended premedication for cisplatin is dexamethasone 7.5 mg once daily for 3 days. Urine output must be maintained at > 2000 mL within 24 hours after the infusion.

Carboplatin: Carboplatin AUC5 will be administered as an IV infusion over 1 hour on day 1 or 2 of each cycle for 3 cycles.

Table 3. Dose selection and administration time for each patient

| **Investigational Medicinal Product** | **Dose** | **Dosing frequency** | **Route of administration** | **Treatment duration** |
| --- | --- | --- | --- | --- |
| Tislelizumab | 200 mg | Day 1 of 21-Day Cycle | Intravenous injection | 3 cycles |
| Paclitaxel | 175 mg/m^2^ | Day 1 or 2 of 21-Day Cycle | Intravenous injection | 3 cycles |
| Cisplatin | 60 mg/m^2^ | Day 1 or 2 of 21-Day Cycle | Intravenous injection | 3 cycles |
| Carboplatin | AUC5 | Day 1 or 2 of a 21-day cycle | Intravenous injection | 3 cycles |

### 4.2.3 Drug dose delay or adjustment

Dose holds are defined as interruptions in the treatment regimen (i.e., drug administration is delayed outside the visit window). Dose interruptions are defined as interruptions in infusions.

Every effort should be made to administer study medications according to the planned dose and schedule. If severe toxicity occurs, doses may be delayed and/or reduced according to the guidelines described below. The reasons for dose modifications or delays, supportive measures taken, and outcomes will be documented in the patient's medical record and documented in the CRF.

The dose modification guidelines in this section do not replace clinical judgment. The investigator may withhold or modify the dose for other reasons (e.g., adverse events, weight loss, laboratory findings) at his or her discretion.

Interruptions are permitted if they are due to medical/surgical events or other reasons unrelated to study treatment (e.g., elective surgery, unrelated medical events, patient leave, and/or vacation). The investigator determines whether the patient should continue study treatment within 3 weeks of a planned interruption.

## 4.3 General guidance for dose adjustment

Tislelizumab may be withheld. Dose adjustments of chemotherapy should be made based on the prescribing information and local practice, based on the treating physician’s clinical judgment.

1) For AEs that have occurred at baseline, dose adjustments will be made based on changes in toxicity grade, based on the investigator’s judgment. For example, if a patient has grade 1 asthenia at baseline and it increases to grade 2 during treatment, it will be considered a change relative to grade 1, and dose adjustments will be made to reduce to grade 1 toxicity.

2) When several toxicities of varying severity occur simultaneously, dose adjustments should be made based on the highest grade observed.

3) If the investigator believes that the toxicity is due to only one part of the study treatment, and the dose of that part has been withheld or adjusted according to the guidelines below, the other parts may continue to be administered if there are no contraindications.

4) If chemotherapy-related toxicity occurs, chemotherapy will be withheld until the toxicity reaches baseline or ≤ grade 1 (whichever is more severe) before the next chemotherapy dose, with the exception of alopecia, grade 2 fatigue, or other adverse events that the investigator believes will not affect the safety assessment of the study drug. If one part of the chemotherapy regimen is suspended due to an AE, the other part will also be suspended until resolution of the AE allows continuation of both parts of the chemotherapy regimen. Tislelizumab should continue to be administered as planned. If the AE resolves within 10 days, chemotherapy will be administered. Administration of chemotherapy and tislelizumab or placebo will be resynchronized in the next cycle, which will be scheduled based on the chemotherapy dosing date. If the AE does not resolve within 10 days, chemotherapy will not be given for that cycle. If the AE resolves within 21 days, chemotherapy and tislelizumab will be administered as planned on the first day of the next treatment cycle.

5) If tislelizumab-related toxicity occurs, tislelizumab will be withheld until toxicity reaches baseline or ≤ Grade 1 (whichever is more severe), at which time the next dose of tislelizumab or placebo can be continued, except for alopecia, Grade 2 fatigue, or other adverse events that the investigator deems will not affect the safety assessment of the study drug. Chemotherapy should be administered as planned. If the AE resolves within 10 days, tislelizumab will be administered. Administration of chemotherapy and tislelizumab will be resynchronized in the next cycle, which will be scheduled based on chemotherapy dosing dates. If the AE does not resolve within 10 days, tislelizumab should not be administered in the current cycle. If the AE resolves within 21 days, tislelizumab and chemotherapy will be administered as originally planned on the first day of the next treatment cycle.

### 4.3.1. Dose suspension or adjustment of tislelizumab

Tislelizumab dose reductions were not performed in this study. Patients may temporarily discontinue study treatment if they experience toxicities that are thought to be related to tislelizumab and require a discontinuation of treatment. If the discontinuation is ≤10 days, tislelizumab will be administered; if the discontinuation is greater than 10 days, tislelizumab will be omitted for that cycle and the next cycle will be administered as planned as long as the AE resolves within 21 days. Patients should resume tislelizumab treatment as soon as possible after the adverse event resolves to baseline or Grade 1 (whichever is more severe) and within 12 weeks after the last dose of tislelizumab. If a patient is unable to resume tislelizumab/placebo within 12 weeks of the last dose of tislelizumab, the patient should discontinue treatment.

### 4.3.2 Suspension, interruption or adjustment of chemotherapy dose

The chemotherapy dose modification guidelines, as described below, are based on the severity of toxicity and the patient's risk-benefit assessment, with the goal of maximizing patient compliance and supportive care. The chemotherapy dose required is calculated using baseline body weight. If the patient's weight changes ≥10% from baseline (the newly recorded weight will be the new baseline weight), a dose modification is required. If the weight change is <10%, no chemotherapy dose modification is necessary. Study drug-related toxicity must resolve to baseline or ≤ Grade 1 (whichever is more severe) before the next dose, with the exception of alopecia or Grade 2 fatigue or other adverse events that the investigator believes will not affect the safety assessment of the study drug. If one part of the chemotherapy regimen is interrupted due to an AE, the other part will also be interrupted until resolution of the AE allows continuation of both parts of the chemotherapy regimen. A maximum of 2 dose reductions may be made for cisplatin/carboplatin and a maximum of 2 dose reductions may be made for paclitaxel. Once a dose is reduced, it should be maintained at the reduced dose for all subsequent dosing sessions or further reduced if necessary. There is no dose escalation portion of this study. If a further dose reduction is required, the chemotherapy agent must be discontinued. Dose modifications of chemotherapy agents will be made according to Table 2. Chemotherapy treatment may be delayed for up to 21 days if the reason for delay is toxicity/AE. All subsequent chemotherapy doses must be rescheduled based on the date of the last chemotherapy dose (Table 4). If any chemotherapy drug is delayed for more than 2 cycles (6 weeks) from the expected treatment date, or if cisplatin/carboplatin is reduced by 2 doses or paclitaxel is reduced by 2 doses, the chemotherapy regimen should be permanently discontinued.

Table 4. Guidelines for dose adjustment levels

|  | **Initial dose** | **Dose level -1** | **Dose level -2** | **Dose Level -3** | **Dose level -4** |
| --- | --- | --- | --- | --- | --- |
| Tislelizumab | 200 mg | Not allowed. | Not allowed. | Not allowed. | Not allowed. |
| Paclitaxel | 175 mg/m^2^ | 135 mg/m^2^ | 90 mg/m^2^ | Discontinue medication. | Discontinue medication. |
| Cisplatin | 60 mg/m^2^ | 50 mg/m^2^ | 40 mg/m^2^ | Discontinue medication. | Discontinue medication. |
| AUC (Area Under the Curve) | 5 | 4.5 | 4 | Discontinue medication. | Discontinue medication. |

Table 5. Chemotherapy doses for hematologic toxicity of paclitaxel combined with cisplatin/carboplatin

| **Adverse event** | | **Therapy** |
| --- | --- | --- |
| Neutropenia; definite infection | | For the first occurrence of neutropenia or confirmed infection, antibiotic therapy should be initiated at a dose level of once.  If the reaction occurs a second time after the dose, the patient must receive prophylactic antibiotic treatment in subsequent cycles.  3. If it occurs for the third time, stop chemotherapy. |
| neutropenia | Level 3 (0.5-0.99 × 10^9^/L) | Chemotherapy should be postponed until recovery to Grade ≤1 (≥1.5 x 10^9^ /L); restart with full dose. |
|  | 4th level (<0.5-0.99 × 10^9^/L) | Chemotherapy postponed until recovery to ≤Grade 1; all subsequent doses at one dose level. |
| Thrombocytopenia | Level 1 | Postpone chemotherapy until recovery; restart at full dose. |
|  | ≥ Level 2 | Chemotherapy delayed until recovery; all subsequent doses at one dose level. |

Table 6. Recommended doses for non-hematologic toxicity

| **Toxicity** | **Level** | **Therapy** |
| --- | --- | --- |
| Nephrotoxicity | ≥ Level 1 | Delay chemotherapy until recovery to Grade 0 or baseline levels, dose of one dose of Cisplatin. |
| Ototoxicity | Level ≥2 | Postpone chemotherapy until recovery to Grade 1 or baseline level, decrease cisplatin dose by 1 level, patients who still need dosage after 2 doses of cisplatin will discontinue cisplatin treatment. |
| Neurological degeneration. | Level 2 | All subsequent doses of cisplatin are at a single dose level. |
|  | 3 levels | Discontinue cisplatin. |
|  | Level 4 | Discontinue all chemotherapy. |
| Toxicity in other organs | 2nd grade | Delay chemotherapy until the alleviation reaches ≤ Grade 1 or baseline level ^a^ . |
|  | Level 3 or level 4. | Postpone chemotherapy until recovery to ≤ Grade 1 or baseline level ^a^ , with all subsequent doses reduced by 1 dose level. |

Note: It may be decided by researchers to implement supportive measures/therapy and/or secondary prevention in the next cycle, instead of dosage, if it is in the best interest of the patient and in accordance with local conventions. The dosage recommendations provided are for reference only. a. Skin reactions, paronychia, hair loss, fatigue, and nausea/vomiting may have already alleviated to Grade 2 or baseline level.

### 4.3.3. Criteria for stopping chemotherapy regimen

In addition to the above, chemotherapy should be discontinued if any of the following occur:

1) Any grade 4 peripheral neuropathy.

2) Persistent grade 3 paresthesias.

3) Grade 3 or 4 drug-related thrombocytopenia associated with clinically significant bleeding

4) Discontinuation is indicated for any drug-related liver function test abnormalities that meet the following criteria:

• AST or ALT >5 to 10×ULN for >2 weeks

• AST or ALT >10×ULN

• Total bilirubin >5×ULN

• Current AST or ALT >3×ULN and/or total bilirubin >2×ULN

5) Cisplatin should be discontinued for any cisplatin-related decrease in creatinine clearance to <30 mL/min (using the Cockroft-Gault formula)

6) Discontinuation is indicated for any recurrence of any drug-related

AE after 2 dose reductions of cisplatin/carboplatin and 2 dose reductions of paclitaxel.

7) If any grade 3 or 4 drug-related allergic or infusion reaction occurs, the drug assessed as causing the reaction should be discontinued. If the drug is assessed as not related to allergic reactions or infusion reactions, it may be continued.

8) Any grade 4 AE that the investigator deems to be related to the study drug and is not amenable to management by dose reduction requires discontinuation. If the drug is not assessed as related to the event, it may be continued.

9) If any chemotherapy drug is suspended for more than two cycles (6 weeks) from the original start date of chemotherapy, the chemotherapy drug needs to be permanently discontinued.

For toxicities not listed above, the investigator will use medical judgment based on the patient's health status and local standards to determine whether the chemotherapy regimen should be discontinued.

## 4.4. Concomitant medication and restricted medication

### 4.4.1. Concomitant medication

Concomitant medications include any prescription or over-the-counter medications used within 28 days prior to the first study drug treatment and during the safety follow-up period. All concomitant medications should be recorded in the CRF.

Most concomitant medications and concomitant treatments are allowed as long as they are deemed necessary by the investigator's judgment and are consistent with local standard medical care for supportive care (e.g., antiemetics, antidiarrhea) and in the interest of the patient. Patients should receive adequate supportive care as needed, including erythropoietin and other hematopoietic growth factors, blood transfusions and blood products, antibiotics, antiemetics, and/or other necessary and applicable medications.

Systemic corticosteroids used to control irAEs must be tapered (see Appendix 5 for details) and reach a non-immunosuppressive dose (prednisone ≤10 mg/day or equivalent) before the next dose of tislelizumab. Short-term use of steroids is allowed as prophylactic treatment (e.g., in patients with allergies to diagnostic imaging contrast agents).

### 4.4.2 Restricted medication

During the entire study, the subjects cannot receive other specific anti-tumor drugs at the same time, including chemotherapy, hormone therapy, immunotherapy, or standard treatment or investigational drugs for the treatment of cancer [including Chinese herbal medicine and Chinese patent medicine]).

Live vaccines are prohibited from 28 days before randomization to 60 days after the last dose of study drug.

Immunosuppressants cannot be received at the same time (systemic glucocorticoids are allowed to treat drug-related adverse events).

# **5. Study procedure**

5.1 The study subjects must be judged by the investigators as suitable for treatment with cisplatin or carboplatin plus paclitaxel.

5.2 Patients who meet the inclusion criteria will receive the following treatment options: Tislelizumab combined with cisplatin/carboplatin + paclitaxel, 3 courses of treatment, radical resection for CR/PR/SD patients. PD patients are excluded from the group and receive radical concurrent chemoradiotherapy.

5.3 Neoadjuvant therapy is a medication cycle of 3 weeks until any of the following situations occurs (whichever occurs first):

1) completion of 3 cycles of treatment;

2) intolerable toxicity;

3) disease progression (based on RECIST 1.1);

4) withdrawal of informed consent, loss of follow-up or death;

5) the total duration of neoadjuvant therapy exceeds 12 weeks.

5.4 Perform imaging assessment once before the third cycle of neoadjuvant therapy and before surgery: 1) Before the third cycle, if the patient does not have PD, continue treatment as planned; if PD, the investigator will determine whether to give follow-up treatment;

2) Preoperative assessment, if the patient meets the surgical indications, the investigator will select the appropriate time for radical cervical resection according to the plan; if the patient is not suitable for surgery, the investigator will determine whether to give follow-up treatment.

5.5 If the patient is unable to complete 3 cycles of neoadjuvant therapy or radical cervical resection due to drug-related AEs, the investigator will determine whether to give follow-up treatment.

# **6. Biomarkers and tumor tissue samples**

The transportation, storage, and processing of blood, archived tumor tissue, fresh tumor tissue and residual tumor tissue for biomarker assessment will be managed by the investigator. Other exploratory predictive biomarkers associated with the clinical benefit of this study may be used to assess, such as tumor mutation burden (TMB), expression of PD-L1, and other immune-related genes/proteins. Fresh tumor tissue samples are obtained from pre-treatment and post-surgery surgical specimens. Plasma samples are collected at three-time points during treatment, including before the neoadjuvant therapy (pre-treatment sample), before the second cycle of neoadjuvant therapy (on-treatment sample), and before surgery (post-treatment sample). Destroyed 5 years after the end of this study. Analysis of blood and tissue specimens is limited to the use described in the informed consent form of the subjects of this study and will not be used for other purposes.

Time points for collecting hematological samples:

• Before treatment, collect tumor tissue and 10 mL of peripheral blood;

• Each time the efficacy is evaluated, draw 10 mL of peripheral blood;

• When the disease progresses, draw 10 mL of peripheral blood;

• For patients with complete remission, draw 10 mL of peripheral blood 3-4 weeks after the last treatment

# **7. Description of study endpoints**

## 7.1 Primary study endpoints

• pCR rate after radical cervical surgery;

## 7.2 Secondary study endpoints

• ORR assessed by imaging after 3 cycles of neoadjuvant therapy, that is, the proportion of patients who achieved CR or PR, according to RECIST 1.1;

• Safety: according to the degree of CTCAE version 5.0 grading;

## 7.3 Exploratory study endpoints

Explore biomarkers predicting efficacy in tumor tissue and peripheral blood, including PD-L1 expression and ctDNA

# **8. Detailed evaluation criteria**

## 8.1 Tumor and efficacy assessment

1) Baseline tumor imaging examinations will be performed within 14 days before enrollment, including plain and contrast-enhanced pelvic MRI sequences and chest and abdominal contrast-enhanced CT scans. In cases where patients will have metallic implants or other contraindications for MRI, contrast-enhanced CT scans of the pelvis will be used as an alternative. Standard treatment tests or examination results performed before obtaining informed consent and ≤14 days before enrollment can be used for screening purposes instead of repeating standard treatment tests.

2) During the treatment period, according to RECIST 1.1, plain and contrast-enhanced pelvic MRI sequences will be performed. In cases where patients will have metallic implants or other contraindications for MRI, contrast-enhanced CT scans of the pelvis will be used as an alternative.

i. Evaluation before the start of the third cycle, if the patient does not have PD, the treatment will continue as planned; if the patient has PD, the investigator will decide whether to give subsequent treatment;

ii. Preoperative evaluation, if the patient meets the surgical indications, the investigator will choose the appropriate time for radical hysterectomy according to the plan; if the patient is not suitable for surgery, the investigator will decide whether to give subsequent treatment

3) After radical surgery, the investigator will evaluate the pathological staging after neoadjuvant treatment based on the pathological results of the surgical specimens.

4) Perform chest, whole abdomen and pelvic computed tomography (CT) scans at 3, 6, 12, 18 and 24 months after radical surgery (if necessary, the investigator will determine whether to perform enhancement) until the patient has disease progression, death, withdrawal of informed consent, loss to follow-up, end of study follow-up or start of new anticancer treatment (surgery), whichever occurs first.

The investigator will evaluate the response using RECIST 1.1 (see Appendix 6). At each tumor assessment after enrollment, all lesions should be reassessed and the same imaging assessment method as that used for screening should be used to ensure internal consistency between visits.

The investigator must read the film before the third cycle of medication to determine whether the patient is eligible to continue to receive the third cycle of neoadjuvant therapy, or other standard treatments (progression, and cannot receive radical surgery). Similarly, the investigator must review the tumor assessment scan results before surgery to assess the response and confirm resectability.

If AE fails to complete 3 cycles of neoadjuvant therapy (the total duration of neoadjuvant therapy does not exceed 12 weeks), but the investigator assesses that the patient meets the indications for radical surgery, the patient will continue to undergo surgery within the specified time limit and will still be considered eligible for all treatments and evaluations required by the study from the last medication of AE to surgery time ≤ 6 weeks. If the above time limit is exceeded, the reason must be explained and the deviation from the protocol must be recorded). If the investigator determines that the patient is physically unable to undergo radical surgery, the study treatment will be stopped, the patient will be given an end-of-treatment visit, and the patient will continue to receive other treatments selected by the investigator.

## 8.2. Safety assessment

### 8.2.1 Vital signs

Vital signs include temperature (°C), pulse, and blood pressure (systolic and diastolic) after the patient has rested quietly for 10 minutes. Height (baseline only) and weight should be measured and recorded in the CRF.

Vital signs must be monitored within 1 hour before, during, and within 1 hour after the first two tislelizumab infusions; starting with cycle 3, vital signs will be collected within 60 minutes before infusion, and if clinically indicated, during and within 30 minutes after infusion.

### 8.2.2 Physical examination

During the Screening Visit, a physical examination will be performed including evaluation of the 1) head, eyes, ears, nose, throat, 2) cardiovascular, 3) skin, 4) musculoskeletal, 5) respiratory, 6) gastrointestinal, 7) neurologic, and 8) gynecologic systems. Any abnormalities found during Screening will be graded according to NCI-CTCAE version 5.0 and recorded in the CRF using the appropriate disease/condition terminology.

At follow-up visits (and as clinically indicated), a limited, symptom-directed physical examination will be performed. Changes from baseline will be recorded. New or worsening clinically significant abnormalities should be recorded as AEs in the CRF.

### 8.2.3 Eastern Cooperative Oncology Group (ECOG) Performance Status

ECOG performance status will be assessed during the study.

### 8.2.4 Laboratory safety inspection

Serum biochemistry, hematology, coagulation, urinalysis, stool analysis, infectious disease testing, pregnancy testing, thyroid function, and blood typing will be evaluated at local laboratories, some of which will be collected as specified in Appendix 1 and the Central Laboratory Sample Management Manual.

If laboratory tests required for screening are not performed within 7 days before enrollment, they should be repeated and the results reviewed before study drug administration. Hematology and serum biochemistry (including liver, kidney, and heart function tests) should be performed at the beginning of subsequent cycles and as clinically indicated as specified in Appendix 1. Investigators may use the results of local laboratories for eligibility assessments, safety monitoring, and dosing decisions. See Table 7 below

Table 7. Laboratory test items

| **Serum biochemistry test** | **Hematology** | **Coagulation** | **Urinalysis** |
| --- | --- | --- | --- |
| alkaline phosphatase  Alanine aminotransferase  Aspartate aminotransferase  Albumin  Hydrogen carbonate or total carbon dioxide ^a^  Total bilirubin  Direct bilirubin  Blood urea nitrogen or urea  magnesium  Chlorine  phosphorus  potassium  Sodium  Calcium  Creatinine  glucose  Lactate dehydrogenase  Total protein  Creatine Kinase (CK)  CK-MB^c^ | Red blood cell count  hemoglobin  Hematocrit  White blood cell count  Platelet count  Neutrophil count.  Eosinophil count  Eosinophil count  Monocyte count  Lymphocyte count | Prothrombin time  Partial thromboplastin time or activated partial thromboplastin time  Internationalization ratio  **Infectious disease examination**  HIV antibody  Hepatitis B both halves  HCV antibodies  **Thyroid function**  Free T3  Free T4  Serum total thyroxine TT4  Serum total triiodothyronine (TT3)  Test for thyroid stimulating hormone (TSH) | pH  Specific gravity  glucose  protein  ketone  Occult blood  24-hour protein ^b^  **Stool routine**  Color  Traits  white blood cell inspection  blood smear  Fecal occult blood test |

Abbreviations: CK-MB, creatine kinase cardiac isoenzyme;

a Measure this if considered regional standard of care.

b For routine urinalysis, if urine protein is ≥2+ by dipstick, obtain a 24-hour urine sample for total protein and a random urine sample for total protein and creatinine to determine the protein-to-creatinine ratio.

c If the CK-MB component is not available, assess troponin I and/or troponin T instead.

**Electrocardiogram**

For safety monitoring purposes, the investigator must review all 12-lead ECG records, sign and date them. Paper or electronic versions of the ECG will be kept at the research center as part of the patient's permanent research file. The 12-lead ECG will be performed by a qualified physician at the time points specified in the "Trial Flow Chart". All ECG examinations are required to be performed after the subject has rested quietly for at least 10 minutes. The content includes at least: heart rate, QT, QTcF and P-R time. The screening period requires 3 examinations (each interval is at least 5 minutes). QTcF can be calculated according to the formula QTcF=QT/heart rate 0.33. If the subject has symptoms such as chest pain and palpitations, the investigator may, at his discretion, perform an ECG and/or myocardial enzyme spectrum test.

**Cardiac Ultrasound**

Qualified physicians are responsible for the screening period echocardiogram examination and LVEF measurement.

Pulmonary function: A qualified physician will be responsible for performing pulmonary function tests during the screening period and evaluating the tolerance of radical surgery.

## 8.3. Adverse Events

### 8.3.1 Definition of adverse events

Medication and radical surgery AEs will be graded and recorded throughout the study according to the NCI-CTCAE, version 5.0 and Clavien-Dindo scoring systems. Characterization of toxicity will include severity, duration, and time to onset. All AEs, including SAEs, will be collected as described below.

An AE is defined as any unfavorable and unexpected sign (including abnormal laboratory test results), symptom, or disease (new or worsening) related to the study drug or procedure.

Examples of adverse events include:

• An exacerbation of a chronic or intermittent pre-existing condition, including an increase in severity, frequency, duration, and/or associated with a significantly worse outcome

• A new condition discovered or diagnosed after study drug administration or related to a procedure, even if these conditions may have existed before the start of the study

• Signs, symptoms, or clinical sequelae of an interaction

• Signs, symptoms, or clinical sequelae suspected to be related to an overdose of the study drug, concomitant medication, or procedure (overdose itself should not be reported as an AE or SAE)

If an AE or SAE occurs, the investigator is responsible for reviewing all documentation related to the AE or SAE (e.g., hospital progress notes, laboratory results, and diagnostic reports). The investigator will then record all information related to the AE or SAE in the CRF.

### 8.3.2 Severity assessment

The investigator will assess the severity of each AE and SAE reported during the study. Drug-related or procedure-related AEs and SAEs should be evaluated and graded according to the NCI-CTCAE version 5.0 or Clavien-Dindo score.

The definitions of specified toxicities in the NCI-CTCAE are as follows:

• Grade 1: Mild: Asymptomatic or mildly symptomatic; clinical or diagnostic observation only; no intervention required

• Grade 2: Moderate: Indicative of minimal, local or noninvasive intervention; limitation of age-appropriate instrumental activities of daily living

• Grade 3: Severe or medically significant but not immediately life-threatening; hospitalization or prolonged hospitalization required; disability; limitation of self-care activities of daily living.

• Grade 4: Life-threatening consequences; urgent treatment required

• Grade 5: AE associated with death

Note: The terms "severity" and "seriousness" are not synonymous. Severity is a measure of intensity (e.g., the grade of a particular AE, mild [grade 1], moderate [grade 2], severe [grade 3], or life-threatening [grade 4]), and severity is categorized according to criteria based on regulatory definitions.

Grades of procedure-related AEs as specified in Clavien-Dindo are defined as follows:

Complication severity is graded according to the complication scoring system (Clavien–Dindo classification), with grade IIIA and above being severe complications.

• Grade 1: Postoperative complications of unwanted medications, surgery, endoscopy, and reflex interventions, but including medications for antiemetics, antipyretics, analgesics, diuretics, electrolytes, physical therapy, and bedside open wound infection

• Grade 2: Patients requiring medications other than Grade 1 medications, including wound infection requiring antibiotics, transfusions, and total parenteral nutrition

• Grade 3: Surgical, endoscopic, or radiological interventions required: Grade 3a: no general anesthesia required; Grade 3b: general anesthesia required

• Grade 4: Life-threatening complications (including central nervous system complications) requiring intermittent monitoring or intensive care management: 4a: single organ dysfunction (including dialysis); 4b: multiple organ dysfunction

• Grade 5: death

### 8.3.3 Causality assessment

The investigator is obligated to use best clinical judgment to assess the relationship between the study drug or procedure and the occurrence of each AE or SAE. Other causes, such as the natural history of the underlying disease, concomitant treatment, other risk factors, and the temporal relationship of the AE or SAE to the study drug, should be considered and investigated. Investigators should refer to the study drug labeling when determining drug-related assessments.

It is important that the investigator assess the causality of each SAE as it occurs, as causality assessment is one of the criteria used in determining regulatory reporting requirements. Investigators may change their view of causality based on follow-up information and adjust SAE reporting accordingly. Investigators are responsible for assessing the causality of each AE and classifying it as definitely related, possibly related, probably not related, definitely not related, or not determined. In making this assessment, multiple factors should be considered, including:

• The timing of the AE to the administration of the study treatment/procedure

• Whether an alternative causal agent can be found

• The mechanism of action of the study drug

• Biological plausibility

### 8.3.4 Definition of serious adverse events

An SAE is any adverse medical event, regardless of dose, that:

• Resulted in death

• Was life-threatening

Note: The term “life-threatening” in the definition of “serious” refers to AEs in which the patient would be at risk of death if the AE occurred. It does not refer to AEs that could have resulted in death if the event had been more severe.

• Required hospitalization or prolonged hospitalization

Note: Generally, hospitalization means that the patient is admitted to a hospital or emergency department (usually for at least one night) for observation and/or treatment that is not conveniently performed in a physician’s office or outpatient setting. Hospitalizations for program-required neoadjuvant therapy and for radical surgery are not classified as SAEs.

• Resulted in disability/incapacity

Note: The term “disability” refers to a significant disruption in a person’s ability to conduct a normal life. This definition does not include relatively medically minor experiences, such as uncomplicated headaches, nausea, vomiting, diarrhea, influenza, and unexpected injuries (e.g., sprained ankles), which may interfere with or impact daily life but are not severe enough to be disruptive.

• Congenital malformation/birth defect in offspring

• An AE that the investigator considers to be medically significant based on medical judgment (e.g., may pose a risk to the patient or may require medical/surgical intervention to prevent one of the above outcomes)

The following will not be considered SAEs:

• Hospitalization for elective treatment of a pre-existing condition that has not worsened from baseline

• Hospitalization for social/convenience reasons

• Hospitalization for treatment scheduled for the target disease of the study, including hospitalization for infusion support or convenience

Adverse Event Reporting

Only SAEs should be reported after informed consent is signed but before study drug administration begins.

After study drug administration begins, all AEs will be reported regardless of relationship to study drug or curative surgery until 28 days after the last dose of chemotherapy, 90 days after tislelizumab, or 90 days after curative surgery, whichever occurs later.

After treatment is discontinued, the investigator should report any SAE assessed as study related.

Serious Adverse Event Reporting

Serious adverse events must be reported immediately

If the investigator determines that the AE meets the definition of SAE in the study protocol, the event must be reported immediately (within 24 hours) to the regulatory agency, IRB/IEC, and designated personnel.

### 8.3.5 Management of adverse events of special concern

**Evaluation and recording of immune-related adverse events**

Because the use of anti-PD-1 therapy may cause autoimmune diseases, the investigator believes that immune-related AEs should be classified as irAEs and identified as irAEs in the CRF AE page until 90 days after cessation of immunotherapy or 90 days after radical surgery (whichever occurs later).

Immune-related AEs require special attention in this study. If the following events or similar events occur, the investigator should exclude other explanations (such as co-medication, infectious diseases, metabolism, toxins, PD or other tumor causes) and perform appropriate diagnostic tests, including but not limited to serological, immunological and histological (biopsy) data. If other causes are excluded, the AE requires systemic steroids, other immunosuppressants, or endocrine therapy; and the AE is consistent with an immune-mediated mechanism of action, the AE CRF should be reviewed for irAE indicators.

An exhaustive list of potential irAEs is listed in Appendix 5. Investigators should evaluate various conditions similar to the listed irAEs to determine whether they are irAEs based on the diagnostic process for reactions described in more detail in Appendix 5.

**Infusion-related reactions**

Symptoms of infusion-related reactions include fever, chills/rigidity, nausea, pruritus, angioedema, hypotension, headache, bronchospasm, urticaria, rash, vomiting, myalgia, dizziness, or hypertension. Severe reactions may include acute respiratory distress syndrome, myocardial infarction, ventricular fibrillation, and cardiogenic shock. Patients should be closely monitored for such reactions. If an infusion-related reaction occurs, immediate admission to an intensive care unit or equivalent setting and appropriate medical treatment (including epinephrine, corticosteroids, intravenous antihistamines, bronchodilators, and oxygen) are required.

Table 8 provides treatment modifications for infusion-related reaction symptoms caused by study drug.

| **NCI-CTCAE Grade** | **Treatment modifications for tislelizumab** |
| --- | --- |
| Grade 1 - Mild  Mild transient reaction; not indicative of terminal infusion; no intervention measures to hydrate | Reduce infusion rate by 50%. All exacerbations should be monitored closely. Administer medical management as needed. Subsequent infusions should be given after administration of the chemotherapy premedication and completed at the reduced infusion rate. |
| Grade 2-Moderate  Indicates interruption of tislelizumab therapy, but responds rapidly to symptomatic treatment (eg, antihistamines, NSAIDs, anesthetics, IV fluids); indicates prophylactic medication should be given for ≤ 24 hours | Stop the infusion. If the infusion-related reaction has resolved or has decreased in severity to Grade 1, the infusion may be resumed at 50% of the previous rate. All exacerbations should be monitored closely. Appropriate medical management should be planned as described below. Subsequent infusions should be given after the chemotherapy premedication and completed at the reduced infusion rate. |
| Grade 3 - Severe  Prolonged (e.g., not responding quickly to symptomatic medications and/or with brief interruptions of infusion); recurrence of symptoms after initial improvement; clinical sequelae indicating hospitalization | Stop the infusion immediately. Appropriate medical management should be planned as described below. Study drug treatment should be withheld in patients. |
| Grade 4 - Life-threatening consequences; urgent medical attention is indicated. | Stop the infusion immediately. Appropriate medical management should be planned as described below. Study drug therapy should be withheld in the patient. Hospitalization is recommended |

If the tislelizumab infusion rate is decreased by 50% or interrupted due to an infusion-related reaction, the decreased rate must be maintained for all subsequent infusions and premedication with chemotherapy should be administered. If a patient experiences a second infusion-related reaction (≥ Grade 2) at a slower infusion rate, the infusion should be stopped and the patient should discontinue tislelizumab therapy.

NCI-CTCAE Grade 1 or 2 Infusion Reactions: Appropriate medical management measures should be instituted based on the type of reaction. These include, but are not limited to, antihistamines (e.g., diphenhydramine or equivalent), antipyretics (e.g., acetaminophen or equivalent), and if indicated, oral or IV glucocorticoids, epinephrine, bronchodilators, and oxygen. During the next cycle, patients should receive oral antihistamines (e.g., diphenhydramine or equivalent) and antipyretics (e.g., acetaminophen or equivalent) as premedication with chemotherapy and should be closely monitored for clinical signs and symptoms of infusion reactions.

NCI-CTCAE Grade 3 or 4 Infusion Reactions: Appropriate medical management measures should be instituted immediately based on the type and severity of the reaction. These include, but are not limited to, oral or IV antihistamines, antipyretics, glucocorticoids, epinephrine, bronchodilators, and oxygen.

**Severe allergic reactions and flu-like symptoms**

If a systemic anaphylactic/anaphylactic-like reaction occurs (typically occurring within minutes of drug/antigen administration and characterized by: respiratory distress; laryngeal edema; and/or intense bronchospasm; and usually followed by vascular collapse or shock with resolution of the preceding dyspnea; skin manifestations such as pruritus and urticaria with or without edema; and gastrointestinal manifestations such as nausea, vomiting, crampy abdominal pain, and diarrhea), the infusion must be stopped immediately and the patient removed from the study. If an anaphylactic reaction is observed, the patient will receive an epinephrine injection and a dexamethasone infusion, followed by immediate monitoring of the patient and notification to the intensive care unit, who may need to be transferred if necessary.

To prevent flu-like symptoms, 25 mg of indomethacin or equivalent doses of nonsteroidal anti-inflammatory drugs (i.e., 600 mg of ibuprofen, 500 mg of naproxen sodium) may be administered 2 hours before and 8 hours after each study drug infusion. Alternative treatment for fever (i.e., paracetamol) may be given to patients at the discretion of the investigator.

# **9. Testing and statistical items**

Study evaluations are detailed in Appendix 1. Patients will be closely monitored for safety and tolerability throughout the study. All evaluations of each patient should be performed and recorded in the medical record. Drugs may be administered only after review of clinical evaluations and local laboratory test values ​​(which must be obtained before each dose) and in accordance with the study protocol guidelines, and when the conditions for administration are met.

## 9.1 Screening period

### 9.1.1 Test items to be completed within 2 weeks before enrollment

1) Demographic information, including age, gender and ethnicity;

2) Tumor history, including tumor treatment history (radiotherapy, chemotherapy, surgery), tumor metastasis, etc.;

3) Concomitant diseases and concomitant treatments;

4) Tumor markers, including squamous cell antigen (SCC), carcinoembryonic antigen (CEA), carbohydrate antigen 125 (CA125), carbohydrate antigen 19-9 (CA19-9) and carbohydrate antigen 242 (CA242).

5) Tumor evaluation, including tumor-related symptoms, imaging examinations such as chest, abdomen and pelvic CT, bone ECT (when bone metastasis is suspected), head CT or MRI (when brain metastasis is suspected).

### 9.1.2 Test items to be completed within 1 week before enrollment

1) Physical examination, with particular attention to the description of peripheral lymph nodes and palpable masses;

2) Vital signs (temperature, respiration, blood pressure and heart rate);

3) ECOG score;

4) Routine blood test;

5) Routine urine test;

6) Complete serum biochemistry (including liver and kidney function and electrolyte tests);

7) Serum virology test;

8) Blood pregnancy (if applicable);

9) Thyroid function;

10) Immunological indicators;

11) Electrocardiogram;

## 9.2 Treatment period

### 9.2.1 Clinical observation indicators during medication

During each medication period, before, during and at least 1 hour after infusion, the subjects' vital signs (temperature, respiration, blood pressure and heart rate) as well as complexion, sweating or headache, etc. must be closely monitored. The following are the items that the subjects must check every 2 weeks and when they exit the trial:

1) Physical examination, especially the description of surrounding lymph nodes and palpable masses;

2) Vital signs (temperature, respiration, blood pressure and heart rate). 9.2.2 Intraoperative and postoperative evaluation (postoperative follow-up/end of surgical treatment visit (EOT)

For all subjects who underwent surgery, the best pathological response rate was evaluated during surgery, and the ORR was evaluated according to the RECIST 1.1 standard.

### 9.2.2 Follow-up period

Safety follow-up:

Safety follow-up was conducted 30 days (±7 days) after the last dose. Including evaluation of adverse events, laboratory tests, physical examinations, ECOG scores, ECG, etc. If the study treatment was terminated for reasons other than disease progression, it is recommended to continue tumor imaging evaluation at this visit.

For immune-related AEs (irAEs) occurring within 30 days after the last treatment, follow-up should be performed as far as possible until 90 (+/-7) days after the last treatment or before the start of other anti-tumor treatment (whichever occurs first). If the study treatment was terminated for reasons other than disease progression, it is recommended to continue tumor imaging evaluation at this visit.

Survival follow-up:

Complete study treatment or early Subjects who discontinue treatment will enter the survival follow-up period from the time of disease progression or the last medication. If the study treatment is terminated for reasons other than disease progression, the corresponding assessment must continue.

The researcher conducts survival assessment, subsequent anti-tumor treatment and collects serious adverse events related to the study drug every 12 weeks (±7 days). Imaging assessment is performed every 12 weeks (±7 days) within 1 year after the end of treatment, and every 24 weeks in the second year. The survival status of the subject should be obtained through various channels such as contacting the subject, checking the subject's hospitalization records, and checking public registration records. If the subject withdraws the informed consent, the researcher can obtain the subject's survival status through public information without violating the law. In order to obtain complete survival data, the subject must still be contacted within 1 week after the survival analysis data cutoff date. Survival follow-up clinical evaluation indicators include:

• Survival assessment;

• Subsequent anti-tumor treatment;

• Collection of serious adverse events related to the study drug.

# **10. Statistical considerations**

## 10.1 Analysis set/analysis population

This study will involve the following analysis sets or analysis populations:

• Full Analysis Set (FAS)

All patients who received the study drug and had at least one post-dose efficacy follow-up data were included in the full analysis set. FAS is the primary analysis set for efficacy endpoints other than the primary efficacy endpoint pCR.

• Per-Protocol Set (PPS)

It is a subset of FAS, defined as all cases that complied with the study protocol, had good compliance, did not take prohibited drugs during the study, and completed the content specified in the case report form. No imputation was performed for missing data.

PPS is a secondary analysis set for efficacy endpoints other than the primary efficacy endpoint pCR.

• Evaluable Set (ES)

It is a subset of FAS, defined as subjects in FAS who had at least one post-baseline efficacy evaluation. ES is the primary analysis set for the primary efficacy endpoint pCR.

• Safety Set (SS)

Defined as all enrolled cases, all patients who have used the study drug at least once and have safety records after taking the drug, belong to the safety analysis set. SS is the main analysis set for safety analysis.

## 10.2 Efficacy analysis

The analysis of the primary efficacy endpoint will be based on the primary analysis set ES, and the analysis of the secondary efficacy endpoint will be repeated based on FAS and PPS.

The primary study endpoint pathological complete response rate (pCR) will be evaluated by the investigator based on pathological results. Imaging evaluation is based on RECIST 1.1 (for all lesions except bone lesions) or PCWG3 (for bone lesions) standards. Other efficacy analyses include: ORR

## 10.3 Safety analysis

Safety analysis will be based on the safety set (actual medication group). According to the reporting standards, safety analysis is limited to descriptive statistical summary, including but not limited to the following aspects, and the specific analysis will be described in the statistical analysis plan:

• Subject distribution and analysis population;

• Basic characteristics of subjects (including socio-demographic information, life history, past medical history and medication history);

• Termination of the trial by subjects;

• Summary of adverse events (all-cause and treatment-related);

• Incidence and severity of adverse events (all-cause and treatment-related);

• Summary of details of serious adverse events;

• Analysis of correlation between adverse events;

• Abnormal occurrence of laboratory indicators, vital signs, and electrocardiogram data

## 10.4 Sample size

A total of 30 subjects are planned to be enrolled.

According to literature data combined with clinical experience, the best pathological response rate of patients with locally advanced cervical cancer after neoadjuvant treatment with paclitaxel plus cisplatin is about 20%, and it is expected that the ORR can be increased to 45% after combined with PD-1 antibody. Simon’s two-stage method design is adopted, with one-sided α=0.025, β=0.2, and the required sample size is 30 cases). It is planned to enroll 10 patients in the first stage. If 3 patients achieve the best pathological response rate, they can enter the second stage and enroll another 20 patients, for a total of 30 patients.

**Appendix 1. Evaluation Schedule**

|  | Filter | Treatment period (with each period lasting 21 days) | | | | Drug safety follow-up | Surgery | | Postoperative follow-up | | | | |
| --- | --- | --- | --- | --- | --- | --- | --- | --- | --- | --- | --- | --- | --- |
|  |  | First cycle | Second cycle | The third period |  |  | Preoperative assessment | root canal treatment | 1 week | 1 month | 3 months (EOT) | 6, 12, 18, 24 months | 9, 15, 21 months |
| Days  window period | -14 to -1 | 1 | -3 to +7 | -3 to +7 |  | 28 days after the last dose (-3 to +7) | -7 to 0 | 28-42 hours after the last dose | ±3 | ±7 | ±14 | ±14 | ±14 |
| Informed consent. | X |  |  |  |  |  |  |  |  |  |  |  |  |
| Demographic data  Treatment history B | X |  |  |  |  |  |  |  |  |  |  |  |  |
| Physical examination/vital signs | X | X | X | X |  | X | X |  | X | X | X | X |  |
| ECOG evaluation | X | X | X | X |  | X | X |  | X | X | X | X |  |
| Surgical suitability assessment | X |  |  |  |  |  | X |  |  |  |  |  |  |
| Echocardiogram | X |  |  |  |  |  | X |  | X |  |  |  |  |
| Electrocardiogram | X | X | X | X |  | X | X |  | X | X | X |  |  |
| Hematology | X | X | X | X |  | X | X |  | X | X | X | X |  |
| Serum biochemistry | X | X | X | X |  | X | X |  | X | X | X | X |  |
| e | X | X | X | X |  | X | X |  | X | X | X | X |  |
| feces | X |  |  | X |  |  | X |  |  |  |  |  |  |
| coagulation | X |  | X |  |  | X | X |  | X |  |  |  |  |
| Pregnancy test | X |  |  |  |  | X | X |  |  |  |  |  |  |
| Hepatitis B two pairs of semi-quantitative g | X |  |  |  |  |  | X |  |  |  |  |  |  |
| Hepatitis C qualitative EIA | X |  |  |  |  |  | X |  |  |  |  |  |  |
| HIV | X |  |  |  |  |  | X |  |  |  |  |  |  |
| Thyroid function | X |  |  |  |  |  | X |  |  |  |  |  |  |
| lung function | X |  |  |  |  |  | X |  |  |  |  |  |  |
| Blood type |  |  |  |  |  |  | X |  |  |  |  |  |  |
| Chest CT plain scan. | X |  |  | X |  | X | X |  |  |  | X | X |  |
| Full abdomen CT scan. | X |  |  | X |  | X | X |  |  |  | X | X |  |
| Uterus  MRI with multiple parameters | X |  |  | X |  |  | X |  |  |  |  |  |  |
| past/merged | X | X | X | X |  | X | X | X | X | X | X |  |  |
| Adverse event k | X | X | X | X |  | X | X | X | X | X | X |  |  |
| Administering rituximab. |  | X | X | X |  |  |  |  |  |  |  |  |  |
| Cisplatin/carboplatin + paclitaxel chemotherapy administration |  | X | X | X |  |  |  |  |  |  |  |  |  |
| Root canal treatment and pathological assessment. |  |  |  |  |  |  |  | X |  |  |  |  |  |
| blood, sample |  | X |  | X |  |  | X |  |  |  |  |  |  |
| Archive/fresh tumor tissue. | X |  |  |  |  |  |  | X |  |  |  |  |  |
| Survival status | X | X | X | X |  | X | X | X | X | X | X | X | X |

Abbreviations: ECOG: Eastern Cooperative Oncology Group; CT: Computed Tomography; MRI: Magnetic Resonance Imaging; RECIST: Response Evaluation Criteria in Solid Tumors; v: version; CTCAE: Common Terminology Criteria for Adverse Events; EOT: End of Treatment visit.

1. Before conducting any specific examination or operation, written informed consent must be obtained. Treatment tests or examination results conducted before informed consent and within 28 days prior to enrollment can be used for screening evaluation, and do not need to be repeated unless otherwise indicated.
2. Include date of birth (or age), gender, and race/ethnicity; history of initial diagnosis and treatment, including past medication treatments, local treatments, and surgical treatments. Relevant information from imaging studies conducted before enrollment in the study can be collected for review by the researchers.
3. Vital signs collected during the research process include body temperature, pulse, and blood pressure (systolic and diastolic) measured after the patient has rested for 10 minutes. Vital signs of patients need to be recorded within 1 hour before the first two infusions of trelagliptin monoclonal antibody, during the infusions, and within 1 hour after completion. For subsequent infusions, vital signs will be collected within 60 minutes before the infusion, and if indicated clinically, during and 30 minutes after the infusion. Researchers should inquire about changes in visual acuity, visual disturbances, or eye inflammation during each planned study visit during trelagliptin monoclonal antibody treatment. If there are any changes in vision, patients should be referred to an appropriate specialist for further management guidance.
4. The patient should rest calmly for at least 10 minutes before undergoing an electrocardiogram examination.
5. The local laboratory will evaluate hematology (complete blood count and coagulation profile), serum biochemistry (including cardiac, liver, and kidney function tests), and feces. After the first cycle, results should be reviewed within 24 hours before each dose of the investigational drug. If there are clinical indications during the follow-up period, stool samples need to be collected. During routine assessments, if urine protein is ≥2+ on the test strip, a 24-hour urine sample should be taken to measure total protein, followed by a random urine sample for total protein and creatinine, to determine the protein-to-creatinine ratio.
6. Conduct pregnancy tests on all women of childbearing age. If the pregnancy test shows a faint positive result, a serum pregnancy test must be conducted for confirmation.
7. Serological tests including HBV/HCV/HIV (HBsAg, Anti-HCV, Anti-HIV) will be conducted at the local laboratory. If HBsAg is positive, an HBV DNA test is required. If Anti-HCV is positive, an HCV RNA test is needed.
8. In addition to screening and preoperative evaluation, if there are clinical indications, the researcher will determine whether thyroid function testing is needed.
9. Pulmonary function tests include but are not limited to spirometry and diffusion capacity assessment.

**Appendix 2. ECOG Performance Status**

| **Level** | **Description** |
| --- | --- |
| 0 | Be free to engage in all activities prior to illness without any restrictions. |
| 1 | Limited to moderate physical activities, but able to walk freely and engage in light physical or sedentary work, including typical household chores or office work. |
| 2 | Able to move freely and take care of oneself; but has lost the ability to work; can be out of bed and active for at least half of the day |
| 3 | Life can only partially take care of itself, spending more than half of the day in bed or a wheelchair. |
| 4 | completely disabled; unable to care for oneself; bedridden or unable to get out of a wheelchair |
| 5 | death |

Source: Oken MM, Creech RH, Tormey DC, et al. Toxicity and Response Criteria of the Eastern Cooperative Oncology Group. Am J Clin Oncol. 1982;5(6):649-55.

**Appendix 3. New York Heart Association Functional Classification**

| **Level** | **Description** |
| --- | --- |
| I | Physical activities are not limited. No fatigue, palpitations, or shortness of breath during daily activities. |
| II | Mild restriction in physical activity. No symptoms at rest, but fatigue, palpitations, and breathlessness (shortness of breath) may occur with daily activities. |
| III | Physical activities are significantly limited. There are no symptoms at rest, but mild activities can lead to fatigue, palpitations, or difficulty breathing. |
| IV | Unable to engage in any physical activity without discomfort. Experience symptoms of heart failure even at rest. Feel discomfort after engaging in any physical activity. |

Excerpt from Dolgin M, New York Heart Association, Fox AC, Gorlin R, Levin RI, New York Heart Association. Criteria

Committee.Nomenclature and criteria for diagnosis of diseases of the heart and great vessels.9th ed. Boston, MA: Lippincott Williams and Wilkins; March 1, 1994.

Original source: Criteria Committee, New York Heart Association, Inc. Diseases of the Heart and Blood Vessels.Nomenclature and Criteria for diagnosis, 6th edition Boston, Little, Brown and Co. 1964, p 114

**Appendix 4. Past history of immunodeficiency or autoimmune diseases**

Carefully inquire about potential patients to determine if they have a history of sexual or congenital immunodeficiency or autoimmune diseases.

| Acute disseminated encephalomyelitis | Ed is sick. |
| --- | --- |
| Ankylosing spondylitis | Antiphospholipid syndrome |
| Aplastic anemia | Autoimmune hemolytic anemia |
| Autoimmune hepatitis | Autoimmune hypoparathyroidism |
| Autoimmune pituitary inflammation | Autoimmune myocarditis |
| Self-immunity | autoimmunity |
| Immune thrombocytopenic purpura | Behcet's disease |
| Pemphigus vulgaris | Chronic inflammatory demyelinating polyneuropathy |
| Churg-Strauss syndrome/allergic granulomatosis angiitis | Crohn's disease |
| Dermatomyositis | Familial dysautonomia |
| Bullous pemphigoid | Pemphigoid gestationis |
| Giant cell arteritis | Goodpasture Syndrome |
| Granulomatosis with polyangiitis | Graves' disease |
| Grinberg Syndrome | Hashimoto's disease |
| Immunoglobulin A (IgA) neuropathy | Inflammatory bowel disease |
| Interstitial inflammation | Kawasaki disease |
| Myasthenia Gravis | Lupus erythematosus |
| Lyme disease (chronic) | Corneal ulcer caused by Acanthamoeba |
| Hardened spot disease | Multiple sclerosis |
| Myasthenia gravis | Spasticity |
| Oculomotor spasm syndrome | Optic neuritis |
| Hashimoto's thyroiditis | pemphigus |
| Aplastic anemia | Rheumatoid arthritis |
| Osteoarthritis | polyglandular autoimmune syndrome |
| Primary biliary cirrhosis | Psoriasis |
| Wright's syndrome | Rheumatoid arthritis |
| Granuloma nodules | Sheehan's syndrome |
| stiff person syndrome | Takayasu's arteritis |
| Ulcerative colitis | Vogt-Koyanagi-Harada (VKH) disease |

**Appendix 5. Evaluation and Management of Immune-Related Adverse Events**

The following recommendations on the diagnosis and management of various irAEs will be used as a guide. This document should be used in conjunction with expert clinical judgment (specialists experienced in the use of immunotherapies in cancer treatment) and guidelines or policies from various institutions.

The diagnostic process for irAEs includes necessary blood tests, imaging studies, histopathological and microbiological assessments to rule out other causes such as infection, disease progression, and concurrent side effects. In addition to these test results, the following factors should also be considered when diagnosing irAEs:

• Is there a temporal relationship between the administration of Tislelizumab and adverse events?

• How do patients react after stopping treatment with Tislelizumab?

• Will the event occur again when using Tislelizumab again?

• Has there been any clinical improvement after using corticosteroids?

• Is this event a autoimmune systemic disease?

• PD or other differential diagnoses may be more reasonable explanations?

If the alternative explanation of self-immune toxicity is excluded, the irAE field relevant to AE in CRF should be checked.

**Recommended diagnostic tests for the management of potential immune-related adverse events.**

| **Immune-related toxicity.** | **Diagnostic assessment indices** |
| --- | --- |
| Thyroid disease | Arrange and repeat thyroid function tests (TSH and T4). |
| Pituitaryitis | Check the field of vision and consider the blood distribution within the pituitary axis. Patients presenting with headache, visual disturbances, unexplained fatigue, weakness, weight loss, and unexplained constitutional symptoms should undergo pituitary and full brain MRI scans. If any abnormalities are found, consider arranging a consultation with an endocrinologist. |
| Non-infectious pneumonia | Patients with new or worsening pulmonary symptoms or signs (such as upper respiratory tract infections, cough, shortness of breath, or hypoxia) should be evaluated by high-resolution CT. Consider pulmonary function testing, including DLCO. Imaging findings are usually nonspecific. Depending on the location of the lesion, bronchoscopy, bronchoalveolar lavage, or lung biopsy may be considered. If the cause is unclear, consult a pulmonologist for further evaluation. |
| Neurotoxicity | Comprehensive neurological examination and brain MRI scan should be conducted for all central nervous system symptoms; review alcohol history and other medications. Screen for diabetes, assess blood levels of B12/folate, HIV status, TFT, and consider autoimmune serology tests. Consider whether brain/spinal cord MRI/MRA scans are needed, as well as nerve conduction studies for peripheral neuropathy. If abnormalities are found, consider consulting a neurologist. |
| Colitis | Check the dietary intake and exclude fatty diarrhea. Consider a comprehensive examination including the following: FBC, UEC, LFT, CRP, TFT, stool microscopy and culture, virus PCR, C. difficile toxin, cryptosporidium (resistant organisms). If experiencing abdominal discomfort, consider imaging studies such as X-ray or CT scan. If the patient presents with severe pain or abdominal distension, consider colonoscopy and arrange for biopsies or surgical intervention as needed. |
| Eye organ disease | If a patient presents with acute, new-onset, or worsening eye inflammation, blurred vision, or other visual disturbances, please refer the patient to an ophthalmologist immediately for evaluation and treatment. |
| Hepatitis | Detect alanine aminotransferase (ALT)/aspartate aminotransferase (AST)/international normalized ratio (INR)/albumin; frequency will depend on the severity of adverse events (e.g., once daily if Grade 3 or 4, every 2-3 days if Grade 2, until resolution). Review medication history (e.g., statins, antibiotics) and alcohol consumption. Conduct liver screening, including serology for hepatitis A/B/C, hepatitis E PCR, and assess anti-ANA/SMA/LKM/SLA/LP/LCI, iron studies. Consider imaging studies, such as ultrasound for metastases or thromboembolism. Consult with a hepatologist and consider liver biopsy. |
| Pituitaryitis | Review hydration status and history. Perform examination and cultivate. Consider renal ultrasound, protein assessment (dipstick/24-hour collection), or phase contrast microscopy. - For further management assistance, consult a nephrologist. |
| Dermatology | Consider performing a physical examination for other possible causes. If a skin biopsy is needed, consider referring to a dermatologist. |
| Joint or inflammation | Check the patient's skeletal medical history and perform a bone examination. Consider joint X-rays and other imaging studies as needed to rule out metastatic disease. Conduct autoimmune serology tests and consult a rheumatologist for further management assistance if necessary. If there is suspicion of myositis/rhabdomyolysis/myasthenia gravis, include CK, ESR, CRP, and troponin, and consider a biopsy. |
| Myocarditis | Please have an electrocardiogram, echocardiogram, CK/CK-MB, and troponin (I and/or T) tests done, and consult a cardiologist. |

Acronyms: AE, Adverse Event; ALT, Alanine Aminotransferase; ANA, Antinuclear Antibody; AST, Aspartate Aminotransferase; CK, Creatine Kinase; CK-MB, Creatine Kinase-MB Isoenzyme; CNS, Central Nervous System; CRP, C-Reactive Protein; CT, Computed Tomography; DLCO, Diffusing Capacity of the Lungs for Carbon Monoxide; ECG, Electrocardiogram; ESR, Erythrocyte Sedimentation Rate; FBC, Full Blood Count; INR, International Normalized Ratio; LCI, Liver Cytosol Antibody; LFT, Liver Function Test; LKM, Liver Kidney Microsomal Antibody; LP, Liver Pancreas Antigen; MRA, Magnetic Resonance Angiography; MRI, Magnetic Resonance Imaging; PCR, Polymerase Chain Reaction; SLA, Soluble Liver Antigen; SMA, Smooth Muscle Antibody; T4, Thyroxine; TFT, Thyroid Function Test; TSH, Thyroid-Stimulating Hormone; UEC, Urea Electrolytes and Creatinine.

**Treatment of immune-related adverse events.**

• Immune-related adverse reactions can escalate rapidly; research treatment interruptions, closely monitor, timely diagnostic tests, and consider therapeutic interventions for patients.

• After immunosuppressive treatment, immunological adverse events should improve rapidly. If there is no improvement, please check the diagnosis, seek further expert advice, and contact the medical research monitor.

• For patients experiencing rapid alleviation of side effects from grade 3 toxicity, it may be considered to reintroduce the investigational drug if there is evidence indicating a clinical response of the tumor to the study treatment following consultation with the medical monitor.

• The table below shows the steroid doses for oral or intravenous (methyl)prednisolone. Other corticosteroids with equivalent doses that can be used as alternatives are considered for steroid-refractory irAEs. Non-corticosteroid immunosuppressive agents, such as mycophenolate, may be considered.

• If a patient is undergoing immunosuppressive therapy, it is advisable to consider using antibiotics to prevent opportunistic infections.

| **Self-mutation toxicity** | **Level** | **Treatment guidelines (based on clinical judgment)** | **Study drug administration.** |
| --- | --- | --- | --- |
| **Thyroid disease** | 1 or 2  Asymptomatic TFT abnormalities or mild symptoms | If hypothyroidism develops, replace thyroxine until TSH/T4 levels return to normal range. Consult an internist if patient has thyrotoxicosis. If systemic symptoms develop: Withhold study treatment, treat with beta-blockers, consider oral troparin 0.5 mg/kg/day for thyroid pain. Taper corticosteroid dose over 2-4 weeks. Monitor thyroid function to determine if replacement therapy is needed. | Continue researching on treatment, stop treatment if experiencing systemic symptoms. |
|  | 3 or 4  Severe symptoms, need hospitalization for treatment. | Transfer the patient to an internist. If it is hypothyroidism, switch to levothyroxine 0.5-1.6 g/kg/day (the recommended initial dose is 0.5 g/kg/day for elderly patients or those with complications). - Oral prednisone 0.5 mg/kg/day is used to treat thyroid pain. Patients with hyperthyroidism may need beta-blockers for treatment, and may also need propylthiouracil until the thyroiditis subsides. | Pause treatment; Resume after achieving remission/improvement to Grade 0 or 1. |
| **Pituitaryitis** | 1 or 2  Mild to moderate symptoms | If alternative treatment is needed, refer the patient to an internal medicine doctor. Patients with pituitary gland inflammation should take oral prednisone at a dose of 0.5-1 mg/kg/day. Gradually reduce the dosage of corticosteroids over at least one month. If there is no improvement within 48 hours, proceed with a level 3 or 4 intervention. | Continue to research the treatment. |
|  | 3 or 4  Symptoms that are severe or life-threatening. | If evaluation and treatment are needed, refer the patient to an internist. Treat headaches/visual disturbances caused by pituitary inflammation with 1 mg/kg methylprednisolone. Switch to oral prednisone and gradually taper the dose over at least 1 month. Maintain replacement therapy based on the internist's recommendations. | Suspend treatment of patients with headache/visual impairment caused by pituitary inflammation until relief/improvement to grade 2 or lower. Discontinuation of medication is usually not required. |
| **Non-infectious pneumonia** | 1  Radiologic changes | Monitor symptoms every 2-3 days. If the signs worsen, take action at level 2. | Consider pausing research on treatment until symptoms improve and the cause is determined. |
|  | 2  Symptom: Exertional dyspnea | If a suspected infection is present, start using antibiotics. If symptoms/signs persist for 48 hours or worsen, consider oral prednisone 1 mg/kg/day. Consider prophylaxis for Pneumocystis infection. Gradually reduce the dose of corticosteroids over at least 6 weeks. Consider monitoring for potential side effects of corticosteroids, such as blood glucose levels and supplementing with vitamin D/calcium. | Discontinue the study treatment if symptoms are completely relieved or controlled at ≤ 10 mg/day of prednisone, and treatment can be resumed if needed. Stop the study treatment if symptoms persist after corticosteroid therapy. |
|  | 3 or 4  Severe or life-threatening symptoms  Respiratory pause during a calm state. | Admission for treatment, starting intravenous infusion of methylprednisolone at 2-4 mg/kg/day. If no improvement or deterioration after 48 hours, consider infliximab at 5 mg/kg (if liver is not involved). Switch to oral prednisone, and gradually taper the dose over at least 2 months. Use prophylactic antibiotics and consider prevention of Pneumocystis jiroveci infection and other corticosteroid side effects, such as monitoring blood sugar, and vitamin D/calcium supplementation. | Discontinue research treatment. |
| **Neurotoxicity** | 1  Mild symptoms | - | Continue to research the treatment. |
| **Neurotoxicity** | 2  Moderate symptoms | Oral administration of 0.5-1 mg/kg/day of dragon for treatment. Gradually reduce the dose over at least 4 weeks. Consultation with neurology department invited. | Pause treatment; Resume after achieving remission/improvement to Grade 0 or 1. |
|  | 3 or 4  serious/life-threatening | Start oral or intravenous administration of prednisone at a dose of 1-2 mg/kg/day based on the symptoms. Gradually reduce the corticosteroid dose over a period of at least 4 weeks. If there is no improvement within 72-96 hours, consider using azathioprine, mycophenolate mofetil, or cyclosporine. | Discontinue research treatment. |
| **Colitis/diarrhea** | 1  Mild symptoms: pass liquid stools more than baseline <3 times per day, feeling well | Targeted treatment: liquid, ropivacaine, avoid high fiber/lactose diet. If Grade 1 exceeds 14 days, manage as Grade 2 event. | Continue to research the treatment. |
|  | 2  Moderate symptoms: increasing frequency of liquid stools by 4-6 times per day compared to baseline, or abdominal pain, or bloody stools, or nausea, or nocturnal attacks. | Oral administration of Den 0.5 mg/kg/day (non-enteric coated tablets). Start treatment immediately without waiting for various diagnostic test results. Gradually discontinue steroids within 2-4 weeks. If symptoms recur, consider endoscopic examination. | Pause research and treatment; resume after relief/improvement to baseline levels. |
|  | 3  Severe symptoms: passing liquid stool more than 6 times per day or needing to pass stool within 1 hour after eating | Start by switching from intravenous methylprednisolone to oral prednisone, and gradually taper the dose over at least 4 weeks. Consider measures to prevent corticosteroid side effects, such as monitoring blood glucose levels and providing vitamin supplementation. If there is no improvement within 72 hours or symptoms worsen, consult with a healthcare professional. | Pause research treatment; consider resuming treatment once symptoms have been relieved/improved to baseline levels and discussed with the study medical monitor. |
|  | 4  Life-threatening symptoms | Consider using Infliximab 5 mg/kg if there are no perforation, sepsis, tuberculosis (TB), hepatitis, NYHA III/IV CHF. Otherwise, consider other immunosuppressive agents: MMF or Tacrolimus. Please consult a gastroenterologist for colonoscopy/sigmoidoscopy examination. | Discontinue research treatment. |
| **Skin reaction** | 1  Rash, with or without any symptoms, is <10% of body surface area (BSA). | Avoid exposure to sunlight on the skin; it is recommended to use topical moisturizers. | Continue to research the treatment. |
|  | 2  Rash covering 10%-30% of the body surface area | Avoid exposure to skin and sunlight; suggest using moisturizers topically. Topical corticosteroids (moderate potency cream once daily, or potent potency cream twice daily) ± oral or topical antihistamines for treating itching. Consider short-term oral corticosteroids. | Continue to research the treatment. |
|  | 3  Rash covering > 30% of body surface area or 2nd degree, accompanied by significant symptoms. | Avoid skin exposure to sunlight; consider using topical moisturizers. Based on clinical judgment, decide whether to start using moderate symptoms: oral prednisone 0.5-1mg/kg/day for 3 days, then gradually taper off over 2-4 weeks. For severe symptoms: intravenous methylprednisolone 0.5-1 mg/kg/day; switch to oral prednisone, and taper off over at least 4 weeks. | Pause the research treatment and discuss with the medical monitor. Treatment can be resumed if the AE has resolved or improved to mild rash (Grade 1 or 2). |
|  | 4  Skin necrosis involving >30% body surface area, accompanied by related symptoms such as erythema, purpura, and epidermal shedding. | Start intravenous injection of methylprednisolone at 1-2mg/kg/day. Switch to oral prednisone and gradually decrease the dose over at least 4 weeks. Admit to the hospital for treatment and urgently request a dermatology consultation. | Discontinue research treatment. |
| **Hepatitis** | 1  ALT or AST>ULN up to 3×ULN | Repeat LFT within 1 week, then repeat before next dose to confirm no worsening. If LFT worsens, repeat every 48-72 hours until improvement is observed. | If LFT remains stable or improves, continue with the treatment. If LFT worsens, then pause the treatment and wait for an improvement to be observed. |
|  | 2  ALT or AST 3-5 times ULN | Review LFT every 48-72 hours. If there is an increase in ALT/AST levels due to hepatitis, consider oral prednisolone at a dose of 0.5-1 mg/kg/day for 3 days, then gradually taper off over 2-4 weeks. If ALT/AST levels are elevated due to other causes, start oral prednisolone at 1 mg/kg/day and taper off gradually over 2-4 weeks. If LFT worsens, adjust the dose based on clinical judgment. | Pause the treatment; improve to baseline level, then treatment can resume gradually reducing the dosage to ≤10 mg. |
|  | 3  ALT or AST >5-20×ULN | ALT/AST < 400 IU/L and bilirubin/INR/albumin normal: start oral prednisolone at 1 mg/kg, gradually tapering the dose over at least 4 weeks. ALT/AST > 400 IU/L or increased bilirubin/INR/albumin: start intravenous (methyl)prednisolone at 2 mg/kg/day. When LFT improves to grade 2 or lower, switch to oral prednisolone, gradually tapering the dose over at least 4 weeks. | Pause research treatment until the cause is identified. If there are suspicions about the investigational drug, discontinue research treatment. |
|  | 4  ALT or AST > 20 × ULN | Start intravenous injection of methylprednisolone at a dose of 2mg/kg/day. Switch to oral prednisone and gradually reduce the dose over at least 6 weeks. | Stop researching treatment. |
|  | Despite the use of steroids, LFTs are still deteriorating.  • If oral dragon, then change to intravenous pulse methylprednisolone.  • If injected intravenously, MMF, 500-1000 mg, twice daily.  • If MMF deteriorates, consider switching to Tacrolimus.  The required steroid type, duration, and dosage will depend on the severity of the condition. | | |
| **nephritis** | 1  Creatinine 1.5 times baseline or > ULN or 1.5 times ULN | Review creatinine levels weekly. If symptoms worsen, follow the steps below. | Continue to research the treatment. |
|  | 2  Creatinine >1.5 times to 3 times baseline or >1.5 times to 3 times the upper limit of normal | Ensure hydration and recheck creatinine levels within 48-72 hours; if no improvement, consider measuring creatinine clearance over 24 hours. Discuss with a nephrologist the need for a renal biopsy. If drug-induced, initiate oral prednisolone at 0.5-1 mg/kg and taper gradually over at least 2 weeks. Recheck creatinine/U&E every 48-72 hours. | Pause the treatment if it is not due to drug toxicity, and restart the treatment. If it is due to the investigational drug and has been relieved/improved to baseline level: restart the use of the investigational drug (titrate down to <10). |
|  | 3  Creatinine >3 times baseline or >3-6 times ULN. | Arrange for the patient to be hospitalized for monitoring and restoration of fluid balance; check creatinine levels every 24 hours; consult a nephrologist for treatment guidance (based on clinical judgment) and discuss the need for renal biopsy if necessary. If the condition worsens, start intravenous injection of methylprednisolone 1-2 mg/kg. Gradually taper the dose of corticosteroids over at least 4 weeks. | Pause research treatment until the cause is identified. If there are suspicions about the investigational drug, discontinue research treatment. |
|  | 4  Creatinine >6×ULN | Patients should receive treatment in hospitals where renal replacement therapy is available. | Stop researching treatment. |
| **Diabetes/hyperglycemia** | 1  Fasting blood glucose level reaches 160 mg/dL; ULN to 8.9 mmol/L. | Monitor closely and manage according to local guidelines. Consider checking C-peptide, glutamic acid decarboxylase antibodies, and islet cell antibodies. | Continue to research the treatment. |
|  | 2  Fasting blood glucose levels reaching 160-250 mg/dL; 8.9-13.9 mmol/L ULN | Review blood sugar levels at least once a week. Manage according to local guidelines. | Continue research treatment, suspend treatment if hyperglycemia worsens. Resume treatment if blood sugar stabilizes at baseline or grade 0 or 1. |
|  | 3  Fasting blood glucose levels reaching 250-500 mg/dL; 13.9-27.8 mmol/L. | Admit the patient to the hospital and refer to a diabetes specialist for management of high blood sugar. Corticosteroids can worsen hyperglycemia and should be avoided. | Pause the research treatment until the symptoms of hyperglycemia in the patient subside and the blood sugar stabilizes at baseline or at grade 0 or 1. |
|  | 4  Fasting blood glucose value reaches >500 mg/dL; >27.8 mmol/L. | Arrange for the patient to be hospitalized and establish local diabetes emergency management measures. Transfer the patient to a diabetes specialist for insulin maintenance and monitoring. |  |
| **Eye Toxicity** | 1  Asymptomatic, eye examination/detection abnormalities | Consider other reasons and prescribe local treatment as needed. | Continue to research the treatment. |
|  | 2  Anterior uveitis or mild symptoms | If assessment and local treatment with corticosteroids is needed, refer the patient to an ophthalmologist. Consider using oral corticosteroids for a period of time. | Continue the research on treatment. If symptoms worsen or if visual impairment symptoms arise, stop treatment. |
|  | 3  Endophthalmitis or panophthalmitis presenting with obvious symptoms | Urgently transfer the patient to an ophthalmologist. Start with oral prednisone 1-2 mg/kg and gradually taper the dose over at least 4 weeks. | Pause treatment research until improvement to Grade 0 or 1; resumption only after discussion with the research medical monitor. |
|  | 4  Impaired eye with blindness (at least 20/200) | Start intravenous injection of (methyl)prednisolone at 2mg/kg/day. Switch to oral prednisolone and gradually decrease the dose over a minimum of 4 weeks. | Stop researching treatment. |
| **Pancreatitis** | 2  Asymptomatic, abnormal blood test. | Test pancreatic enzymes. | Continue to research the treatment. |
|  | 3  Abdominal pain, nausea, and vomiting | Arrange for hospitalization to receive emergency treatment. Start intravenous infusion of (methyl)prednisolone at 1-2 mg/kg/day. If amylase/lipase levels improve to grade 2, switch to oral prednisolone and taper off over a minimum of 4 weeks. | Pause research and treatment; must consult with the medical research supervisor before resuming. |
|  | 4  Acute abdominal pain, emergency surgery | Arrange for hospital admission for emergency treatment and make appropriate referrals. | Discontinue research treatment. |
| **Arthritis** | 1  Mild pain with inflammation and swelling. | Follow the local guidelines. | Continue to research the treatment. |
|  | 2  Moderate pain accompanied by inflammation and swelling, limited range of motion due to the use of instruments (fine motor skills). | Manage according to local guidelines. Consider referring the patient to a rheumatologist. If symptoms worsen during treatment, treat as a level 3 event. | Continue treatment; if symptoms worsen, pause treatment and resume once symptoms improve to baseline or grade 0 or 1. |
|  | 3  Pain accompanying inflammatory joint injury, daily activities symptoms or permanent restriction | Transfer the patient to a rheumatologist for evaluation and treatment. Start with oral prednisone at a dose of 0.5-1mg/kg, and gradually taper the dose over at least 4 weeks. | Pause the research treatment until the condition improves to grade 0 or 1; must be resumed only after discussion with the medical research supervisor. |
| **mucositis/stomatitis** | 1  Just test results or very few symptoms. | Consider local treatment or pain relief according to local guidelines. | Continue to research the treatment. |
|  | 2  Moderate pain, taken orally, limited activity using instruments | Manage according to local guidelines. Consider transferring the patient to a rheumatologist. If symptoms worsen during treatment, escalate as a level 3 event. | Continue to research the treatment. |
|  | 3. Pain, limited food and liquid intake, limited daily activities. | Arrange for hospitalization to receive appropriate treatment. Start intravenous injection of (methyl) prednisolone 1-2 mg/kg. If the symptoms improve to Grade 2, switch to oral dexamethasone and gradually stop taking it over at least 4 weeks. | Pause treatment until improvement to Level 0 or 1. |
|  | 4. Life-threatening complications or dehydration. | Arrange hospital admission for emergency care. Consider intravenous injection of corticosteroids if there are no contraindications to infection. | Stop researching treatment. |
| **Myositis/rhabdomyolysis** | 1  Mild weakness with/without pain | Start analgesics. If CK levels are significantly elevated and the patient is symptomatic, consider oral steroids as second-line treatment. | Continue to research the treatment. |
|  | 2. Mild weakness with/without pain | If the CK level reaches 3 times the ULN or higher, start with oral prednisone 0.5-1mg/kg and gradually reduce the dose over at least 4 weeks. | Pause research treatment; until improve to level 0 or 1 |
|  | 3 or 4. Severe weakness, self-limiting | Arrange hospitalization and start oral prednisone at 1 mg/kg. Consider intravenous methylprednisolone at 1-2 mg/kg/day as maintenance therapy for severe activity limitation or swallowing difficulty. If symptoms do not improve, immunosuppressive therapy. Gradually reduce the dose of oral steroids over at least 4 weeks. | Pause treatment until improvement to grade 0 or 1 is achieved. Discontinue treatment if evidence of myocardial involvement is present. |
| **Myocarditis** | ＜2. Elevated CK-MB or significantly elevated troponin levels in the absence of symptoms, or clinically significant ventricular conduction delay. | Initiate cardiac evaluation with close monitoring through repeated serum testing; consider referral to cardiology specialist. If diagnosis of myocarditis is confirmed, classify as Grade 2. | Pause the treatment research. |
|  | 2  Symptoms after light or moderate physical activity | Admit the patient and start oral or IV methylprednisolone at a dose of 1-2 mg/kg per day. Consult a cardiologist and manage heart failure symptoms according to local guidelines. If symptoms do not immediately improve, switch to pulse dose methylprednisolone 1 g per day, along with MMF, eculizumab, or anti-thymocyte globulin. | If the diagnosis of myocarditis is confirmed, research treatment for the patient should be permanently discontinued. Patients with no symptoms or with mild symptoms should not resume treatment with tocilizumab if heart parameters have not returned to baseline levels and discussions have taken place with the medical monitor. |
|  | 3  Severe symptoms after light physical activity. |  |  |
|  | 4  Life-threatening |  |  |

Abbreviations: AE, adverse event; ALT, alanine aminotransferase; AST, aspartate aminotransferase; BSA, body surface area; CHF, congestive heart failure; CK, creatine kinase; CK-MB, creatine kinase-MB; INR, international normalized ratio; IV, intravenous injection; LFT, liver function test; MMF, mycophenolate mofetil; NYHA, New York Heart Association; T4, thyroxine; TB, tuberculosis; TFT, thyroid function test; TSH, thyroid-stimulating hormone; U&E, urea and electrolytes; ULN, upper limit of normal.

**Appendix 6. Response Evaluation Criteria in Solid Tumors (RECIST) Guidelines, Version 1.1**

The following text is excerpted from the following reference:

Eisenhauer EA, Therasse P, Bogaerts J, et al.New response evaluation criteria in solid tumours: revised RECIST guideline (version 1.1).Eur J Cancer.2009;45(2):228-47.

**Definition**

Assessment of response and progression in this study will be conducted based on the international criteria proposed by the RECIST (version 1.1). RECIST only utilizes changes in the longest diameter of tumor lesions (unidimensional measurement).

Note: As indicated in the following, lesions will be categorized as measurable or non-measurable. The term "evaluable" used for reference to measurability will no longer be employed as it does not contribute any additional meaning or accuracy.

Measurable diseases.

Tumor lesion: At least one dimension not less than the lower limit (instrument detection) must be accurately measured (the longest diameter on the measuring instrument will be recorded).

• 10 mm, using CT scanning (regardless of scanner type) and MRI (at least twice the slice thickness, minimum 10 millimeters)

• 10 mm, measured with calipers in a clinical examination (lesion located on the surface of the body)

• 20 mm, radiograph of the chest (if borders are clear and surrounded by inflated lung fields).

Malignant: When evaluating with a CT scan (CT scan slice thickness should not exceed 5 millimeters), the short-axis must be ≥15mm to be considered pathologically enlarged and measurable. Only the short-axis length should be measured and tracked at baseline and follow-up.

Non-measurable disease

All other lesions (or disease sites), including small lesions (≥10 to <15 mm in longest diameter by conventional techniques or <10 mm by CT scan), are considered non-measurable disease. Leptomeningeal disease, ascites, pleural or spiral pericardial effusions, inflammatory breast disease, skin or lung involvement, and abdominal masses/abdominal organomegaly that are determined by physical examination and cannot be measured by reproducible imaging techniques are considered non-measurable disease.Bone disease:

• Bone scan, PET scan, or X-ray are not considered appropriate imaging techniques for measuring bone lesions. However, these techniques can be used to confirm the presence or disappearance of bone lesions.

• If the soft tissue components meet the above definitional criteria and dissolve bone lesions or dissolution-acute mixed lesions with recognizable soft tissues can be evaluated through cross-sectional imaging techniques such as CT or MRI, they can be considered measurable lesions.

• Acute bone disease is immeasurable.

Cystic lesion:

• A simple cyst lesion that meets the criteria for a simple cyst on imaging should not be considered malignant (regardless of measurability) because by definition they are simple cysts.

• Believe that 'cystic lesions' which are considered cystic metastasis, if they meet the above measurable definition, can be regarded as measurable."

Evaluate the lesions. However, if a patient has both cystic and non-cystic lesions at the same time, it is preferable to consider all of these as target lesions.

Lesions already treated locally:

• Tumor lesions previously located in the irradiation field or in other locally treated areas are generally not considered measurable diseases unless it is demonstrated that the lesions are still present. The study protocol should specify under what conditions such lesions can be considered measurable diseases.

Target lesion:

All measurable lesions (up to 2 per organ, a total of 5 lesions) should be designated as target lesions, recorded, and measured at baseline. Target lesions should be selected based on their size (longest diameter) and may represent all involved organs. Furthermore, target lesions should aid in reproducibility of measurements.

It is worth mentioning that these are normal anatomical structures that can be seen on imaging studies even in the absence of tumors. Pathological nodules defined as measurable and identifiable as target lesions must have a short-axis ≥15 mm on CT scans. The baseline sum is calculated by adding up the short-axis of only these nodules. The short-axis of the nodules is the diameter radiologists typically use to assess whether the nodules are involved with solid tumors. Nodule size is usually reported as two-dimensional data in the image plane acquired (for CT scans, the result is almost always in the axial plane; for MRI, the acquisition plane can be axial, sagittal, or coronal). The smaller of these measurements is the short-axis. For example, a abdominal nodule reported as 20 mm × 30 mm has a short-axis of 20 mm and is characterized as a malignant, measurable nodule. In this instance, 20 mm should be recorded as the nodule measurement value. All other pathological nodules (short-axis ≥10 mm but <15 mm) should be considered non-target lesions. Nodules with a short-axis <10 mm are considered non-pathological nodules and should not be recorded or tracked.

Calculate the sum of the diameters of all target lesions (for non-nodal lesions, use the longest diameter, for nodal lesions, use the short axis), and report it as the sum of baseline diameters. If the sum includes the short axis as described above, include only the short axis in the sum. The sum of baseline diameters will be used as a reference for further characterizing objective tumor response in various measurable dimensions of the disease.

Non-target Lesion:

All other lesions (or disease sites) including pathological findings should be identified as non-target lesions and should be documented at baseline. - No measurements are required, these lesions should be recorded as "present", "absent" or in few cases "unequivocal progression" (see more details below). In addition, multiple non-target lesions involving the same organ can be documented on the case report form (e.g., "multiple enlargements" or "multiple liver metastases").

Measurable Disease Assessment Guidelines:

All measurements should be recorded in metric units. For clinical assessments, a caliper should be used for measurements. All baseline assessments must be conducted as close to the start of treatment as possible, and no later than 4 weeks before the start of treatment.

When characterizing each identified and reported lesion, the same assessment and techniques should be used at baseline and at follow-up. Except for lesions that can only be assessed by clinical examination and are not amenable to imaging detection, all lesions must be evaluated using imaging detection, and clinical examination alone is not sufficient.

Clinical manifestations:

Only superficial lesions below 10mm, such as skin nodules, should be considered for clinical assessment using a caliper. For skin lesions, it is recommended to record with colored photos, attaching a scale for measuring lesion size. As mentioned earlier, when a lesion can be assessed clinically and through imaging studies, imaging studies should be conducted because they are more objective. Furthermore, post-treatment imaging can be used for retrospective evaluation of study endpoints.

• Chest X-ray: For measuring lesions in the chest, chest CT scan is preferred over chest X-ray, especially when there is progression at important treatment endpoints, as CT is more sensitive than X-ray, especially in detecting new lesions. Of course, lesions with clear borders and surrounded by inflated lungs can also be detected using chest X-ray.

• CT, MRI: CT is currently the most effective and reproducible method for evaluating the therapeutic efficacy of lesions. This guideline specifies whether CT scanning can be used to measure lesions based on whether the CT slice thickness does not exceed 5mm. When the CT scan slice thickness exceeds 5 mm, the minimum measurable lesion size should be twice the slice thickness. MRI can also be used in some cases, such as for whole-body scans.

• Ultrasound examination: Ultrasound examination is not suitable for assessing the size of lesions and should not be used for measurements. Ultrasound examination cannot completely reproduce between two successive observations, and the results depend on the operator from one examination to the next.

It cannot be guaranteed that using the same technique will yield the same measurement results. If new lesions are discovered during the study using ultrasound, it is recommended to verify with CT or MRI. If concerned about radiation exposure from CT, MRI can be used as an alternative in some cases.

• Endoscopy, laparoscopy: It is not recommended to assess solid tumors using these techniques. However, these examinations can be used when confirming complete pathological remission with biopsy or determining whether there is recurrence after achieving CR or surgical resection.

• Tumor Markers: Tumor markers cannot be used alone to evaluate the therapeutic effect of solid tumors. If the marker level is above the normal upper limit at baseline, it must return to that level when the patient is judged to be in complete remission (CR). Because tumor markers have disease specificity, the assay methodology should be noted in protocols specific to a particular disease.

Special guidelines on changes in CA-125 levels (recurrence of cancer) and PSA levels (recurrence) have been published. In addition, the International Gynecologic Oncology Group has developed advancements in CA-125, which will be combined with objective tumor assessment for frontline cancer research.

• Cytology, histology: These techniques can be used to differentiate between PR and CR in individual cases as required by the protocol (e.g., residual tumor in tumors, such as in germ cell tumors where known residual benign tumors may be retained). When known effusions are potential side effects of treatment (e.g., the use of certain taxane compounds or anti-angiogenic agents), cytological confirmation of the tumor origin of any effusion appearing or worsening during treatment may be considered to differentiate between disease remission (or disease stabilization) and PD where measurable tumor activity or disease stability is suggested.

Assessment of therapeutic effects.

Assessment of therapeutic efficacy in target lesions

• CR: All target lesions have disappeared. The short-axis diameter of all pathological lymph nodes, regardless of whether they are target lesions or not, must be <10 mm.

• PR: A reduction of at least 30% in the total sum of the diameters of all target lesions compared to the sum of the baseline diameters.

• PD: based on the sum of the smallest diameters in the study, the sum of target lesion diameters must increase by at least 20% (including baseline sum if it is the smallest diameter in the study). In addition to the relative 20%, the sum must also show an absolute increase of at least 5 mm (note: the appearance of one or more new lesions will also be considered progression).

• SD: Neither meets the criteria for Partial Response (PR) in terms of shrinkage nor for PD in terms of increase during the study period, serving as a reference for the sum of the smallest diameters.

• If the target lesion is, the actual short-axis value should always be measured and recorded (measured on the same anatomical plane as the baseline examination), even if the lesion being studied has regressed to below 10 mm. This means that, when also considered a target lesion, the "sum" of the lesions may not be zero, as normal is defined as short-axis <10 mm. In case reports, separately recorded, for compliance with CR, the short axis of each nodule must be <10 mm. For PR, SD, and PD, the actual short-axis measurement values of the nodules will be included in the total of the target lesion.

In studies of target lesions that are "too small to measure," all lesions (nodules and non-nodules) recorded at baseline should have their actual measurement values recorded in each subsequent evaluation, even if they are very small (e.g. 2 mm). However, sometimes lesions that were recorded as target lesions at baseline or are very blurry on CT scans to the point where radiologists cannot comfortably assign precise measurement values to them, and they may be reported as "too small to measure."

Recording a measurement value in the eCRF is important in situations like this. If the radiologist believes that the lesion may disappear, the measurement value should be recorded as 0 mm. If the lesion is believed to be present but is faintly visible and too small to measure, a default value of 5 mm should be specified (Note: this rule is unlikely to apply to lesions that typically have a definable size under normal circumstances and are often surrounded by fat, such as those in the retroperitoneum; however, if the lesion is believed to be present but faintly visible and too small to measure, a default value of 5 mm should be assigned in such cases as well). The default value is determined based on the thickness of a 5mm CT scan layer (if this thickness changes, the default value of 5 mm should not be altered). Measurement values of these (too small to measure) lesions may lack reproducibility, hence assigning this default value helps prevent false therapeutic or progression outcomes due to measurement errors. - It is important to reiterate that if the radiologist can provide an actual measurement value, even if it is less than 5mm, it should still be recorded.

• Treatment-related or fused lesions: When non-nodular lesions "shatter," the total length of all fragments must be added together to determine the sum of the target lesion (diameters). Similarly, if lesions fuse, the plane between the lesions before fusion may remain unchanged, which can aid in determining the maximum diameter of each lesion. If the lesions have indeed fully fused and will not separate further, the sagittal orientation of the longest diameter should be considered the longest diameter of the "fused lesion."

Assessment of efficacy in non-target lesions:

Although some non-target lesions may indeed be measurable, the specified time points in the study protocol may allow for qualitative assessment only, without measurements.

• CR: All non-target lesions have resolved and tumor marker levels have returned to normal. - All lesions must be non-pathological in size (i.e., short axis <10 mm).

• PD: There has been clear progression of non-target lesions (see details below). (Note: the appearance of one or more new lesions will also be considered as progression.)

• Non-CR/non-PD: One or more non-target lesions are present and/or tumor marker levels are maintained above the upper limit of normal.

• When a patient simultaneously suffers from measurable diseases: in this case, to achieve "clear progression" on the basis of non-target diseases, there must be an overall level of substantial deterioration in non-target diseases, resulting in a total tumor burden sufficient to discontinue treatment even if SD or PR is present in the target disease. One or more non-target lesions may show a slight increase in volume, but this is generally not enough to be classified as clear progression. Therefore, if the target disease shows SD or PR, determining overall progression solely based on changes in the target disease in this scenario would be extremely rare.

• When a patient only has unmeasurable disease: This situation typically occurs in some phase III trials, where having measurable disease excludes patients from enrolling in the study. In this context, the same general concepts apply, but in this scenario, the lack of assessment for measurable disease can be used to interpret the burden of unmeasurable disease. - Because worsening of non-targeted disease is not easily quantifiable (by definition: if all lesions are truly unmeasurable), useful assessments can be applied when evaluating if a patient has progressed definitively, by gauging the overall disease burden to the extent required for defining progression in measurable disease, based on changes in unmeasurable disease: i.e., an additional 73% "volume" of tumor burden (equivalent to a 20% increase in measurable lesion diameter). --- Examples of this would include pleural effusion going from "trace" to "significant," disease progressing from local to diffuse, or being described in a protocol as "sufficient to warrant treatment." If "definite progression" is observed, the patient should be classified as PD overall at that time point. While making objective assessments for unmeasurable disease is merely a conceptual state, significant magnitude must be observed due to the nature of the disease itself, for such an event to occur.

New lesion:

The emergence of a new malignant lesion indicates the occurrence of progressive disease, so it is very important to discuss the detection of new lesions. There is currently no specific method to identify new lesions on X-rays, but the finding of new lesions should be very clear, not attributed to differences in scanning techniques, imaging changes, or other findings apart from tumors (such as some "new" bone lesions that may simply be existing lesions healing or emerging). This is particularly important when the patient's baseline condition shows partial remission or complete remission. For example, liver necrosis may be reported as a "new" cystic lesion on a CT scan report, when in fact it is not.

If a lesion is detected in a follow-up study that was not found during baseline scanning, it can be considered a new lesion and indicates the occurrence of progressive disease. For example, a patient with baseline visceral disease who undergoes a CT or brain MRI during a study and is found to have metastatic lesions can be considered evidence of progressive disease, even if the patient did not have a brain imaging study at baseline.

If a new lesion is ambiguous, such as being too small, continued treatment and follow-up evaluations will determine whether it represents a true new disease. -If repeat scanning confirms the presence of a new lesion, the date of the initial scan should be considered the date of progression.

Overall evaluation of therapeutic effects:

Overall response refers to the documented efficacy from the beginning to the end of the therapeutic treatment, taking various factors into consideration. Sometimes the response is recorded only after the treatment has ended, so if post-treatment assessments need to be considered when determining overall efficacy, it should be specified in the protocol. The protocol must specify how the introduction of various new therapies prior to progression will impact efficacy. The overall response of patients is associated with the detection of target and non-target lesions, as well as the occurrence of new lesions. Additionally, due to the nature of the study itself and protocol requirements, specific measurements may need to be determined. Particularly in non-randomized studies, efficacy is a primary endpoint that requires determining PR or CR, where either one is considered an "overall response."

After obtaining all patient data, the overall response can be determined. In studies where confirmation of CR or PR is not required to determine efficacy, efficacy in these studies is defined as the efficacy at all time points (for example, if a patient is classified as SD at the first assessment, PR at the second assessment, and PD at the final assessment, their overall response would be categorized as PR). When disease stabilization is considered efficacy, it must also meet the minimum time from baseline as specified in the protocol. If the time for SD does not meet the specified minimum duration, but SD is indeed the efficacy at that time point, the patient's response will depend on subsequent assessments. For example, if a patient is assessed as SD at the first evaluation and then PD at the second, and the time for SD does not meet the minimum requirement, their response would be categorized as PD. If the same patient is lost to follow-up after the first assessment of SD, they will be considered unevaluable.

| **Target lesion** | **Non-target lesion** | **New lesion** | **overall relieve** |
| --- | --- | --- | --- |
| CR | CR | No | CR |
| CR | Non-CR/non-PD | No | PR |
| CR | Unrated | No | PR |
| PR | Non-PD or incomplete assessment | No | PR |
| SD | Non-PD or incomplete assessment | No | SD |
| Not fully evaluated. | Non-PD | No | NE |
| PD | Various | Yes or no | PD |
| Various | PD | Yes or no | PD |
| Various | Various | 是 | PD |

Abbreviations: CR, complete remission; NE, not evaluable; PD, progressive disease; PR, partial remission; SD, stable disease.

If nodular lesions are included in the target lesion sum, and if the nodules have decreased to "normal" size (<10 millimeters), measurements should still be reported in the scan records. Even if these nodules have resolved, these measurement results should be documented to not assess progression, and the basis should be whether the nodules are of the size. As mentioned earlier, this means that the sum of patients achieving a CR may not be "zero."

In research studies where determining efficacy is necessary, repeated assessments of "not evaluable" time points can make establishing efficacy more complex. The study protocol must specify how missing data/assessments will be used to determine efficacy or progression. For example, in most studies, considering PR-not evaluable-PR as a confirmed efficacy when evaluating efficacy at different time points is reasonable.

If a patient's overall health deteriorates and there is no objective evidence of progression disease at that time point, it should be reported as "symptomatic deterioration." Even after discontinuing treatment, efforts should be made to verify objective progression. Symptomatic deterioration is not a description of an objective response; it is the reason for stopping research treatment.

The definitions of "early progress," "early death," and "non-evaluability" are research-specific and should be clearly described in each protocol (depending on treatment timing and treatment cycles).

In some cases, it may be difficult to differentiate residual lesions from normal tissue. When relying on these results to evaluate CR, it is recommended to examine residual lesions (through needle aspiration/biopsy) before drawing conclusions on CR.

Due to the vague nature of the findings (such as very small and uncertain new lesions, cystic changes, and necrosis in existing lesions), treatment can be planned for the next scheduled assessment. If progression is confirmed at the next planned assessment, the date of progression should be earlier than the suspected date of progression.

**Confirmative Measurement/Mitigation Time**

Confirm:

In non-randomized studies with efficacy as the main endpoint, it is necessary to differentiate whether the efficacy observed in PR or CR is due to measurement error. Doing so will enable individuals to interpret the results appropriately based on historical data, as confirmation is required in such studies. However, in all other cases (such as randomized studies (Phase II or III) or studies with SD or progression as the main endpoint), confirming efficacy is not necessary as it does not add value to the interpretation of the study results. Nevertheless, eliminating the requirement for confirming efficacy may highlight the importance of preventing bias in central review, especially in non-blinded studies.

For SD, there should be at least one measurement of SD within the minimum interval after entering the study (typically not less than 6 weeks).

Overall relief time:

DOR is measured from the first assessment of complete response/partial response (taking the first recorded assessment as the reference) to the first day of objectively documented disease recurrence or progression (with the minimum recorded measurement in the study used as the reference for progressive disease).

The overall CR time is measured from the first CR achieved to the first day of objectively documented disease relapse.

Time of stabilization of the illness:

Measurement of time to disease stability is calculated from the start of treatment (from the day of enrollment) until progression is reached, taking the smallest time in the study as a reference (if the baseline total is the smallest, then the baseline value will be used as the reference for calculating PD).

The clinical relevance of disease stability time varies across different research and diseases. If the proportion of patients achieving disease stability in the shortest time possible is an important endpoint in a specific study, the protocol should specify the minimum time interval required for measuring disease stability on two occasions.

Note: Follow-up after baseline assessment will have an impact on DOR, time to disease stability, and PFS. - The definition of follow-up is beyond the scope of this guideline. - Many parameters should be considered, including disease type and stage, treatment duration, and practice. However, these limitations on measuring endpoint accuracy should be considered when comparing between studies

# References

1.Cohen, P.A., et al., Cervical cancer The Lancet, 2019. 393(10167): p. 169-182.

2. National Cancer Center Statistics,2022 .

3. ANGIOLO GADDUCCI and STEFANIA COSIO. ANTICANCER RESEARCH 40: 4819-4828 (2020)

4. Alessandro Buda, et al. Randomized trial of neoadjuvant chemotherapy comparing paclitaxel, ifosfamide, and cisplatin with ifosfamide and cisplatin followed by radical surgery in patients with locally advanced squamous cell cervical carcinoma: the SNAP01 (Studio Neo-Adjuvante Portio) Italian Collaborative Study. J Clin Oncol 23:4137-4145.

5. NCCN Guidelines Cervical Cancer Version 1. 2022

6. Minion, L.E. and K.S. Tewari, Cervical cancer - State of the science: From angiogenesis blockade to checkpoint inhibition. Gynecol Oncol, 2018. 148(3): p. 609-621.

7. Meng Ying et al. PD-L1 Expression Correlates With Tumor Infiltrating Lymphocytes And Response To Neoadjuvant Chemotherapy In Cervical Cancer. Journal of Cancer. 2018; 9(16): 2938-2945. doi: 10.7150/jca.22532

8. Thomas J. Herzog，et al. Gynecol Oncol 2015, 137, 204–205.

9. Richard S. Future Sci OA. Association of programmed death ligand 1 expression with prognosis among patients with ten uncommon advanced cancers. Mod Pathol. 2020 Aug 19;6(8):FSO616.

10. Richard S Clinicopathologic and genomic characterization of PD-L1-positive uterine cervical carcinoma. Mod Pathol. 2021 Jul;34(7):1425-1433.

11. Chung HC, Ros W, Delord JP, et al. Efficacy and Safety of Pembrolizumab in Previously Treated Advanced Cervical Cancer: Results From the Phase II KEYNOTE-158 Study . J Clin Oncol. 2019 Jun 10;37(17):1470-1478.

12. Oaknin A, Monk BJ, Vergote I, et al. 2021 ESMO, 783P.

13. Colombo N, Dubot C , Lorusso D, et al. 2021 ESMO, LBA2.

14. Peter Schmid et al. Pembrolizumab for Early Triple-Negative Breast Cancer.N Engl J Med. 2020 Feb 27;382(9):810-821.

15. Hongjing Jiang et al. KEYSTONE-001. 2021 ESMO IO

16. Clinical Trial.gov

17. Sanjeev Deva, et. al. ESMO IO, 2018

18. D Marinelli et al. KEAP1-driven co-mutations in lung adenocarcinoma unresponsive to immunotherapy despite high tumor mutational burden Annals of Oncology (2020) 31 (suppl_6): S1378-S1381. 10.1016/annonc/annonc365

19. A Phase II Study of tislelizumab plus chemotherapy in EGFR mutated advanced non-squamous NSCLC patients failed to EGFR TKI therapies: first analysis. ESMO IO 2021 148P

20. Chen Y, Hu J, Bu F, Zhang H, Fei K, Zhang P. Clinical characteristics of hyperprogressive disease in NSCLC after treatment with immune checkpoint inhibitor: a systematic review and meta-analysis .BMC Cancer. 2020 Jul 29;20(1):707

21. D.Wang et.al. SGO 2022.
